# Color-tunable luminescent Tb_x_Eu_y_(BDC) complexes assembled within liposome-based nanoreactors

**DOI:** 10.1016/j.xcrp.2026.103312

**Published:** 2026-05-20

**Authors:** Aaron Torres-Huerta, Miriam de J. Velásquez-Hernández, Sven Lempereur, Ludovic Troian-Gautier, Giulia Veronesi, Hennie Valkenier

**Affiliations:** 1 Université libre de Bruxelles, Engineering of Molecular NanoSystems, Avenue F.D. Roosevelt 50, CP165/64, 1050 Brussels, Belgium; 2 Center for Membrane Separations, Adsorption, Catalysis, and Spectroscopy (cMACS), KU Leuven, 3001 Leuven, Belgium; 3 Institut de la Matière Condensée et des Nanosciences (IMCN), Molecular Chemistry, Materials and Catalysis (MOST), Université catholique de Louvain (UCLouvain), 1348 Louvain-la-Neuve, Belgium; 4 WEL Research Institute, 6 Avenue Pasteur, 1300 Wavre, Belgium; 5 University Grenoble Alpes, CNRS, CEA Grenoble, IRIG, Laboratoire de Chimie et Biologie des Métaux, 17 Rue des Martyrs, 38000 Grenoble, France

**Keywords:** ion transport, multimetallic lanthanides, color tunability, luminescence, liposomes, nanoreactors

## Abstract

Precise stoichiometric control in multimetallic lanthanide nanosystems is essential for optical devices, sensing, and bioimaging applications owing to their composition-dependent emission properties. However, controlling metal composition, spatial distribution, intermetallic energy transfer, and colloidal stability remains challenging. Here, we report a liposome-based nanoreactor platform that enables the *in situ* formation of multivariate Tb_x_Eu_y_-dicarboxylate complexes, enabling finely tuned lanthanide stoichiometry within attoliter-scale confined volumes. Liposomes pre-loaded with specific Tb^3+^:Eu^3+^ ratios are combined with a synthetic anion transporter that mediates dicarboxylate transport through lipid membranes, enabling controlled coordination reactions in aqueous solution. This method, coupled with the use of a blue-emissive ligand, supports continuous color tuning across the entire RGB spectrum. Real-time emission spectroscopy reveals faster photoluminescence appearance for Eu^3+^ than Tb^3+^, providing experimental insight into lanthanide reactivity under nanoscale confinement. These findings position liposome-based nanoreactors as a versatile platform for investigating coordination reactions and engineering multimetallic luminescent colloidal materials in aqueous media.

## Highlights

• Liposome nanoreactors enable coordination reactions with controlled Tb/Eu ratios
• RGB emission color tuning is achieved via ligand and metal composition control
• Colloidal emissive systems are stable in aqueous solutions

## Graphical abstract

**Figure:**
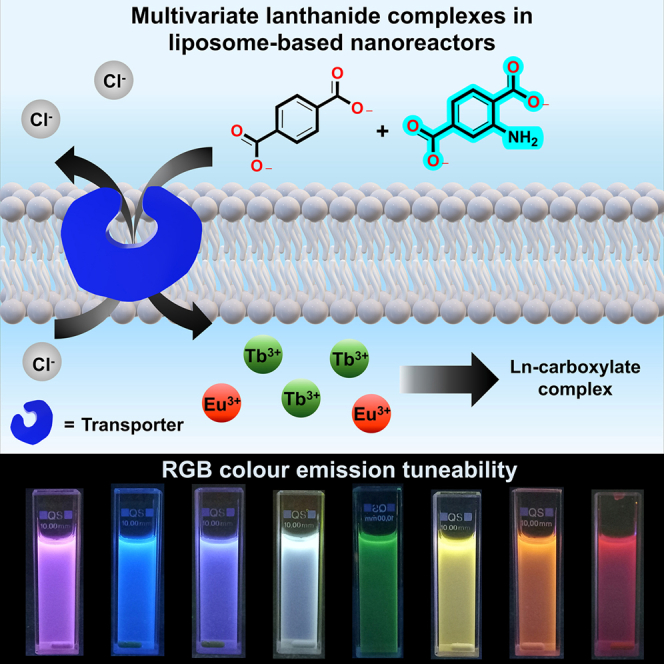

## Teaser

Aaron Torres-Huerta et al. develop liposome nanoreactors that assemble multimetallic lanthanide complexes inside attoliter compartments. Real-time photoluminescence monitoring reveals distinct Tb^3+^ and Eu^3+^ emission behavior, while ligand engineering enables RGB color tuning, providing a platform for studying coordination chemistry and designing luminescent nanomaterials in aqueous environments.

## Introduction

Lanthanide-based systems possess exceptional luminescent properties, making them uniquely suited for optical applications.^1^^,^^2^^,^^3^ These properties arise from the open-shell 4f orbitals of lanthanides, resulting in narrow emission bands, high color purity, and long luminescence lifetimes. Precise control over emission color is essential for advancing a wide range of emerging technologies, including information encryption,^4^^,^^5^ light-emitting diodes (LEDs),^6^ optical thermometers,^7^ sensing,^8^ and bioimaging.^2^^,^^9^

The rational integration of multiple lanthanide ions, such as terbium (Tb^3+^) and europium (Eu^3+^), into a single system enables multicolor emission spanning the visible spectrum.^10^ However, the formation of multivariate lanthanide complexes remains challenging, and the major limitation lies in the difficulty of distinguishing genuine heterometallic assemblies from physical mixtures or phase-segregated domains.^11^ Moreover, intermetallic energy transfer from Tb^3+^ to Eu^3+^, commonly observed in solid-state materials such as metal-organic frameworks (MOFs),^12^ often leads to quenching of Tb-based green emission, compromising color tunability and spectral precision. Furthermore, the stoichiometric ratios of lanthanide precursors do not always reflect the final product composition,^13^ introducing further variability and uncertainty in the design of multimetallic emitters. To mitigate undesired energy transfer and improve control over emission profiles, strategies such as core-shell nanoparticles,^14^^,^^15^^,^^16^ core-shell MOFs,^13^^,^^17^^,^^18^^,^^19^ and layer-by-layer films^20^^,^^21^ have been employed to spatially isolate different lanthanides. Although effective, these approaches often involve sophisticated fabrication protocols and are generally limited to solid-state systems, hindering their use in aqueous or biologically relevant environments.

To address these challenges and enable the formation of water-compatible, luminescent nanomaterials with color-tunable properties, we propose the on-demand assembly of lanthanide-carboxylate complexes within liposomes. Drawing inspiration from synthetic ion transport strategies commonly applied in the context of channelopathies,^22^^,^^23^ we have pioneered the controlled transmembrane transport of organic ligands into liposomes pre-loaded with selected metal ions to drive the formation of hybrid metal-organic colloidal materials.^24^

**Figure 1:**
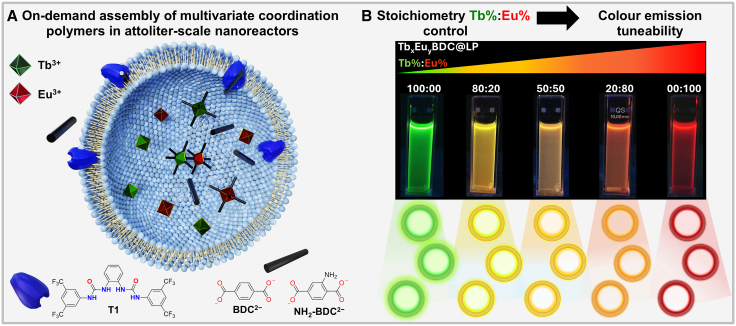
Liposome nanoreactor strategy to control multivariate lanthanide complex formation (A) Schematic illustration of the liposome-based nanoreactor (LNR) strategy, showing the controlled transmembrane transport of benzene-1,4-dicarboxylate (BDC^2−^) and subsequent coordination with encapsulated lanthanide cations inside 200-nm liposomes. (B) Tuning of Tb^3+^:Eu^3+^ ratios to achieve color-tunable luminescent nanomaterials in aqueous solution.

In this work, we exploit the liposome-based nanoreactor (LNR) platform to tune the luminescent properties of colloidal multivariate Tb_x_Eu_y_(BDC) complexes assembled within attoliter (10^−18^ L) confined volumes (Tb_x_Eu_y_BDC@LP; LP, liposome). This level of synthetic control is achieved by encapsulating predefined Tb^3+^:Eu^3+^ ratios in liposomes, followed by the assisted transport of benzene-1,4-dicarboxylate (BDC^2−^) using a bisurea-based transporter (**T1**), to afford colloidal Tb_x_Eu_y_BDC@LP systems with a bilamellar structure (Figures 1 and S2; Note S1). This methodology offers precise control over the stoichiometry ratios of encapsulated lanthanide ions, and the fluorescence properties of the lanthanides allow for real-time monitoring via fluorescence spectroscopy. The successful formation of co-encapsulated Tb_x_Eu_y_BDC@LP systems can be confirmed by steady-state photoluminescence spectroscopy using the distinct emission profiles of both lanthanides. Real-time tracking of the 542 (from Tb^3+^) and 614 (from Eu^3+^) nm emission bands allows us to study the emission evolution kinetics of Eu^3+^ and Tb^3+^ in competitive mixtures, providing direct experimental insight into the reactivity of lanthanides in confined nanospaces. Furthermore, by employing the blue-emissive 2-amino-1,4-benzenedicarboxylate (NH_2_BDC^2−^) ligand, the system could be extended to achieve full RGB color tunability.

## Results and discussion

### Preparation and characterization of luminescent multivariate Tb_x_Eu_y_BDC@LP nanosystems

Liposomes were prepared using a 7:3 molar ratio of 1-palmitoyl-2-oleoyl-*sn*-glycero-3-phosphocholine (POPC) and cholesterol. The lipid film was hydrated with a 7 mM aqueous solution of TbCl_3_ and EuCl_3_ in predefined molar ratios (Tb:Eu = 100:00, 90:10, 80:20, 70:30, 60:40, 50:50, 40:60, 30:70, 20:80, 10:90, and 00:100). Then, lipids were subjected to freeze-thaw cycles and extrusion through a polycarbonate membrane with 200 nm pores to afford uniform Tb_x_Eu_y_Cl_3_@LP vesicles. Non-encapsulated lanthanide ions and chloride were removed by dialysis against a 10.5 mM Na_2_SO_4_ solution. The resulting vesicle suspensions were diluted in Na_2_SO_4_ medium to achieve a final lipid concentration of 3 mM.

The encapsulation efficiency was confirmed using a chloride ion-selective electrode (Cl-ISE) by measuring the chloride concentration in a 3 mL liposomal suspension. All Tb_x_Eu_y_Cl_3_@LP samples displayed residual chloride levels below 0.07 mM. After lysing the liposomes with Triton X-100, the chloride concentration increased significantly (0.36–0.45 mM; Figure S1; Methods S1), which corresponds to an estimated total lanthanide concentration of 0.12–0.15 mM. This confirmed that chloride and, consequently, the lanthanide ions were successfully encapsulated.

**Figure 2:**
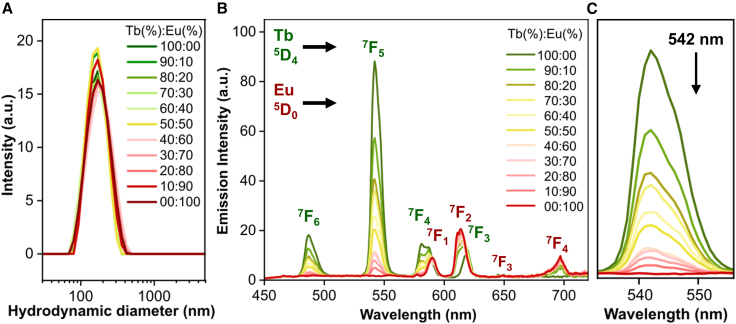
Characterization of Tb_x_Eu_y_BDC@LP systems (A) DLS measurements of Tb_x_Eu_y_BDC@LP samples in aqueous Na_2_SO_4_ solution. (B) Emission spectra of Tb_x_Eu_y_BDC@LP samples upon excitation at 285 nm, displaying characteristic terbium and europium transitions. (C) Zoom of the emission band around 542 nm as a function of terbium mole fraction.

To induce the *in situ* formation of lanthanide-organic complexes within the liposomes, BDC^2−^ was transported through the membrane by the anion transporter **T1** (Figure 1A) at a transporter-to-lipid molar ratio of 1:10,000. The integration of **T1** in the lipid membrane triggers the dissipation of the BDC^2−^ gradient via the BDC^2−^/Cl^−^ ion-exchange process.^25^ Dynamic light scattering (DLS) measurements confirmed that the resultant Tb_x_Eu_y_BDC@LP systems exhibit a monodisperse size distribution and high colloidal stability, with an average hydrodynamic diameter of approximately 170 nm (Figures 2A and S3), which is consistent with our previous reports.^24^ Additionally, scanning electron microscopy coupled with energy-dispersive X-ray spectroscopy (SEM-EDX; Methods S2) analysis of selected samples revealed the co-localization of both Tb^3+^ and Eu^3+^ within individual liposomes (Figure S4).

The successful formation of the luminescent Tb_x_Eu_y_(BDC) complex inside the liposomes was confirmed by steady-state photoluminescence spectroscopy in solution. Emission spectra were recorded from 450 to 750 nm upon excitation at λ_ex_ = 285 nm, using a 450 nm long-pass filter. The excitation wavelength was selected based on the excitation spectra of reference samples Tb_100_Eu_00_BDC@LP and Tb_00_Eu_100_BDC@LP (Figure S5). The Tb_100_Eu_00_BDC@LP sample displayed characteristics terbium transitions ^5^D_4_→^7^F_j_ (j = 6, 5, 4, and 3), while the Tb_00_Eu_100_BDC@LP sample exhibited europium transitions ^5^D_0_→^7^F_j_ (j = 1–4), which are consistent with carboxylate coordination (Figures S6 and S8).^26^ Samples containing both Tb^3+^ and Eu^3+^ ions displayed both sets of transitions, in agreement with the co-encapsulation and implication of both lanthanides in the coordination reaction with BDC^2−^ (Figures 2B and S9).

Notably, the terbium transitions ^5^D_4_→^7^F_j_ exhibited higher emission intensity than the europium transitions ^5^D_0_→^7^F_j_. This trend is consistent with the generally more efficient sensitization of Tb^3+^ by BDC^2−^ ligands than of Eu^3+^, as reported for related lanthanide-carboxylate systems.^27^ To further support this interpretation, we measured the photoluminescence quantum yields of representative samples (see Methods S3). The Tb_100_Eu_00_BDC@LP, Tb_00_Eu_100_BDC@LP, and Tb_50_Eu_50_BDC@LP systems exhibited quantum yields of Φ = 0.049, 0.016, and 0.011, respectively. Interestingly, the strongest Tb^3+^ emission at 542 nm (^5^D_4_→^7^F_5_) significantly decreased with increasing Eu^3+^ contents (Figure 2C), consistent with quenching via intermetallic energy transfer from Tb^3+^ to Eu^3+^ (Figure S6). This behavior is in line with previously reported Tb^3+^→Eu^3+^ energy transfer in mixed-lanthanide MOFs^12^^,^^28^ and molecular lanthanide systems.^29^

### Comparison of the emission properties of co-encapsulated Tb_x_Eu_y_BDC@LP systems and controlled mixtures of monometallic systems

To gain deeper insights into the intermetallic energy transfer from Tb^3+^ to Eu^3+^ in multivariate systems, we also prepared physical mixtures of Tb_100_Eu_00_BDC@LP and Tb_00_Eu_100_BDC@LP samples in mole fractions that matched the Tb:Eu ratios used in the Tb_x_Eu_y_BDC@LP series. These mixtures ensured equivalent total lanthanide concentrations but maintained spatial separation between Tb^3+^ and Eu^3+^ within distinct liposomes, thereby minimizing direct intermetallic interactions.

**Figure 3:**
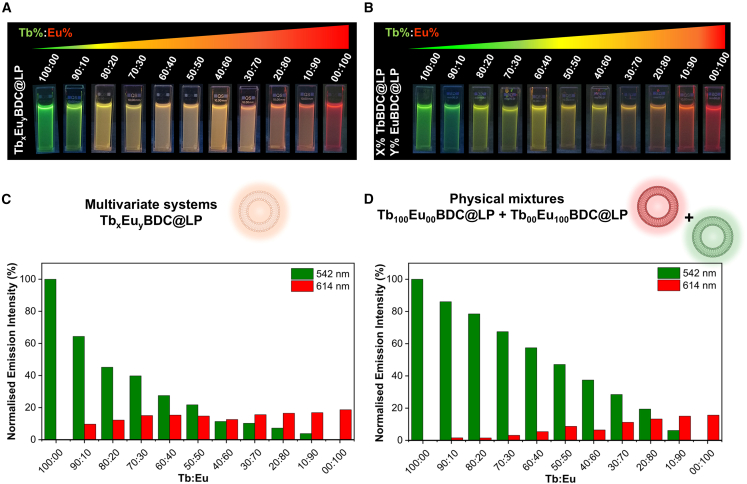
Emission properties of co-encapsulated lanthanides and physically mixed systems (A and B) Photographs of liposome suspensions under 254-nm UV illumination, showing tunable emission colors in (A) the Tb_x_Eu_y_BDC@LP series and (B) their analog physical mixtures. (C) Emission intensities for Tb_x_Eu_y_BDC@LP samples collected at 542 (Tb^3+^, green) and 614 (Eu^3+^, red) nm reveal an exponential decrease in terbium emission and a corresponding increase in europium emission with rising Eu^3+^ content. (D) Emission intensities for physical mixtures of Tb_100_Eu_00_BDC@LP and Tb_00_Eu_100_BDC@LP at equivalent Tb:Eu mole fractions for reference.

Under UV illumination at 254 nm, these differences were visually apparent. The co-encapsulated Tb_x_Eu_y_BDC@LP samples, in which intermetallic energy transfer is active, exhibited a color gradient from green (Tb rich) to red (Eu rich), with prominent yellow to orange hues observed at intermediate compositions (Tb:Eu = 80:20–10:90) (Figure 3A). In contrast, the physically mixed systems, which maintained spatial separation between lanthanides and thereby suppressed energy transfer, displayed predominantly green-yellow emission at Tb:Eu ratios of 100:00–40:60 (Figure 3B), with significantly reduced orange-red colors.

Quantitative analysis of the emission intensities at 542 (from Tb^3+^) and 614 (from Eu^3+^) nm revealed pronounced differences between co-encapsulated (Figure 3C) and physically mixed (Figures 3D and S10) systems. In the Tb_x_Eu_y_BDC@LP series, terbium emission showed an exponential decrease, while europium emission increased logarithmically with increasing Eu^3+^ mole fraction (Figures 3C and S11). This behavior is consistent with the intermetallic energy transfer from Tb^3+^ to Eu^3+^ within the confined environment of the liposomes. In contrast, physical mixtures displayed linear emission trends for both lanthanides (Figures 3D and S12). This linearity indicates a simple dilution effect rather than an energy transfer process, suggesting the presence of independent, non-interacting luminescent species.

These findings demonstrate that spatial confinement within liposomes enables controlled localization and distribution of lanthanide ions, thereby governing intermetallic energy transfer and resulting in tunable luminescence. Notably, while co-encapsulation promotes energy-transfer-based modulation, physical mixtures yield more predictable and finely tunable color due to the absence of intermetallic coupling. Overall, the liposomal membrane functions as a versatile nanoscale scaffold that supports either synergistic or independent luminescent emission, depending on the spatial arrangement of the lanthanide ions.

### Monitoring lanthanide emission appearance in lipid nanoreactors with real-time steady-state photoluminescence spectroscopy

To further assess changes in the emission behavior of Tb^3+^ and Eu^3+^ within the LNRs, we performed real-time steady-state photoluminescence measurements. Emission intensities at 542 (Tb^3+^, ^5^D_4_→^7^F_5_) and 614 (Eu^3+^, ^5^D_0_→^7^F_2_) nm were simultaneously recorded over 30 min, upon the addition of BDC^2−^. These measurements allowed us to monitor both the transmembrane transport of BDC^2−^ within the vesicle interior and the subsequent linker coordination with the encapsulated lanthanide ions.

**Figure 4:**
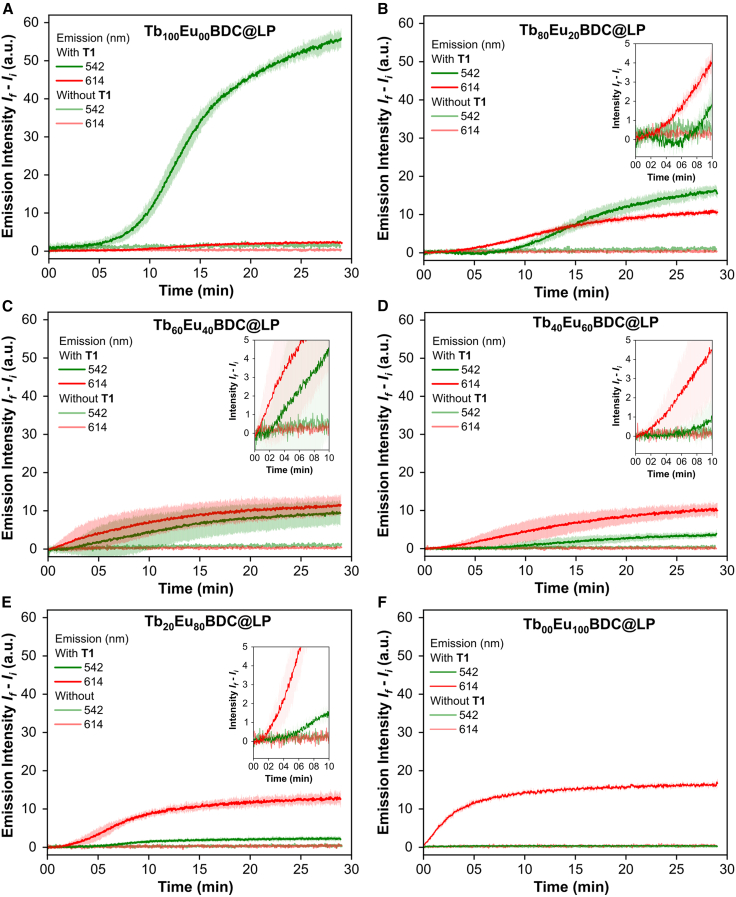
Real-time monitoring of emission changes following BDC^2−^ addition (A–F) Emission intensities at 542 nm (green line) correspond to the Tb^3+ 5^D_4_→^7^F_5_ transition, while those at 614 nm (red line) correspond to the Eu^3+ 5^D_0_→^7^F_2_ transition. Kinetic data were collected for (A) Tb_100_Eu_00_BDC@LP, (B) Tb_80_Eu_20_BDC@LP, (C) Tb_60_Eu_40_BDC@LP, (D) Tb_40_Eu_60_BDC@LP, (E) Tb_20_Eu_80_BDC@LP, and (F) Tb_00_Eu_100_BDC@LP. Each image includes control experiments without transporter **T1**, represented by light green and light red. Shaded areas represent the standard deviation of three independent measurements.

Initial experiments were performed on the single-lanthanide reference samples Tb_100_Eu_00_BDC@LP and Tb_00_Eu_100_BDC@LP, using **T1** at a 1:10,000 transporter-to-lipid molar ratio. Upon BDC^2−^ addition, the europium-containing sample exhibited an immediate increase in emission intensity at 614 nm, indicating rapid complex formation (Figure 4F). In contrast, the terbium-containing sample displayed a delayed onset emission at 542 nm, with a pronounced increase occurring ∼5 min post-addition (Figure 4A). These differences could, for instance, be due to intrinsic variations in the coordination kinetics of Eu^3+^ and Tb^3+^ with carboxylate ligands or to different thresholds in local coordination geometry required for each lanthanide to become efficiently emissive.

In multivariate systems such as the Tb_80_Eu_20_BDC@LP sample (Figure 4B), the initial emission was dominated by the Eu^3+^ band centered at 614 nm, which increased faster and reached a plateau earlier than the emission associated with Tb^3+^ at 542 nm. Thus, despite the higher Tb^3+^ mole fraction, europium emission clearly appeared faster. This trend persisted across all Tb_x_Eu_y_BDC@LP samples (Figures 4B–4E and S13–S18), where the emission of europium increased consistently at a faster rate than that of terbium. Photoluminescence data across the Tb_x_Eu_y_BDC@LP series revealed a systematic reduction in the Tb^3+^ signal intensity and a corresponding increase in the Eu^3+^ signal as the mole fraction of Eu^3+^ increased. Furthermore, the presence of quenching throughout the kinetic profiles of Tb_x_Eu_y_BDC@LP systems suggests the formation of a multimetallic coordination complex.

The earlier appearance of Eu^3+^ compared to Tb^3+^ emission may arise not only from faster coordination kinetics, as in the present system, the time-dependent photoluminescence response reflects the convolution of several coupled processes. These include the evolution of ligand-to-metal sensitization pathways and structural rearrangements of the coordination assemblies to obtain strong emission. To understand such contributions in a quantitative manner would require detailed theoretical modeling and/or advanced kinetic analysis, which are beyond the scope of the present work.

Control experiments conducted in the absence of **T1** confirmed that no change in emission intensity occurred upon BDC^2−^ addition, supporting the role of **T1** in actively mediating ligand transport and indicating the integrity of the liposomal membrane, confirming the absence of Ln^3+^ leakage and the impermeability of the lipid membrane to BDC^2−^ in the absence of an anion transporter.

We have attempted to study the changes in the coordination of Tb^3+^ during the formation of Tb_100_Eu_00_BDC@LP systems in more detail using X-ray absorption spectroscopy (XAS; see Methods S4 and Note S3). While the Tb^3+^ signal was clearly observed, the changes in the coordination sphere were too small to result in significant differences in the near-edge region of the spectra (Figure S29A), and only minor differences were detected in the extended X-ray absorption fine structure (EXAFS) spectra (Figures S29B and S30; Table S1). This can be explained by the first coordination sphere most likely being made up exclusively of oxygen atoms throughout the coordination process. At the start, the encapsulated Tb^3+^ will be solvated by water molecules, with potential interactions with phosphate groups from the lipids, and after the transport of BDC^2−^ into the liposomes, the water molecules are (partially) replaced by the coordinating carboxylate groups. The coordination of the carboxylates to Tb^3+^ or Eu^3+^ cations in the liposomes was confirmed by infrared spectroscopy (see Methods S5; Note S4; Figures S31 and S32).

### Extending emission to cover the RGB spectrum

To expand the potential versatility of our system, we used the blue-emissive ligand NH_2_BDC^2−^ to achieve full RGB color coverage, which represents an essential feature for advanced display technologies and optical probes within biological environments.^30^^,^^31^ NH_2_BDC^2−^ is known to exhibit blue fluorescence (∼460–480 nm; Figure S19) in ligand-based MOF systems,^32^^,^^33^ making it a suitable complement to the green (Tb^3+^) and red (Eu^3+^) emissions within our nanoreactor platform.

Initial attempts to form Tb(NH_2_BDC)@LP and Eu(NH_2_BDC)@LP resulted exclusively in intense blue emission, with no detectable Tb^3+^ or Eu^3+^ transitions, indicating that the NH_2_BDC ligand strongly quenches lanthanide-based emission. This behavior is consistent with previous reports on Ln-NH_2_BDC-based MOFs suspended in dry organic solvents, where even small amounts of water (<5%) cause significant quenching of lanthanide emission due to Ln^3+^→ligand energy back-transfer processes.^34^^,^^35^ To minimize quenching, the excess of unbound ligand was removed by introducing an additional post-complexation dialysis step for the Ln(NH_2_BDC)@LP and Ln(BDC)(NH_2_BDC)@LP systems (see the methods section).

**Figure 5:**
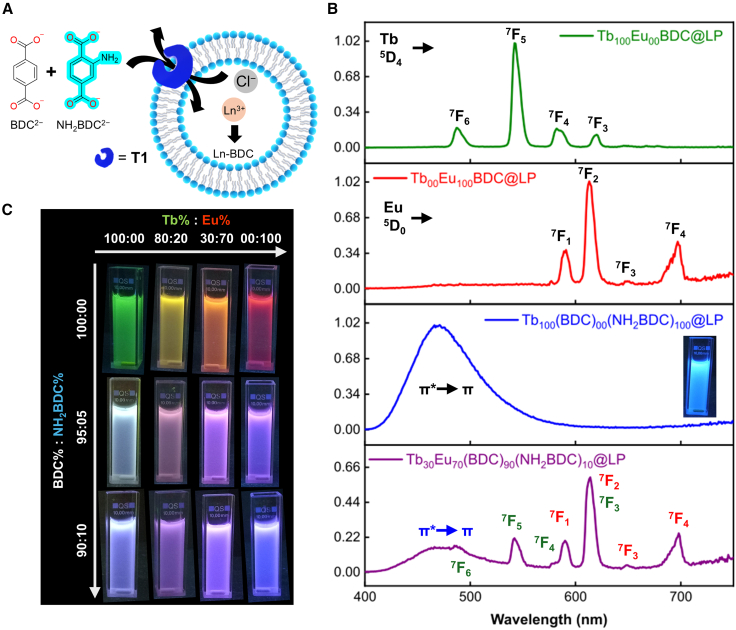
RGB emission tuning using mixed-ligand systems (A) Extension of emission tunability to cover the full RGB spectrum through the use of mixed BDC^2−^/NH_2_BDC^2−^ ligand pulses. (B) Emission spectra of representative systems: Tb_100_Eu_00_BDC@LP, Tb_00_Eu_100_BDC@LP, Tb_100_(BDC)_00_(NH_2_BDC)_100_@LP, and Tb_30_Eu_70_(BDC)_90_(NH_2_BDC)_10_@LP. (C) Mixed-ligand systems generated by replacing the ligand pulse with BDC^2−^/NH_2_BDC^2−^ mixtures (95:05 and 90:10), expanding chromaticity. Photographs are of liposome suspensions under 254-nm UV illumination.

Mixed-ligand systems with varying BDC^2−^:NH_2_BDC^2−^ mole fractions were then prepared (Figure 5A). Compositions containing ≥20% NH_2_BDC^2−^ significantly suppressed lanthanide emission (Figures S21 and S22), whereas samples containing ≤10% NH_2_BDC^2−^ retained characteristic Tb^3+^ and Eu^3+^ bands. Based on these results, selected reference compositions exhibiting green (Tb_100_Eu_00_BDC@LP), yellow (Tb_80_Eu_20_BDC@LP), orange (Tb_30_Eu_70_BDC@LP), and red (Tb_00_Eu_100_BDC@LP) emissions were further modified by replacing the ligand pulse with BDC^2−^:NH_2_BDC^2−^ mixtures at 95:05 and 90:10 ratios (Figures 5B and 5C). In all cases, a reduction in lanthanide emission intensity was observed, more pronounced for Tb^3+^ than Eu^3+^ (Figures S23–S26), while blue emission from NH_2_BDC^2−^ became dominant. The resulting chromaticity enabled the acquisition of additional colors, such as gray-blue (Tb_100_Eu_00_(BDC)_95_(NH_2_BDC)_05_@LP) and pink (Tb_80_Eu_20_(BDC)_95_(NH_2_BDC)_05_@LP). Although the additional post-complexation dialysis step and Fourier transform infrared (FTIR) measurements (Figures S31 and S32) support coordination of NH_2_BDC^2−^ to the lanthanide cations, the confined and dynamic environment inside liposome nanoreactors may allow the coexistence of multiple coordination species. Therefore, the present experiments cannot completely exclude the presence of mixed assemblies or minor fractions of uncoordinated ligand. Additionally, DLS analysis confirmed that the introduction of NH_2_BDC^2−^ did not affect colloidal integrity, as all samples remained monodisperse with hydrodynamic diameters comparable to the Tb_x_Eu_y_BDC@LP samples (Figures S21B and S22B).

Furthermore, to explore whether spatial separation could mitigate quenching, physical mixtures were prepared using Tb_100_Eu_00_BDC@LP and the highly violet-emissive Tb_00_Eu_100_(BDC)_95_(NH_2_BDC)_05_@LP. As anticipated, the liposomal membrane prevented detrimental quenching of the Tb^3+^ transitions, and the 542 nm emission band decreased linearly with the volume fraction of the violet-emissive partner, consistent with a dilution effect (Figure S27). This mixing strategy enabled a controlled color shift from green to cyan and ultimately to near-white emission at a 70:30 ratio (Figure S28), demonstrating precise RGB balance through nanoscale spatial control.

In summary, this study establishes LNRs as a powerful and versatile platform for the synthesis and study of multimetallic lanthanide complexes in aqueous conditions. By precisely controlling the Tb^3+^:Eu^3+^ stoichiometry and spatial co-localization within attoliter-scale vesicles, we achieved the *in situ* formation of Tb_x_Eu_y_(BDC) complexes with composition-dependent, tunable emission profiles. The use of a synthetic bisurea as an anion transporter enabled controlled ligand delivery and real-time steady-state photoluminescence monitoring, revealing that Eu^3+^ exhibits faster emission increases than Tb^3+^ under nanoscale confinement. Comparative studies with physically mixed monometallic systems confirmed that efficient Tb→Eu energy transfer requires co-encapsulation and demonstrated the suitability of LNRs to resolve subtle differences in reactivity and complexation. Extending this strategy to blue-emissive NH_2_BDC^2−^ ligands enables full RGB coverage, expanding the photonic versatility of the platform. Notably, the liposomal membrane ensures colloidal stability, prevents aggregation, and provides a biologically relevant aqueous environment. Overall, this study highlights LNRs as an adaptable nanoscale scaffold for engineering multimetallic luminescent nanomaterials with precisely controlled spatial organization and optical output.

## Methods

### General experimental information

The reagents and solvents were obtained from Sigma-Aldrich and used without further purification unless otherwise specified. POPC and cholesterol were purchased from Sigma-Aldrich and Acros, respectively. Chloroform was deacidified before preparing lipid solutions by passing it through a column containing basic alumina. POPC solutions were stored at −20°C, while cholesterol solutions were freshly prepared. All aqueous solutions were prepared using deionized water, and the pH values were 6.5–7.

### Liposome preparation

Liposomes were prepared from a lipid mixture of POPC and cholesterol at a 7:3 molar ratio, dissolved in deacidified chloroform. The total lipid amount was calculated to achieve a final concentration of 3 mM (POPC + cholesterol). Chloroform was removed by evaporation under a gentle stream of nitrogen, followed by overnight drying under vacuum to ensure complete solvent removal. The dried lipid film was rehydrated with 500 μL of a 7 mM aqueous solution containing TbCl_3_ and EuCl_3_ in predefined molar ratios (Tb:Eu = 100:00, 90:10, 80:20, 70:30, 60:40, 50:50, 40:60, 30:70, 20:80, 10:90, and 00:100). The resulting suspension was sonicated for 1 min and stirred for 1 h at room temperature to promote vesicle formation. To obtain predominantly unilamellar vesicles, the suspension underwent 10 freeze-thaw cycles, followed by extrusion 29 times through polycarbonate membranes with a pore size of 200 nm at room temperature.

Unencapsulated lanthanide chloride (LnCl_3_) outside the liposomes was removed by dialysis using Biotech CE Tubing dialysis membranes (molecular weight cut-off [MWCO] 20 kDa) against a 10.5 mM Na_2_SO_4_ solution. The final LnCl_3_@LP suspension was then diluted in the external solution to achieve a final lipid concentration of 3 mM for fluorescence experiments.

### Liposome preparation for RGB systems

Liposomes were prepared following the methodology described above, with modifications introduced to minimize the quenching effect of the NH_2_BDC^2−^ ligand. Specifically, the total concentration of the BDC^2−^:NH_2_BDC^2−^ mixture was reduced to 0.33 mM, and the resulting Ln(BDC)(NH_2_BDC)@LP suspensions were diluted in the external solution to achieve a final lipid concentration of 1 mM. The concentration of transporter **T1** was maintained at a transporter-to-lipid molar ratio of 1:10,000. To remove excess unbound ligand remaining in the external phase after complex formation, a post-complexation purification step was included, consisting of dialysis against a 10.5 mM Na_2_SO_4_ solution using Biotech CE Tubing (MWCO 20 kDa) (three cycles, 150 mL each).

### DLS

DLS measurements were carried out using a Malvern Zetasizer Ultra instrument at 25°C. Samples were loaded into disposable cuvettes, and measurements were performed using a standard refractive index of 1.45 for lipid-based systems.

### Steady-state photoluminescence spectroscopy

Steady-state photoluminescence measurements were carried out using a Horiba Fluoromax-4. Emission spectra were recorded from 450 to 750 nm upon excitation at 285 nm, using a 450 nm long-pass filter (Figure S20; Note S2). All measurements were performed in quartz cuvettes containing a magnetic stir bar. The cuvette holder temperature was maintained at 25°C using a water-circulating bath, and the sample compartment was allowed to equilibrate for 3 min before each experiment.

For complexation experiments, 3 mL of a LnCl_3_@LP suspension was placed in the cuvette, and **T1** (3.3 μL, 0.27 mM in methanol) was added to achieve a transporter-to-lipid molar ratio of 1:10,000. Then, 60 μL of a 50 mM BDC^2−^ solution was added, resulting in a final BDC^2−^ concentration of 1 mM. Emission spectra were recorded until no further changes in intensity were observed, typically within 1 h. Additionally, control experiments were conducted to verify that the emission results directly from the coordination of BDC^2−^ with the lanthanide cation inside liposomes (Figure S7). For kinetics experiments, the emission intensities at 542 and 614 nm were recorded simultaneously over a 30-min period following the addition of BDC^2−^ and **T1**.

### Photographs

Photographs of the liposomal suspensions were captured using a Samsung A4 smartphone under illumination from a handheld UV lamp (254 nm). The images were taken inside a custom-built wooden black box to eliminate ambient light interference.^36^^,^^37^^,^^38^^,^^39^^,^^40^^,^^41^^,^^42^^,^^43^

## Resource availability

### Lead contact

Requests for further information and experimental data should be directed to the lead contact, Hennie Valkenier (hennie.valkenier@ulb.be).

### Materials availability

This study did not generate new unique reagents.

### Data and code availability

• All data reported in this paper will be shared by the lead contact upon request.
• This paper does not report original code.
• Any additional information required to reanalyze the data reported in this paper is available from the lead contact upon request.

## Acknowledgments

This project has received funding from the European Union’s Horizon Europe research and innovation programme under Marie Skłodowska-Curie grant agreement no. 101065037 and from the 10.13039/501100000781European Research Council (ERC) under grant agreement no. 802727 (Horizon 2020). Access to international research center laboratories and large-scale analytical facilities was supported by the European Union’s Horizon 2020 research and innovation programme under grant agreement no. 101007417, having benefited from the access provided by the SAMBA station at the SOLEIL synchrotron in Paris, France, and ENL FEMTO-ST/10.13039/501100004794CNRS - EuroNanoLab in Besançon, France, within the framework of the NFFA-Europe Pilot Transnational Access Activity, proposal ID520. We extend our gratitude to Dr. Andrea Zitolo for assisting with XAS data acquisition and to Elena Tamarit-Amoros for her support in sample preparation during synchrotron experiments. This work was partly supported by the French RENATECH network and its FEMTO-ST technological facility. We thank Marina Raschetti for her assistance with the acquisition and analysis of SEM-EDX data. H.V. is a research associate of the Fonds de la Recherche Scientifique (FNRS).

## Author contributions

A.T.-H., M.d.J.V.-H., and H.V. conceptualized the study and designed the experiments. G.V. supervised, analyzed, and validated the synchrotron experiments and results. S.L. and L.T.-G. determined the photoluminescence quantum yields. A.T.-H. and M.d.J.V.-H. prepared the initial draft of the manuscript. H.V. reviewed and edited the manuscript. H.V. provided supervision and project administration. Funding acquisition was secured by H.V. and A.T.-H. All authors reviewed and approved the final version of the manuscript.

## Declaration of interests

The authors declare no competing interests.

## Supplemental information

Document S1. Figures S1–S32, Table S1, Notes S1–S4, and Methods S1–S5

Document S2. Article plus supplemental information
