# Supplementary material for: Color-tunable luminescent TbxEuy(BDC) complexes assembled within liposome-based nanoreactors
Source: Cell Rep Phys Sci. 2026 May 20;7(5):103312. doi: 10.1016/j.xcrp.2026.103312 (PMC13190561; doi:10.1016/j.xcrp.2026.103312)
Supplement: Document S1. Figures S1–S32, Table S1, Notes S1–S4, and Methods S1–S5 [file mmc1.pdf]

Cell Reports Physical Science, Volume 7

Supplemental information

Color-tunable luminescent Tb_xEu_y(BDC) complexes assembled within liposome-based nanoreactors

Aaron Torres-Huerta, Miriam de J. Velásquez-Hernández, Sven Lempereur, Ludovic Troian-Gautier, Giulia Veronesi, and Hennie Valkenier

SUPPLEMENTAL METHODS

1. Chloride Ion-Selective Electrode (Cl-ISE)

Chloride concentrations outside and inside the liposomes were quantified using a Fisherbrand Accument AB250 chloride ion-selective electrode. The electrode was calibrated prior to each measurement using $NaNO_3$ (0.5 M) solutions containing NaCl at concentrations of 1 ppm, 10 ppm, 100 ppm, and 1000 ppm.
For each measurement, 3 mL of the $LnCl_3$@LP suspension (3 mM lipid) was transferred into a 25 mL beaker and stirred gently. The Cl-ISE was immersed into the sample, and the reading was recorded once it stabilized, corresponding to the external chloride concentration $[Cl^-]_{ext}$. To determine the total chloride content, 100 µL of Triton X-100 (5% w/w in water) was added to lyse the liposomes, and the measurement taken after complete lysis was recorded as the global chloride concentration $[Cl^-]_{global}$. See Figure S1.

2. Scanning Electron Microscopy and Energy-Dispersive X-ray Spectroscopy (SEM-EDX)

SEM-EDX analyses were conducted using an APREO S Thermo Fisher scanning electron microscope equipped with a Trinity detector system along an A-tube biased at 8 kV. For sample preparation, a small drop of the liposome suspension was deposited onto a silicon chip and dried under vacuum. To remove residual Na_2SO_4, the dried samples were gently rinsed with milli-Q water and then dried again under vacuum. To ensure proper surface conductivity during imaging, the samples were coated with a thin carbon layer (<5 nm) using a vacuum sputter coater. EDX spectra were acquired from at least three different vesicles to confirm elemental composition and ensure representativity. See Figure S4.

3. Quantum yield determinations

UV-vis absorption spectra were recorded on an Agilent Cary 60 spectrophotometer in a quartz cuvette with a 1 cm path length.

Photoluminescence measurements for quantum yield determinations were recorded on an Edinburgh Instruments FS5 Spectrofluorometer. The steady-state photoluminescence spectra were recorded using a 150 W Xenon arc lamp as the excitation source. The photoluminescence was detected at a right angle to the excitation beam using a single photon counting PMT-900 with PMT-EXT, allowing detection up to 980 nm in a temperature stabilized housing.

The measurements were performed four times, using water for the complexes and 0.05 M H_2SO_4 for the quinine reference.[2] Emission spectra were recorded under identical instrumental settings for all measurements. The quantum yield was calculated by integrating the emission spectra over the range 360–750 nm, with correction for the absorbance at the excitation wavelength (250 nm), using the following equation:

$$\emptyset_S = \emptyset_R \left(\frac{PL_S}{PL_R}\right)\left(\frac{1-10^{-A_R}}{1-10^{-A_S}}\right)\left(\frac{\eta_S}{\eta_R}\right)^2$$

(S) Sample; (R) Reference = quinine
Ø : Emission Quantum Yield; $\emptyset_R = 0.52$

PL : Photoluminescence intensity
A : Absorbance at the excitation λ
η: Refractive index of the solution solvent η_S = 0.337; η_R= 0.333

4. X-ray Absorption Spectroscopy (XAS)

Experimental Tb L_{III}-edge X ray Absorption Spectroscopy (XAS) data were acquired on the beamline SAMBA at the SOLEIL synchrotron, Saint-Aubin, France.[3] The energy region 7.35-8.20 keV with a Si(220) double-crystal monochromator equipped with sagittal focusing. Drops of solution samples were frozen in liquid nitrogen (LN2) and measured in a He cryostat. The spectra were acquired in fluorescence mode, using a 35-elements monolithic planar Ge pixel array detector.
Liposomal suspensions were concentrated to achieve a final lipid concentration of 23 mM. 5 to 20 spectra were acquired and averaged using the Fastosh software.[4] Further data reduction and analysis was performed with the Larix GUI provided within the Larch data analysis suite.[5] EXAFS spectra were extracted and Fourier-transformed in the k range [2.6, 10] $Å^{-1}$, with a k-weight of 3, then fitted in the real space in the region [1.5 – 3.8] Å. Theoretical amplitudes and phase shifts were generated with the FEFF8.0 code,[6] including self-consistent calculations, using Tb oxychloride (ICSD 120251) as the input structure.[7] Two or three single-scattering paths were necessary to reproduce the experimental spectra, corresponding to atomic shells populated by O, Cl, and Tb around the Tb absorber. For each atomic shell, the free fit parameters were the number of atoms (N), interatomic distances (R), and Debye-Waller factors (σ^2). A common shift in the energy origin (ΔE_0) was assigned to all scattering paths and allowed to vary. See Figure S29 and S30 and Table S1.

5. FT-IR spectroscopy

To further confirm the lanthanide-dicarboxylate coordination, FT-IR spectroscopy was performed. For these measurements, the external sulphate solution was replaced with a 21 mM NaCl solution (pH 7) to avoid interference from SO_4^{2-} absorption bands. Furthermore, a second dialysis step with 21 mM NaCl solution was performed after the Tb_xEu_yBDC@LP formation to remove any remaining exterior carboxylate salts. See Figure S31 and S32.

SUPPLEMENTAL NOTES

NOTE S1. Formation of bilamellar vesicles

The unilamellar-to-bilamellar transition may be attributed to osmotic pressure variations between the bulk solution and the internal liposomal cavity during TbBDC@LP formation. This osmotic shrinkage is likely induced by two key processes: (*i*) the exchange of one BDC^{2-} anions for two Cl^- ions via an antiport mechanism to avoid the buildup of an electrostatic potential and (*ii*) the coordination of the BDC^{2-} with Tb^{3+} cations inside the liposome (Figure S2a). The combination of these processes induces osmotic stress, driving the inward folding of the lipid bilayer and the subsequent formation of bilamellar vesicles (Figure S2b).[1]

NOTE S2. Emission studies in RGB systems

NH_2BDC^{2-} exhibits fluorescence between 400 and 550 nm when excited at 285 nm (Figure S19). We note that the 450 nm long-pass filter (Figure S20), required to suppress excitation scattering and overtones that would be visible at 570 nm, partially attenuated the blue edge of the NH_2BDC^{2-} emission band (Figure S19b). However, the characteristic Tb^{3+} (542 nm) and Eu^{3+} (614 nm) emission bands are hardly affected.

NOTE S3. X-ray Absorption Spectroscopy (XAS)

We investigated the formation of Tb-BDC complex with X-ray Absorption Spectroscopy (XAS). To do so, we measured the Tb L_{III}-edge XAS spectra of three solution samples: the $TbCl_3$ salt, $TbCl_3$ encapsulated in liposomes ($TbCl_3$@LP), and $TbCl_3$ co-encapsulated in liposomes with BDC (TbBDC@LP). The X-ray Absorption Near Edge Structure (XANES) region of the X-ray absorption coefficient of the three samples is reported in Figure S29A. The spectra show minimum variations, indicative of a nearly identical coordination sphere, comprising the nature and number of the first neighbors, and their geometry. This is consistent with a same coordination sphere in all samples, composed by O atoms from water or carboxylates, which is favored in lanthanides.[8]

In contrast, the Fourier Transformed Extended X-ray Absorption Fine Structure (FT-EXAFS, Figure S29B) spectra reveal subtle but significant differences between the three samples. In particular, the spectrum of the $TbCl_3$ salt differs from those of $TbCl_3$@LP and TbBDC@LP, whereas the latter two nearly overlap in the region 1.5 - 4 Å. All samples display a first-shell peak centered at ~ 2 Å (not phase-corrected), the intensity of which is higher in the $TbCl_3$ salt. This observation is consistent with the similarity of XANES spectra, the latter region being more sensitive to the first neighbors of Tb^{3+} than to further atomic shells.
The fit of the FT-EXAFS spectra based on a structural model comprising O first neighbors, as well as Cl and Tb atoms, confirms that in all samples Tb^{3+} binds O atoms at a distance of 2.38-2.39 Å. The quantitative fit results are reported in Table S1. The average number of O atoms is slightly higher in $TbCl_3$ in solution than in $TbCl_3$ encapsulated into liposomes (6.4±0.2 *vs* 5.9±0.2), regardless of the presence of BDC.

In $TbCl_3$ in solution, an average number of 1.3 ± 0.4 Cl atoms surrounds the Tb absorber, with a Tb-Cl distance of 3.25 Å. This distance is longer than one observed in the literature for a $TbCl_3{\cdot}6H_2O$ solid powder (reported in Table S1 for comparison), consistently with the differences expected in solution *vs* solid samples. Accordingly, a higher disorder (indicated by an increase in the dynamical parameter σ^2, see Table S1) is observed in the $TbCl_3$ solution

with respect to the $TbCl_3 \cdot 6H_2O$ powder. When $TbCl_3$ is encapsulated into liposomes, the EXAFS analysis exposes the formation of a Tb cluster, indicated by the presence of 6.0 ± 1.7 Tb atoms surrounding the Tb absorber, with an average Tb-Tb distance of 3.34 ± 0.02 Å (Table S1). When $TbCl_3$ and BDO are co-encapsulated, these values are unchanged within the error, as well as the number of O neighbors and the Tb-O distance.
It is worth to mention that both Cl and Tb neighbors are necessary to fit the features in the 2.8-3.8 Å region of the FT-EXAFS spectra in the $TbCl_3$@LP and TbBDC@LP samples (Figure S29B). The interplay between these two contributions is particularly clear in the real part of the FT EXAFS signal (Figure S30, dark yellow *vs* magenta curves), where they cancel out in the range 2.0-3.0 Å, and they sum up to account for the spectral features in the 3.0-3.8 Å region.

Overall, these results indicate that the confinement into liposomes fosters the formation of Tb clusters, in which Tb^{3+} binds oxygen atoms from the solution of from the phosphate heads of the membrane. When BDC is co-encapsulated, the carboxylate moieties can replace O atoms in the pre-formed Tb-cluster, inducing the formation of the Tb-BDC complex and the acquisition of luminescence, without changing the nature of the Tb^{3+} ligands.

NOTE S4. FT-IR spectroscopy

Six representative samples were analysed: $Tb_{100}Eu_{00}$BDC@LP, $Tb_{00}Eu_{100}$BDC@LP, $Tb_{50}Eu_{50}$BDC@LP, $Tb_{100}Eu_{00}NH_2$BDC@LP, $Tb_{00}Eu_{100}NH_2$DC@LP and $Tb_{50}Eu_{50}NH_2$BDC@LP. These spectra were compared with those obtained from control samples, including $LnCl_3$@LP and $LnCl_3$@LP + **T1**, as well as the corresponding free dicarboxylate ligands (BDC^{2-} or NH_2BDC^{2-}). In dried samples of BDC-based systems ($Tb_{100}Eu_{00}$BDC@LP, $Tb_{00}Eu_{100}$BDC@LP, and $Tb_{50}Eu_{50}$BDC@LP), the asymmetric $RCOO^-$ stretching vibration appeared at 1562 cm^{-1}, which is shifted compared to the uncoordinated BDC^{2-} ligand (1551 cm^{-1}). Similarly, for NH_2BDC-based systems ($Tb_{100}Eu_{00}NH_2$BDC@LP, $Tb_{00}Eu_{100}NH_2$DC@LP, and $Tb_{50}Eu_{50}NH_2$BDC@LP) the asymmetric $RCOO^-$ band was observed in the range 1566-1570 cm^{-1}, compared to 1557 cm^{-1} for the free NH_2BDC^{2-} ligand.

SUPPLEMENTAL FIGURES

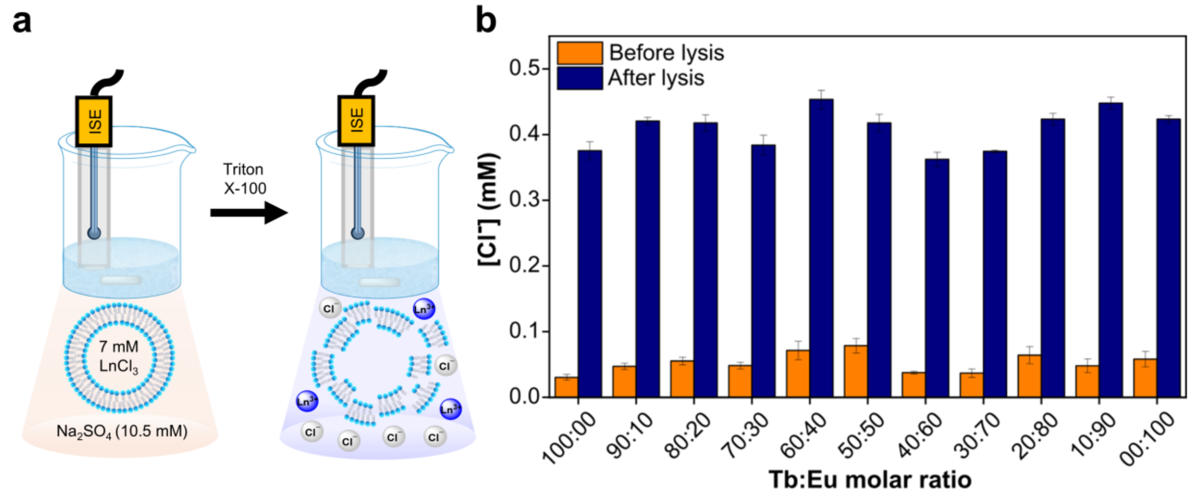

Figure S1: Quantification of encapsulated lanthanide chloride content. A) Schematic of Cl-ISE assay to determine Cl^- concentration before and after lysing $Tb_xEu_yCl_3$@LP systems. B) Chloride ion-selective electrode (Cl-ISE) measurements of $Tb_xEu_yCl_3$@LP suspensions. Error bars represent the standard deviation of three independent measurements.

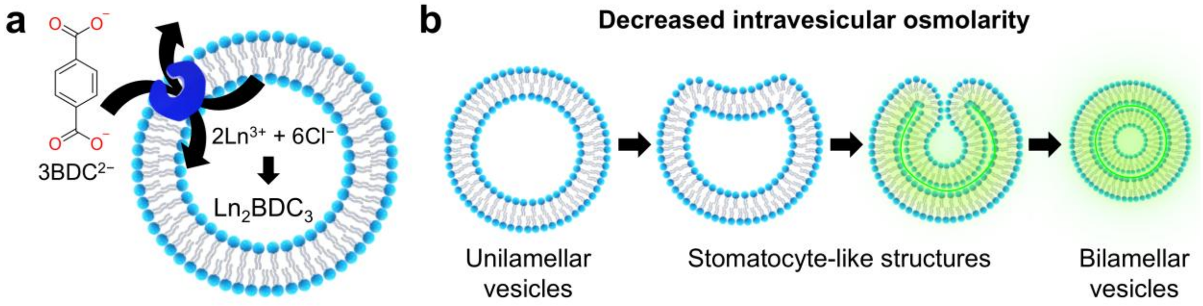

Figure S2: Schematic illustration of bilamellar vesicle formation. A), B) Schematic representation of bilamellar vesicle formation induced by osmolarity reduction upon the anti-port BDC^{2-} transport.

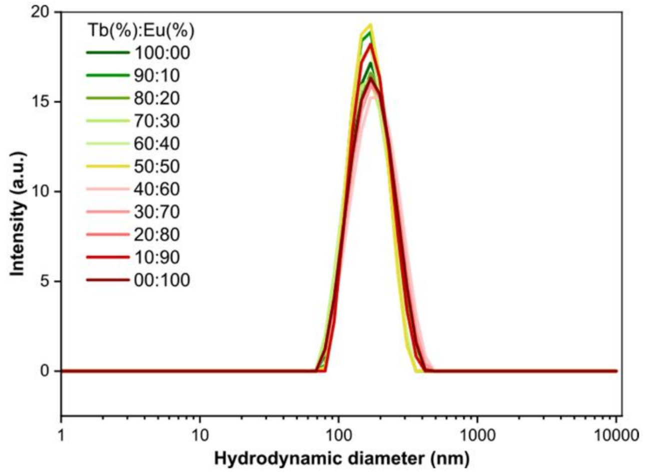

Figure S3: Full-range DLS intensity. Distributions for representative Tb_xEu_yBDC@LP systems.

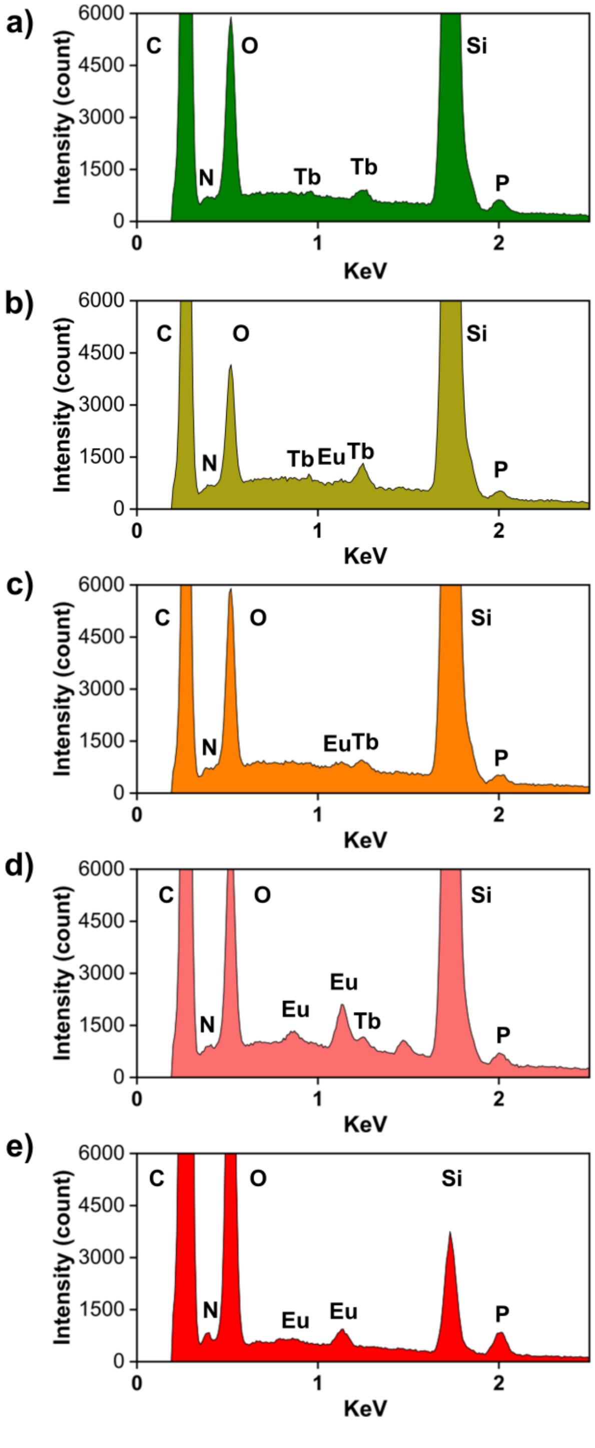

Figure S4: Energy-dispersive X-ray spectroscopy (EDX) elemental analysis. EDX elemental analysis of selected Tb_xEu_yBDC@LP samples: A) $Tb_{100}Eu_{00}$BDC@LP, B) $Tb_{80}Eu_{20}$BDC@LP, C) $Tb_{50}Eu_{50}$BDC@LP, D) $Tb_{20}Eu_{80}$BDC@LP, and E) $Tb_{00}Eu_{100}$BDC@LP.

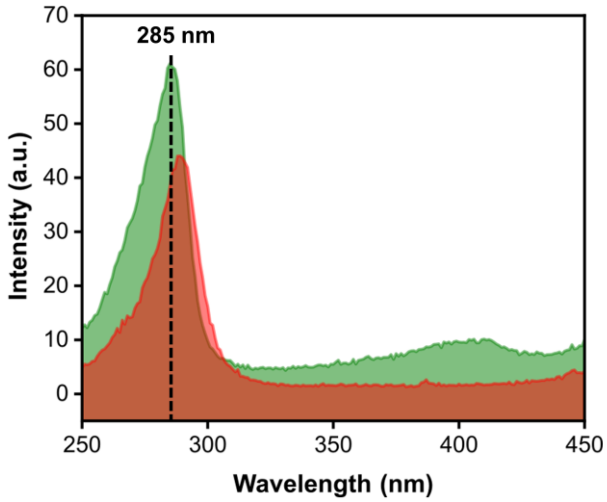

Figure S5: Excitation spectra in Tb and Eu reference systems. Excitation spectra of $Tb_{100}Eu_{00}$BDC@LP (λ_{em} = 542) and $Tb_{00}Eu_{100}$BDC@LP (λ_{em} = 614).

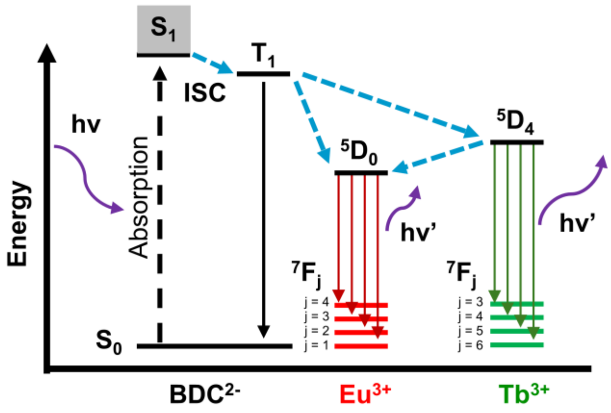

Figure S6: Schematic representation of the energy transfer pathways in Tb_xEu_yBDC@LP systems. Excitation of the BDC^{2-} ligand results in energy transfer to the $^5D_4$ excited state of Tb^{3+} and the $^5D_0$ excited state of Eu^{3+}. In mixed-metal systems, additional intermetallic energy transfer occurs from Tb^{3+} to Eu^{3+}.

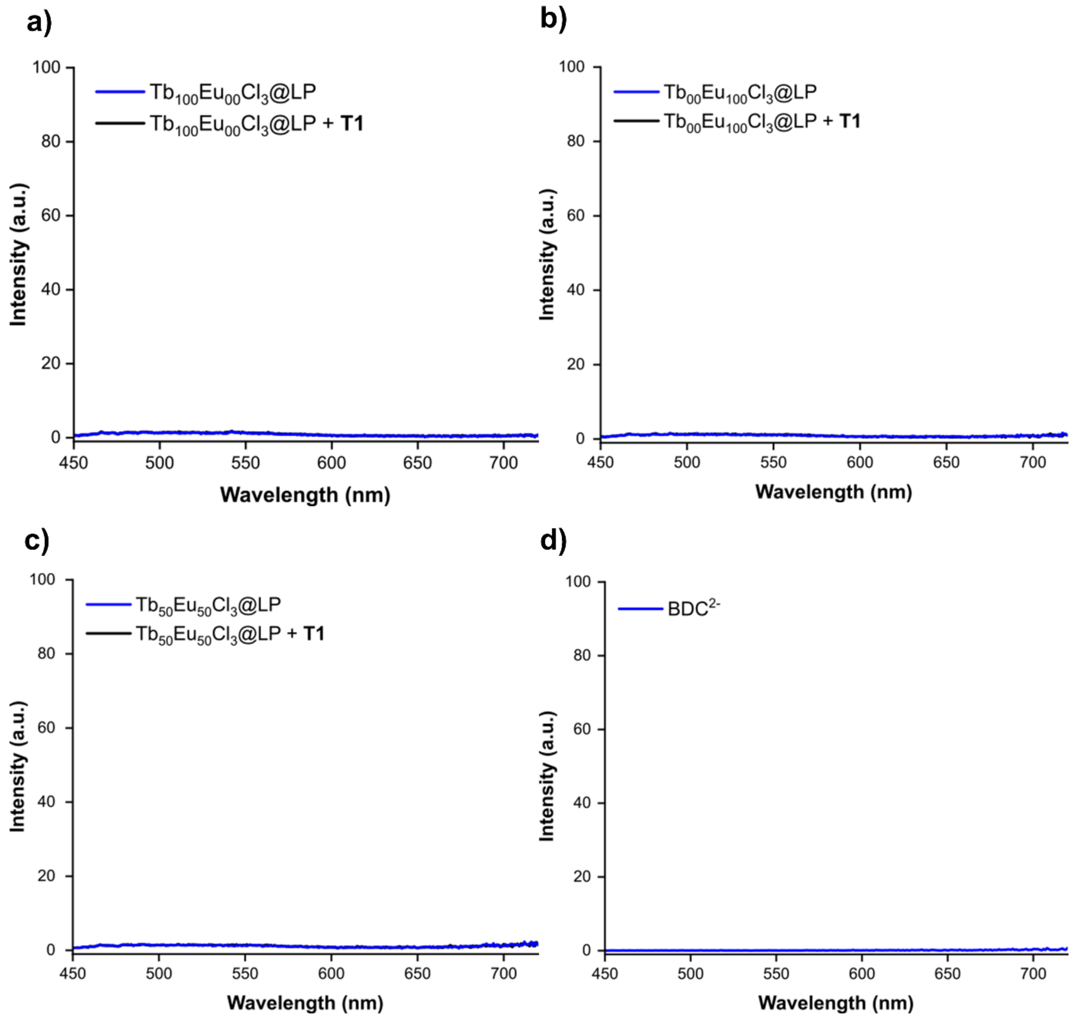

Figure S7: Control emission spectra. Emission spectra recorded under 285 nm excitation (using a 450 nm long-pass filter) for 3 mL liposome samples in the absence and presence of the anion transporter **T1**: A) $Tb_{100}Eu_{00}Cl_3$@LP and $Tb_{100}Eu_{00}Cl_3$@LP + **T1**; B) $Tb_{00}Eu_{100}Cl_3$@LP and $Tb_{00}Eu_{100}Cl_3$@LP + **T1**; C) $Tb_{50}Eu_{50}Cl_3$@LP and $Tb_{50}Eu_{50}Cl_3$@LP + **T1**; and D) 1 mM BDC^{2-} in 3 mL of sulphate solution.

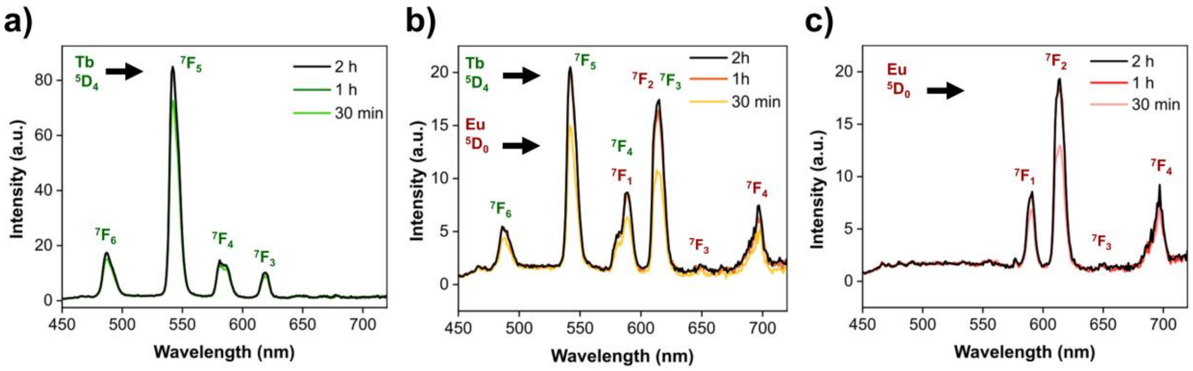

Figure S8: Stability of Tb_xEu_yBDC@LP systems over time. Emission spectra recorded at 30 minutes, 1 hour and 2 hours for selected Tb_xEu_yBDC@LP samples: A) $Tb_{100}Eu_{00}$BDC@LP, B) $Tb_{50}Eu_{50}$BDC@LP, and C) $Tb_{00}Eu_{100}$BDC@LP (λ_{ex}= 285 nm, using a 450 nm long-pass filter). All samples exhibit negligible changes in emission intensity after 1 hour.

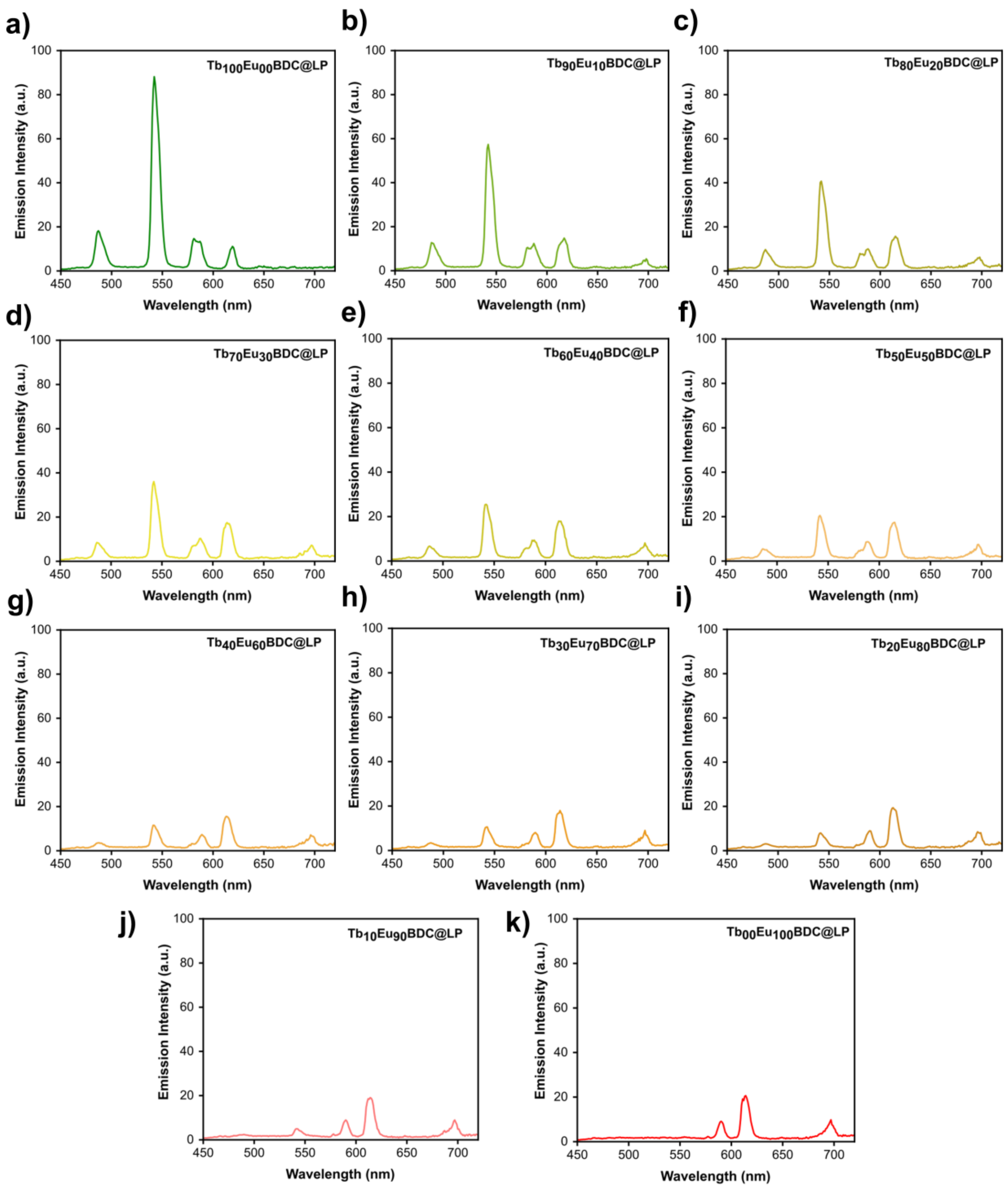

Figure S9: Emission spectra of Tb_xEu_yBDC@LP samples with varying Tb:Eu molar ratios. The spectra were recorded after complexation, and no further changes in emission intensity were observed upon excitation at 285 nm, using a 450nm long-pass filter.

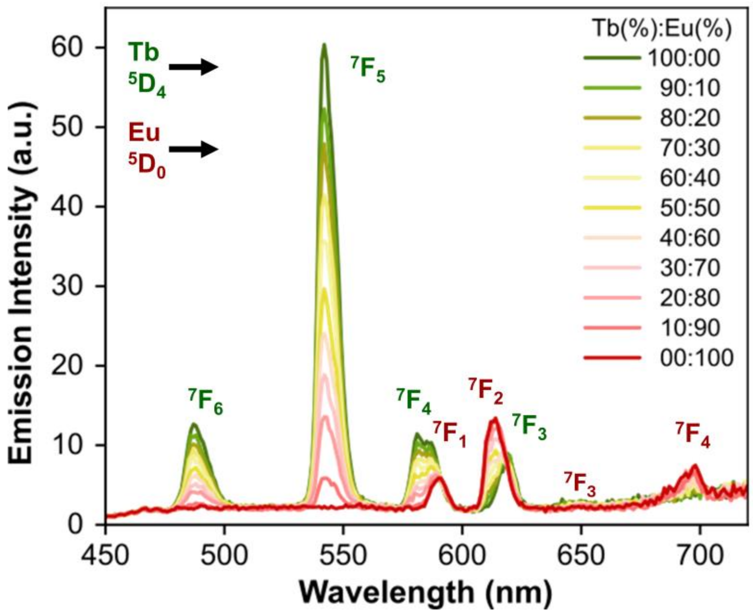

Figure S10: Emission spectra of physically mixed lanthanide systems without intermetallic interaction. Emission spectra for physically mixed samples composed of $Tb_{100}Eu_{00}BDC@LP$ and $Tb_{00}Eu_{100}BDC@LP$ in varying Tb:Eu molar ratios (λ_{ex}= 285 nm, using a 450nm long-pass filter).

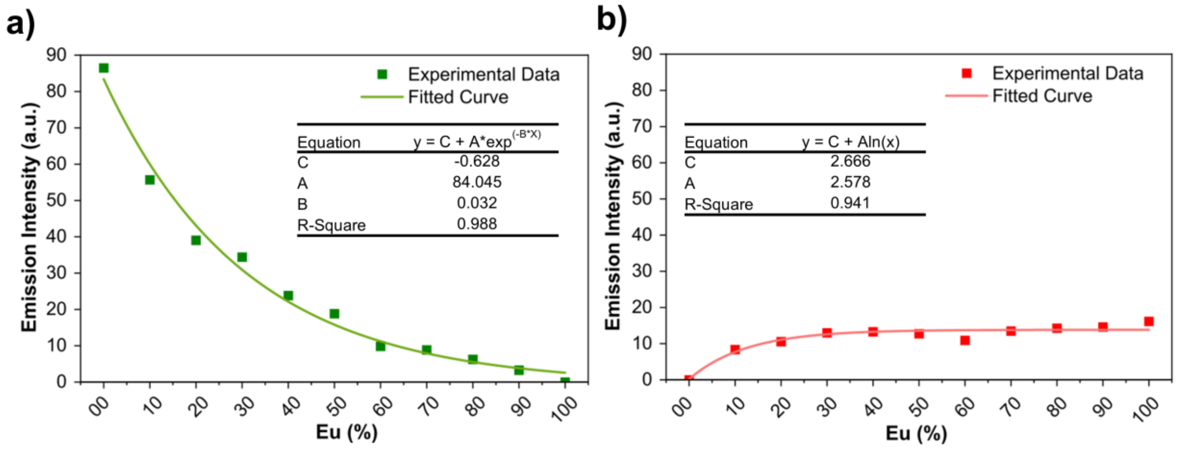

Figure S11: Non-linear variation of Tb and Eu emission intensities in multivariate systems. Fitting of the emission intensities at 542 nm and 614 nm as a function of Eu^{3+} mole fraction in $Tb_xEu_yBDC@LP$ samples.

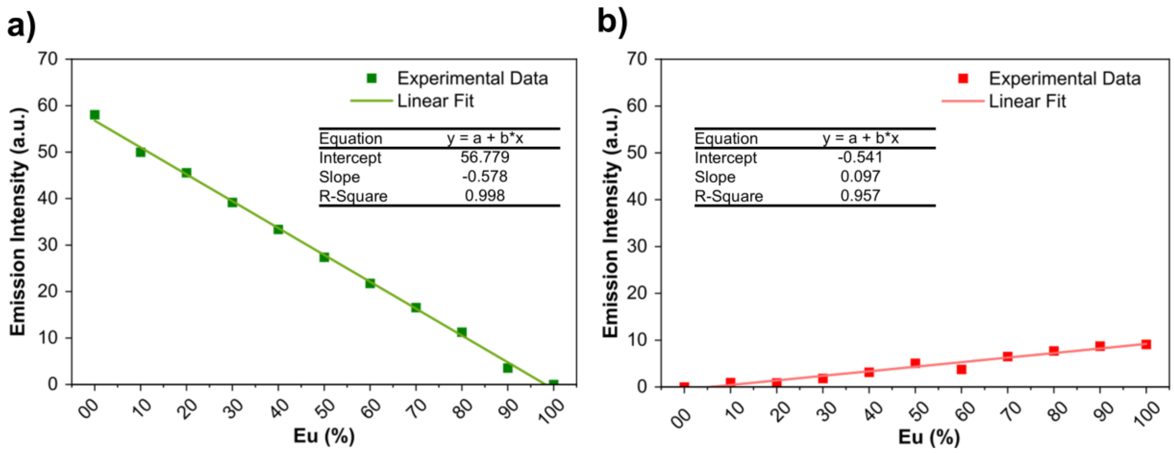

Figure S12: Linear variation of Tb and Eu emission intensities in physical mixtures. Linear fitting of the emission intensities at 542 nm and 614 nm as a function of Eu^{3+} mole fraction in physically mixed samples composed of $Tb_{100}Eu_{00}$BDC@LP and $Tb_{00}Eu_{100}$BDC@LP. The linear trends confirm the independent luminescence contributions of physically separated lanthanide complexes, in contrast to the cooperative behaviour observed in co-encapsulated systems.

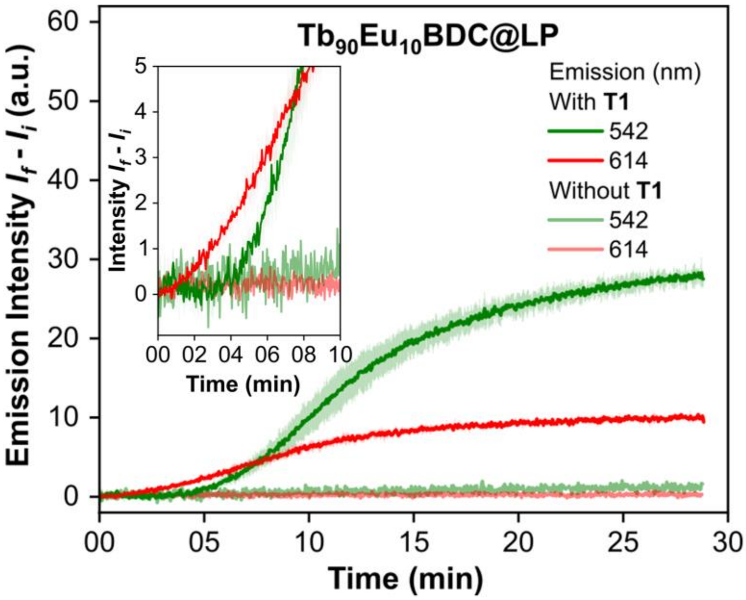

Figure S13: Kinetic emission data for $Tb_{90}Eu_{10}$BDC@LP. Emission intensities at 542 nm (green line) correspond to the Tb^{3+} $^5D_4 \rightarrow ^7F_5$ transition, while those at 614 nm (red line) correspond to the Eu^{3+} $^5D_0 \rightarrow ^7F_2$ transition. Control experiments without transporter **T1** are shown in lighter green and red lines. Shaded areas represent the standard deviation of three independent measurements.

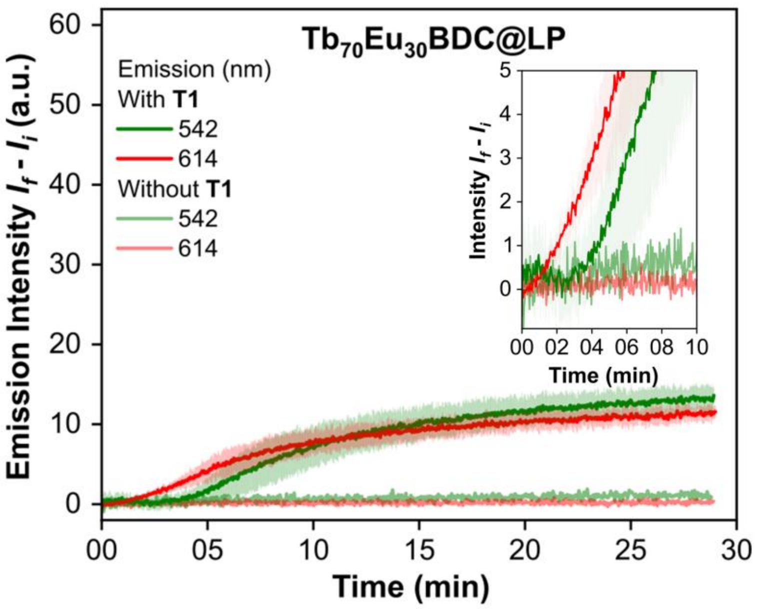

Figure S14: Kinetic emission data for $Tb_{70}Eu_{30}$BDC@LP. Emission intensities at 542 nm (green line) correspond to the Tb^{3+} $^5D_4 \rightarrow ^7F_5$ transition, while those at 614 nm (red line) correspond to the Eu^{3+} $^5D_0 \rightarrow ^7F_2$ transition. Control experiments without transporter **T1** are shown in lighter green and red lines. Shaded areas represent the standard deviation of three independent measurements.

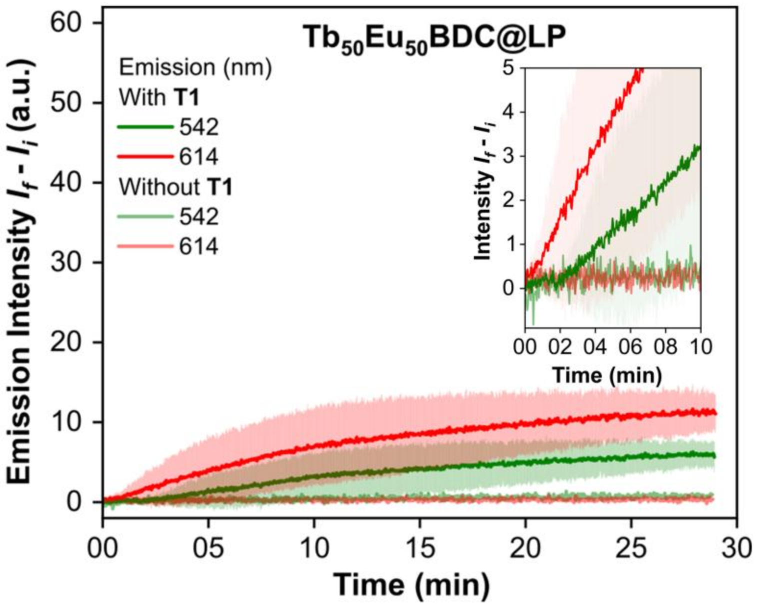

Figure S15: Kinetic emission data for $Tb_{50}Eu_{50}$BDC@LP. Emission intensities at 542 nm (green line) correspond to the Tb^{3+} $^5D_4 \rightarrow ^7F_5$ transition, while those at 614 nm (red line) correspond to the Eu^{3+} $^5D_0 \rightarrow ^7F_2$ transition. Control experiments without transporter **T1** are shown in lighter green and red lines. Shaded areas represent the standard deviation of three independent measurements.

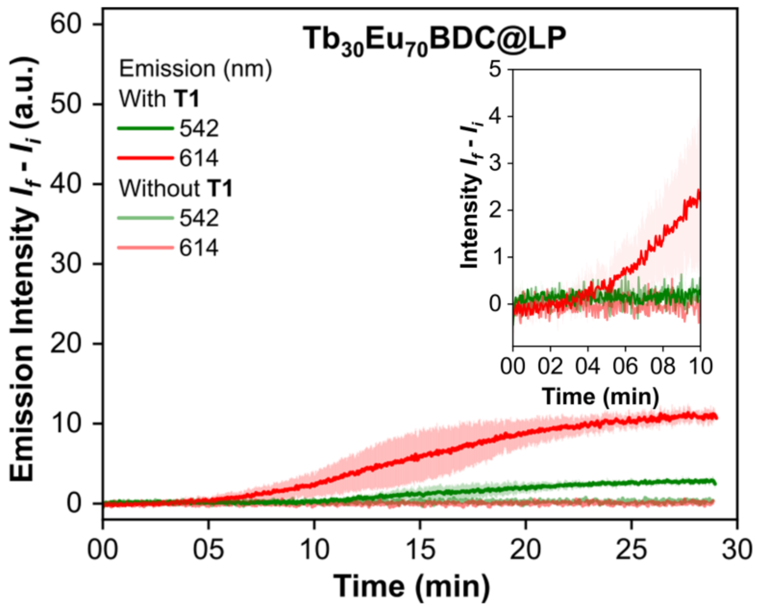

Figure S16: Kinetic emission data for $Tb_{30}Eu_{70}$BDC@LP. Emission intensities at 542 nm (green line) correspond to the Tb^{3+} $^5D_4 \rightarrow ^7F_5$ transition, while those at 614 nm (red line) correspond to the Eu^{3+} $^5D_0 \rightarrow ^7F_2$ transition. Control experiments without transporter **T1** are shown in lighter green and red lines. Shaded areas represent the standard deviation of three independent measurements.

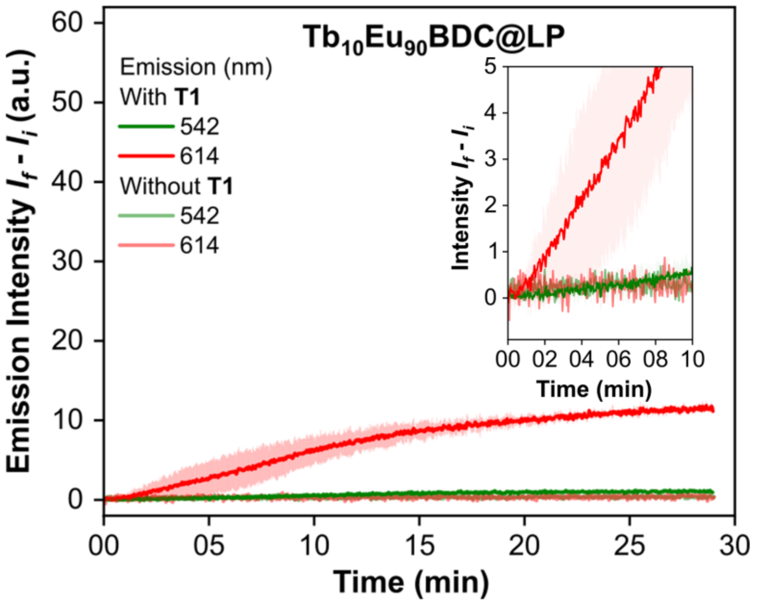

Figure S17: Kinetic emission data for $Tb_{10}Eu_{90}$BDC@LP. Emission intensities at 542 nm (green line) correspond to the Tb^{3+} $^5D_4 \rightarrow ^7F_5$ transition, while those at 614 nm (red line) correspond to the Eu^{3+} $^5D_0 \rightarrow ^7F_2$ transition. Control experiments without transporter **T1** are shown in lighter green and red lines. Shaded areas represent the standard deviation of three independent measurements.

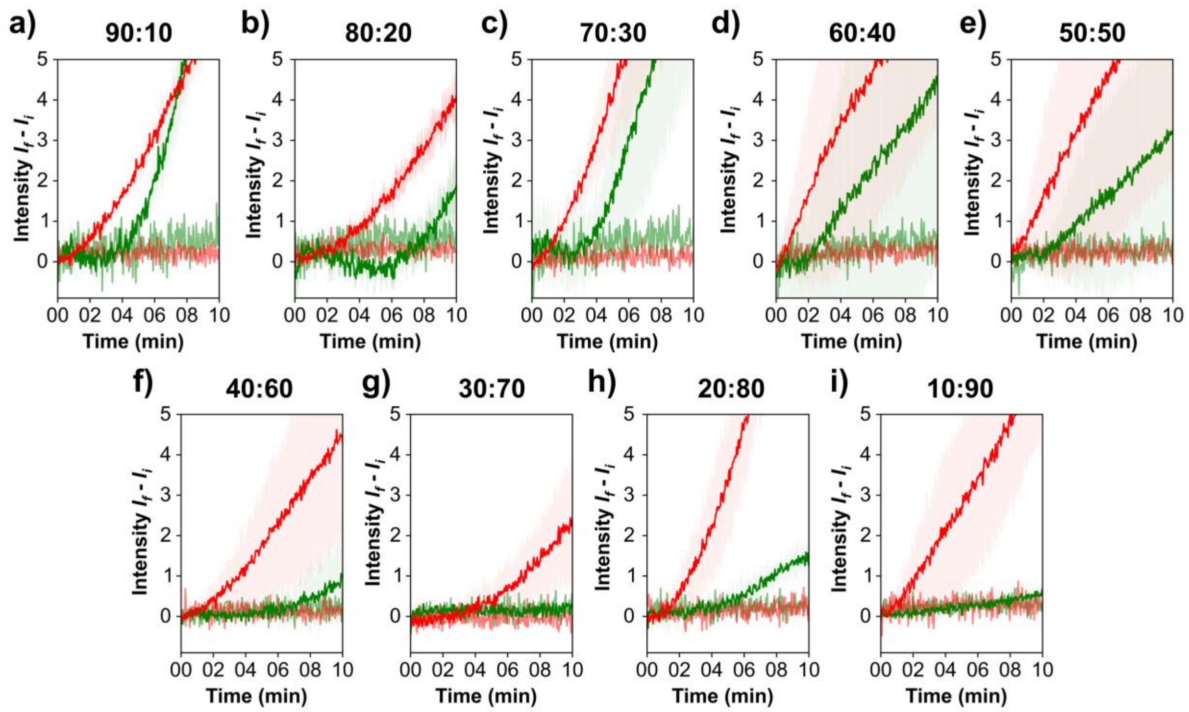

Figure S18: Real-time emission kinetics during early-stage coordination. Real-time emission monitoring of the coordination between BDC and lanthanide ions within liposome-based nanoreactors during the first 10 minutes following reactant addition. Emission intensities at 542 nm (green line) correspond to the Tb^{3+} $^5D_4 \rightarrow ^7F_5$ transition, while those at 614 nm (red line) correspond to the Eu^{3+} $^5D_0 \rightarrow ^7F_2$ transition. Kinetic data are shown for: A) $Tb_{90}Eu_{10}$BDC@LP, B) $Tb_{80}Eu_{20}$BDC@LP, C) $Tb_{70}Eu_{30}$BDC@LP, D) $Tb_{60}Eu_{40}$BDC@LP, E) $Tb_{50}Eu_{50}$BDC@LP, F) $Tb_{40}Eu_{60}$BDC@LP, G) $Tb_{30}Eu_{70}$BDC@LP, H) $Tb_{20}Eu_{80}$BDC@LP, and I) $Tb_{10}Eu_{90}$BDC@LP. Control experiments without transporter **T1** are represented with a slight green and red colour. Shaded areas represent the standard deviation of three independent measurements.

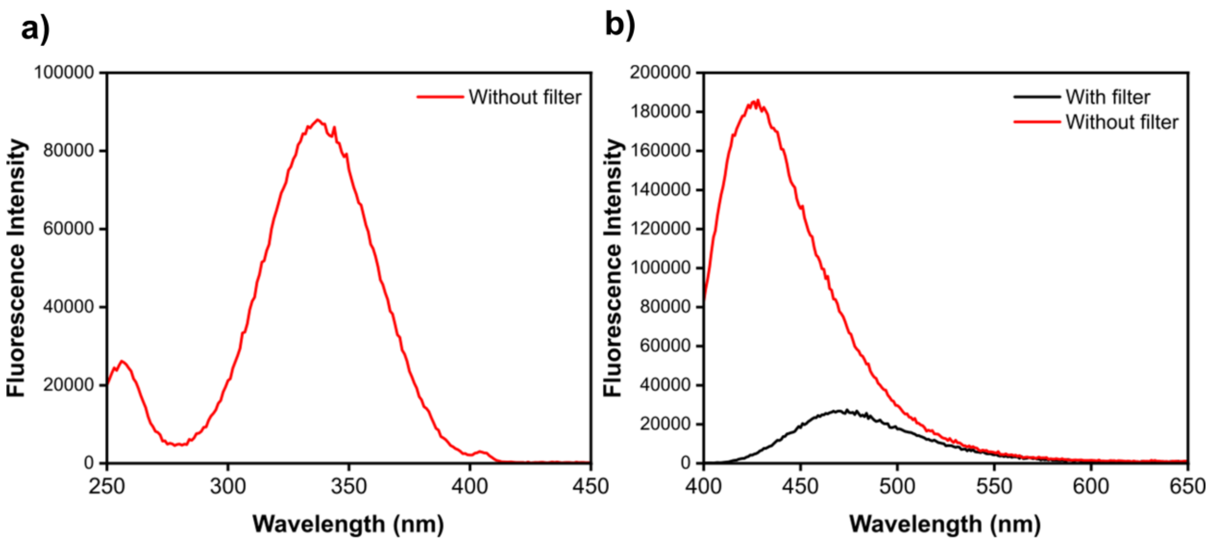

Figure S19. Long-pass filter effect on excitation and emission spectra of NH_2BDC^{2-} ligand. Fluorescence spectra of NH_2BDC^{2-} ligand (1.6 μM in 3 mL of 10.5 mM Na_2SO_4) obtained in the presence and absence of the 450 nm long-pass filter. A) The excitation spectrum was obtained when recording the emission at 470 nm, and B) the emission spectra upon excitation at 285 nm.

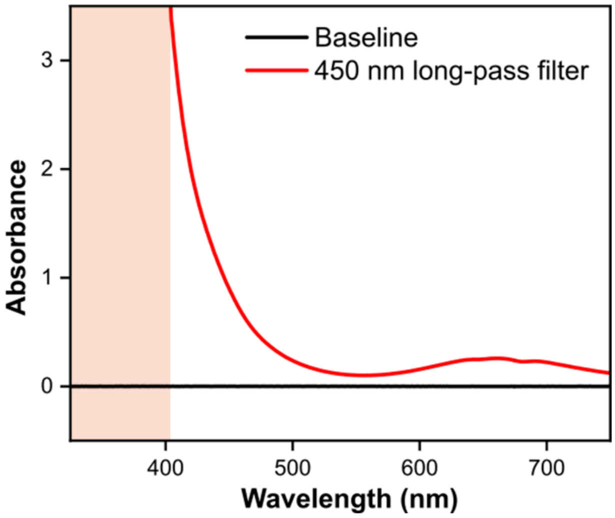

Figure S20: Absorbance profile of the 450 nm long-pass filter used in emission measurements. Spectrum of the 450 nm long-pass filter from 400-750 nm upon excitation at 285 nm.

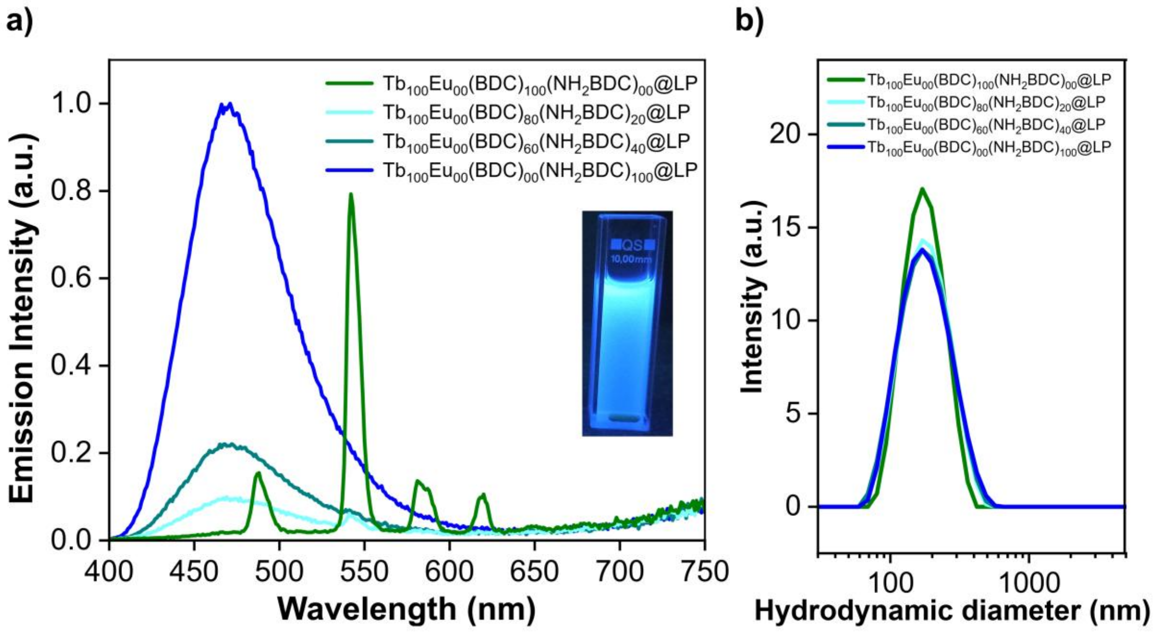

Figure S21: Mixed-ligand systems. A) Emission spectra of mixed-ligand systems prepared with varying BDC^{2-}/NH_2BDC^{2-} molar ratios, showing the evolution from Tb^{3+} emission (λ_{ex}= 285 nm, using a 450nm long-pass filter). Inset: Photograph of the $Tb_{100}Eu_{00}(BDC)_{00}(NH_2BDC)_{100}$@LP sample under 254 nm UV illumination. B) corresponding DLS analysis.

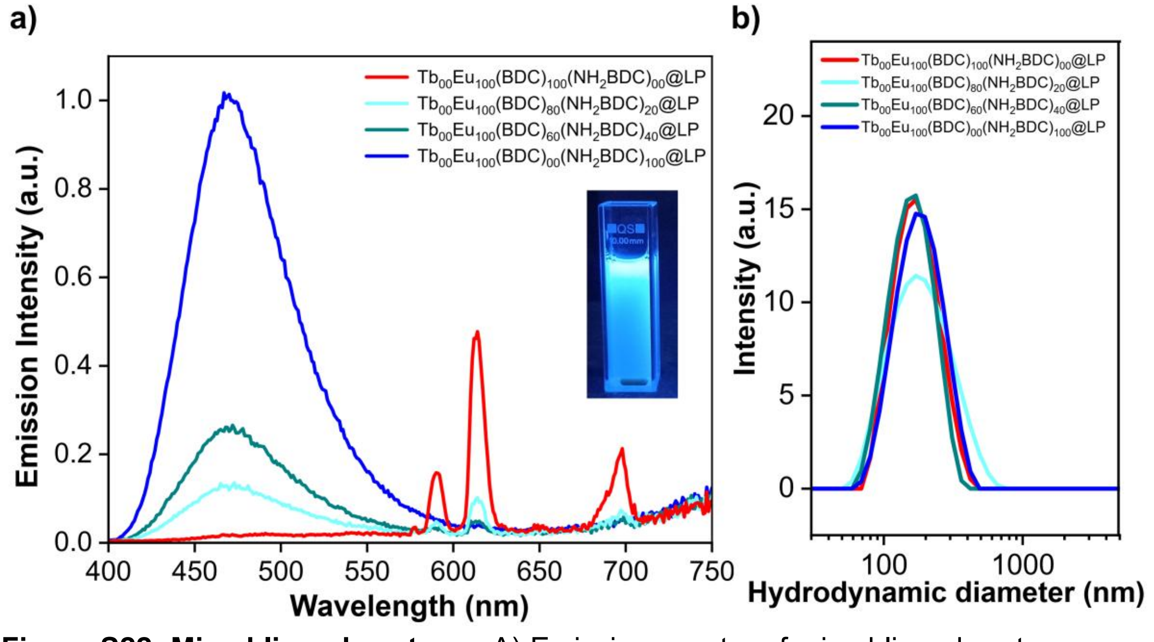

Figure S22: Mixed-ligand systems. A) Emission spectra of mixed-ligand systems prepared with varying BDC^{2-}/NH_2BDC^{2-} molar ratios, showing the evolution from Eu^{3+} emission (λ_{ex}= 285 nm, using a 450nm long-pass filter). Inset: Photograph of the $Tb_{00}Eu_{100}(BDC)_{00}(NH_2BDC)_{100}$@LP sample under 254 nm UV illumination. B) corresponding DLS analysis.

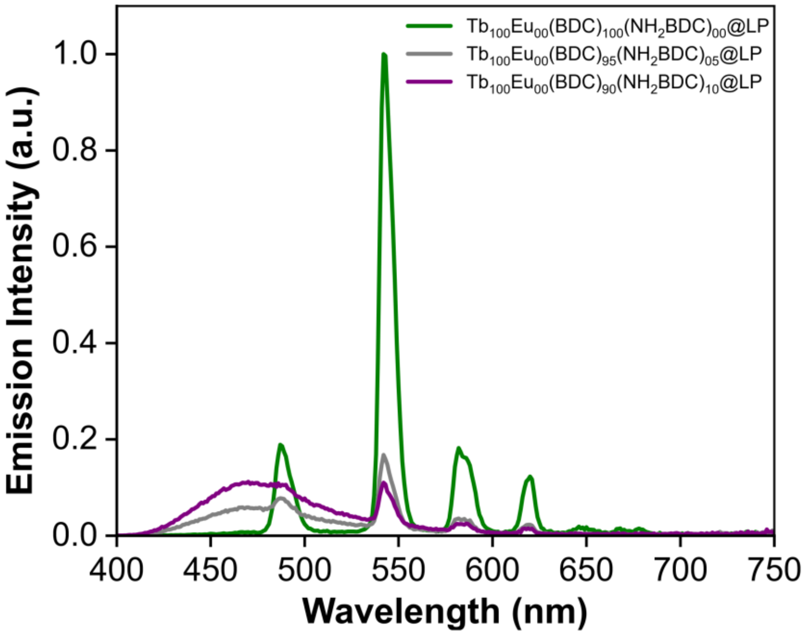

Figure S23: Progressive suppression of Tb emission in mixed-ligand systems. Emission spectra of mixed-ligand systems prepared with BDC^{2-}/NH_2BDC^{2-} molar ratios of 100/00, 95/05, and 90/10, showing the evolution from Tb^{3+} emission (λ_{ex}= 285 nm, using a 450nm long-pass filter).

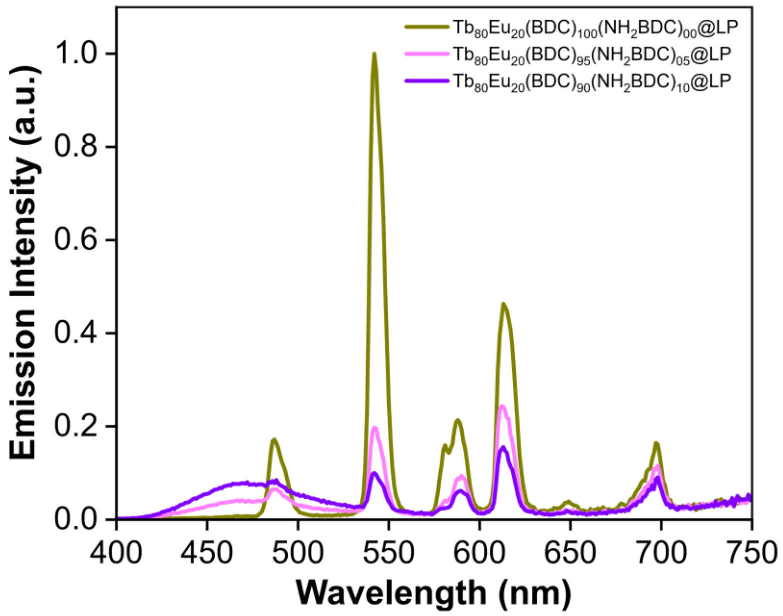

Figure S24: Evolution of Tb and Eu emission in mixed-ligand systems. Emission spectra of mixed-ligand systems prepared with BDC^{2-}/NH_2BDC^{2-} molar ratios of 100/00, 95/05, and 90/10, showing the evolution from Tb^{3+} and Eu^{3+} emissions (λ_{ex}= 285 nm, using a 450nm long-pass filter).

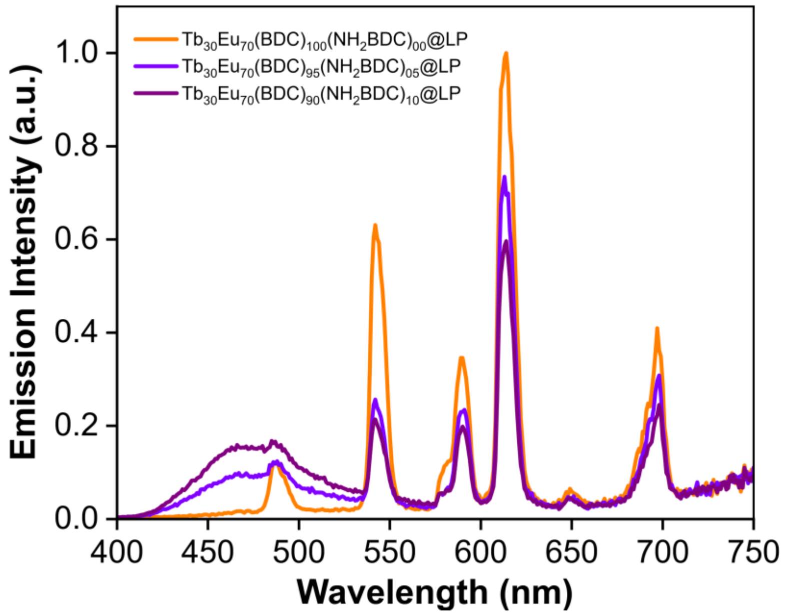

Figure S25: Evolution of Tb and Eu emission in mixed-ligand systems. Emission spectra of mixed-ligand systems prepared with $BDC^{2–}/NH_2BDC^{2–}$ molar ratios of 100/00, 95/05, and 90/10, showing the evolution from Tb^{3+} and Eu^{3+} emissions (λ_{ex}= 285 nm, using a 450nm long-pass filter).

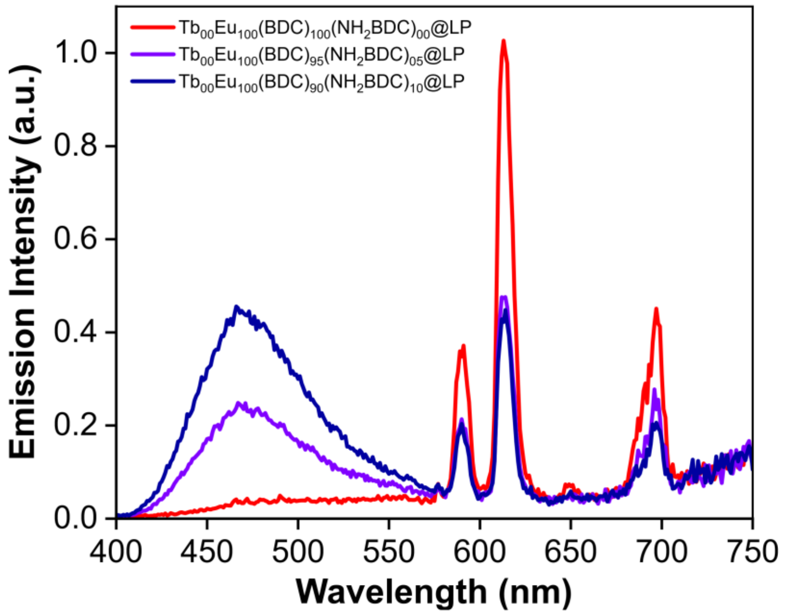

Figure S26: Progressive suppression of Eu emission in mixed-ligand systems. Emission spectra of mixed-ligand systems prepared with $BDC^{2–}/NH_2BDC^{2–}$ molar ratios of 100/00, 95/05, and 90/10, showing the evolution from Eu^{3+} emission (λ_{ex}= 285 nm, using a 450nm long-pass filter).

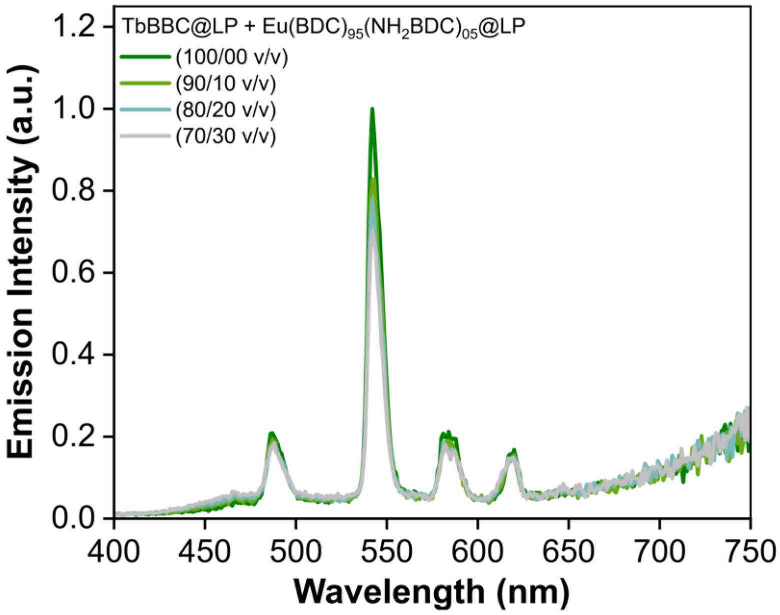

Figure S27: Emission spectra of physically mixed systems. Emission spectra of physical mixtures of $Tb_{100}Eu_{00}$BDC@LP and $Tb_{00}Eu_{100}(BDC)_{95}(NH_2BDC)_{05}$@LP prepared at v/v ratios of 100/00, 90/10, 80/20, and 70/30 (λ_{ex}= 285 nm, using a 450nm long-pass filter).

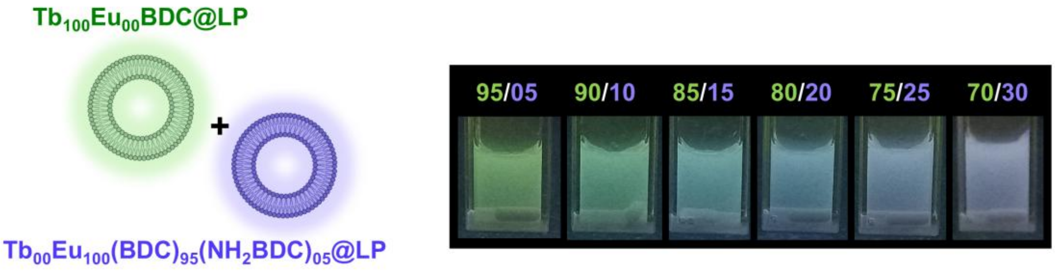

Figure S28: Colour tuning via physical mixing. Colour-tuning strategy based on physical mixtures of green-emissive $Tb_{100}Eu_{00}$BDC@LP and violet-emissive $Tb_{00}Eu_{100}(BDC)_{95}(NH_2BDC)_{05}$@LP, providing a route to cyan and near-white emission.

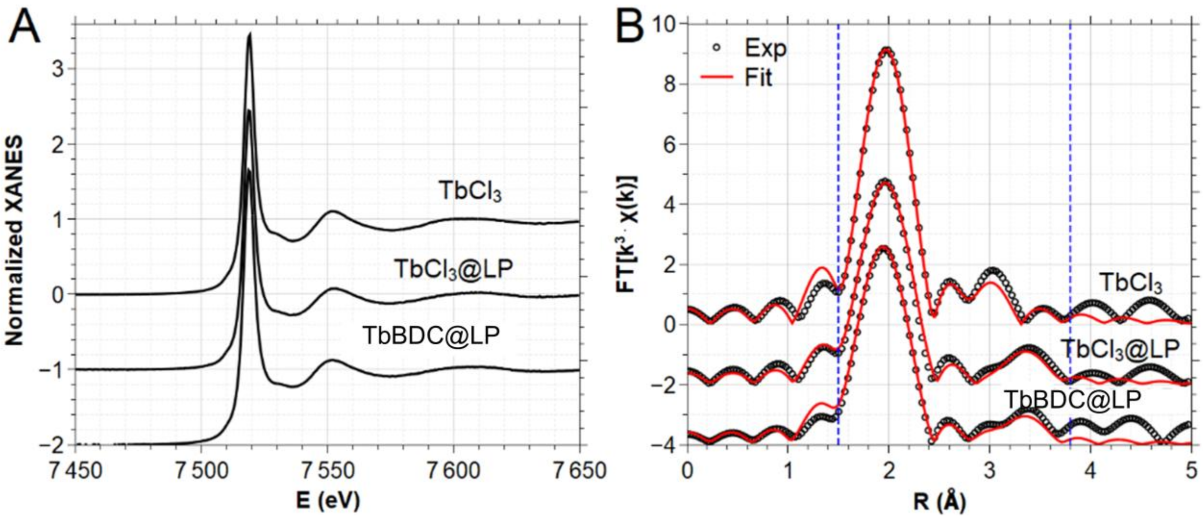

Figure S29: XANES and EXAFS analysis of the coordination environment of Tb in liposome nanoreactors. A) Experimental Tb L_{III}-edge XANES spectra of the $TbCl_3$ salt in solution (top), encapsulated in liposomes (middle), and co-encapsulated with BDC in liposomes (bottom). B) Experimental Fourier-Transformed EXAFS signal (open circles) of the same samples as in panel A, and theoretical curves (red solid) providing the best fit to the experimental data. Blue dashed vertical lines indicate the fitting region.

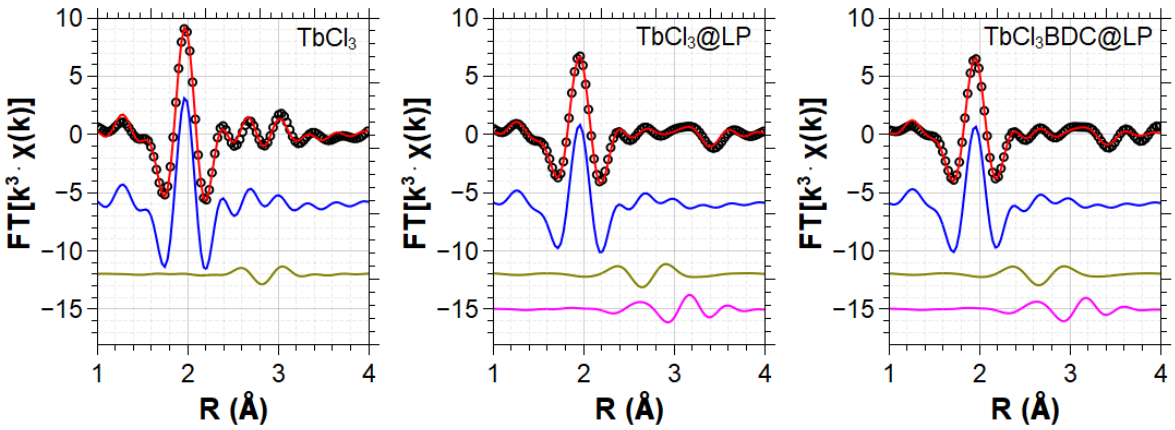

Figure S30: EXAFS fitting analysis. Real part of the Fourier-Transformed Tb L_{III}-edge EXAFS signal. Experimental spectra (open circles) and best-fitting curves (red) based on a theoretical model including Tb-O (blue), Tb-Cl (dark yellow), and Tb-Tb (magenta) contributions. The same model was applied to the three samples: $TbCl_3$ salt in solution (left), encapsulated in liposomes (middle), and co-encapsulated with BDC in liposomes (right).

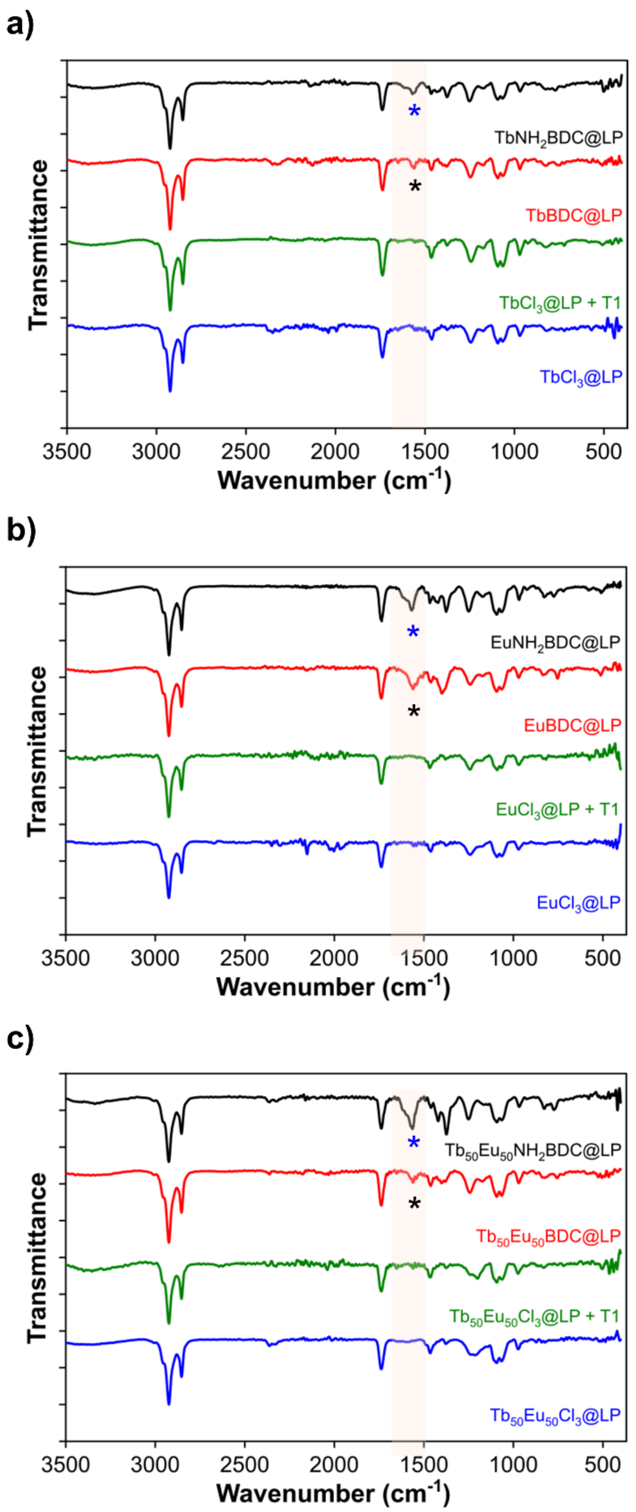

Figure S31: FT-IR analysis. FT-IR spectra comparing lanthanide-chloride and lanthanide-dicarboxylate systems encapsulated within liposomes: A) $TbCl_3$@LP, $TbCl_3$@LP + **T1**, TbBDC@LP, and $TbNH_2BDC$@LP; B) $EuCl_3$@LP, $EuCl_3$@LP + **T1**, EuBDC@LP, and $EuNH_2BDC$@LP; C) $Tb_{50}Eu_{50}Cl_3$@LP, $Tb_{50}Eu_{50}Cl_3$@LP + **T1**, $Tb_{50}Eu_{50}BDC$@LP, and $Tb_{50}Eu_{50}NH_2BDC$@LP. Blue and black stars indicate the asymmetric carboxylate stretching vibration ($\nu_{as}COO^-$) for NH_2BDC^{2-} and BDC^{2-}, respectively.

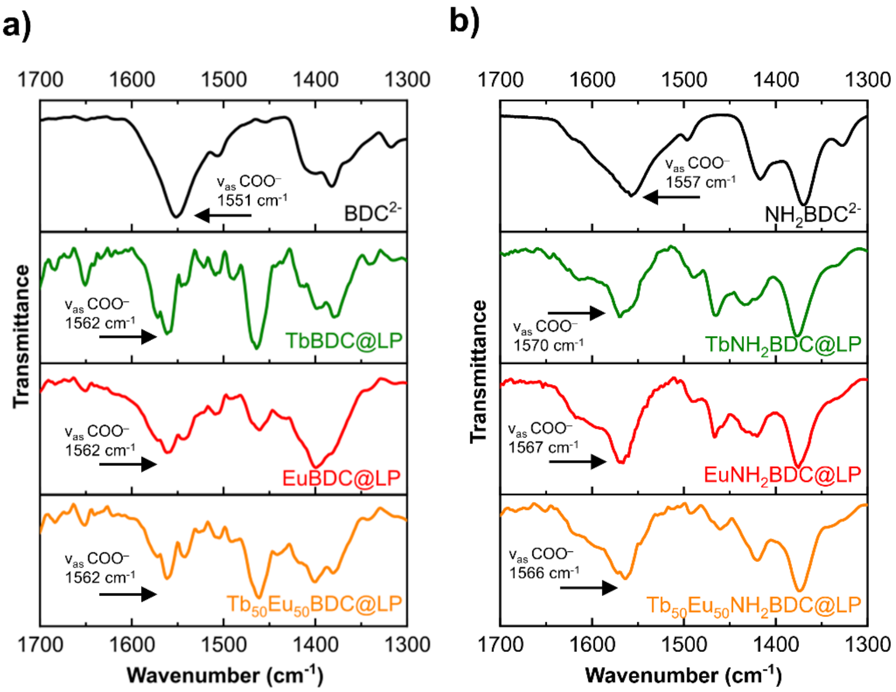

Figure S32: Shifts in asymmetric carboxylate stretching bands in FT-IR spectra. FT-IR spectra comparing the asymmetric carboxylate stretching vibration ($\nu_{as}COO^-$) of: a) TbBDC@LP, EuBDC@LP, $Tb_{50}Eu_{50}$BDC@LP, and the free BDC^{2-} ligand; and b) $TbNH_2$BDC@LP, $EuNH_2$BDC@LP, $Tb_{50}Eu_{50}NH_2$BDC@LP, and the free NH_2BDC^{2-} ligand.

SUPPLEMENTAL TABLE

Table S1: EXAFS-derived structural parameters. Structural and dynamical parameters of the average Tb binding site during the formation of TbMOFs in liposomes, extracted from fitting of Tb L_{III}-edge EXAFS data based on a theoretical model. The values of Tb-O, Tb-Cl, and Tb-Tb distances found in the literature for Tb^{3+} compounds are reported for comparison.[9]

Sample	Tb-O			Tb-Cl			Tb-Tb			ΔE_0 (eV)	R_{fit} (%)
	**N**	R (Å)	σ^2 (10^{-3} Å^2)	**N**	R (Å)	σ^2 (10^{-3} Å^2)	**N**	R (Å)	σ^2 (10^{-3} Å^2)		
**$TbCl_3$**	**6.4 ± 0.2**	2.397 ± 0.005	3.0 ± 0.4	**1.3 ± 0.4**	3.25 ± 0.03	10 ± 4	-	-	-	6.5 ± 0.7	0.65
**$TbCl_3$@LP**	**5.9 ± 0.2**	2.386 ± 0.007	6.1 ± 0.6	**3.5 ± 1.0**	3.10 ± 0.03	23 ± 5	**6.0 ± 1.7**	3.35 ± 0.02	20 ± 4	6.9 ± 0.6	0.75
**TbBCD@LP**	**5.8 ± 0.2**	2.376 ± 0.005	6.0 ± 0.4	**4.1 ± 1.0**	3.10 ± 0.02	27 ± 4	**6.7 ± 1.5**	3.34 ± 0.02	22 ± 2	5.8 ± 0.6	0.33
TbOCl[8]	4	2.24 ± 0.02	7 ± 1	5	3.07 ± 0.03	18 ± 3	4	3.55 ± 0.03	5 ± 2	4 ± 3	1.9
$TbCl_3{\cdot}6H_2O$[8]	6	2.38 ± 0.02	9 ± 1	2	2.73 ± 0.03	10 ± 3	-	-	-	4.2 ± 1.5	0.7

R_{fit} is the goodness of fit calculated as $\Sigma(\chi_{exp}-\chi_{fit})^2/\Sigma(\chi_{exp})^2$, where χ_{exp} is the experimental data point and χ_{fit} the corresponding point in the best-fitting curve.

SUPPLEMENTAL REFERENCES

(1) Torres-Huerta, A., Velásquez-Hernández, M.D.J., Tamarit-Amoros, E., Raschetti, M., Pinkas, D., Jurček, O., Pérez, J., and Valkenier, H. (2025). Spatiotemporal Control of the Formation of Luminescent Lanthanide Complexes in Liposome-Based Nanoreactors. *Angew. Chem. Int. Ed.*, e202510471. https://doi.org/10.1002/anie.202510471.

(2) Brouwer, A.M. (2011). Standards for photoluminescence quantum yield measurements in solution (IUPAC Technical Report). Pure Appl. Chem. *83*, 2213–2228. https://doi.org/10.1351/PAC-REP-10-09-31.

(3) Briois, V., Fonda, E., Belin, S., Barthe, L., La Fontaine, C., Langlois, F., Ribbens, M., and Villain, F. (2011). SAMBA: The 4–40 keV X-ray absorption spectroscopy beamline at SOLEIL. In UVX 2010 - 10e Colloque sur les Sources Cohérentes et Incohérentes UV, VUV et X ; Applications et Développements Récents (EDP Sciences), pp. 41–47. https://doi.org/10.1051/uvx/2011006.

(4) Landrot, G., and Fonda, E. (2025). *Fastosh* : a software for the treatment of XAFS datasets of environmental relevance or acquired in *operando* conditions. *J. Synchrotron Radiat. 32*, 1085–1094. https://doi.org/10.1107/S1600577525003923.

(5) Newville, M. (2013). Larch: An Analysis Package for XAFS and Related Spectroscopies. *J. Phys. Conf. Ser. 430*, 012007. https://doi.org/10.1088/1742-6596/430/1/012007.

(6) Rehr, J.J., Kas, J.J., Prange, M.P., Sorini, A.P., Takimoto, Y., and Vila, F. (2008). Ab initio theory and calculations of X-ray spectra. *Comptes Rendus Phys. 10*, 548–559. https://doi.org/10.1016/j.crhy.2008.08.004.

(7) Chong, S., Riley, B.J., and Nelson, Z.J. (2020). Dehydration synthesis and crystal structure of terbium oxychloride, TbOCl. *Acta Crystallogr. Sect. E Crystallogr. Commun. 76*, 621–624. https://doi.org/10.1107/S2056989020004387.

(8) Martinez-Gomez, N. C.; Vu, H. N.; Skovran, E. Lanthanide Chemistry: From Coordination in Chemical Complexes Shaping Our Technology to Coordination in Enzymes Shaping Bacterial Metabolism. *Inorg. Chem.* 2016, *55* (20), 10083–10089. https://doi.org/10.1021/acs.inorgchem.6b00919.

(9) Boglaienko, D.; Andersen, A.; Heald, S. M.; Varga, T.; Mortensen, D. R.; Tetef, S.; Seidler, G. T.; Govind, N.; Levitskaia, T. G. X-Ray Absorption Spectroscopy of Trivalent Eu, Gd, Tb, and Dy Chlorides and Oxychlorides. *J. Alloys Compd.* 2022, *897*, 162629. https://doi.org/10.1016/j.jallcom.2021.162629.
