# Supplementary material for: Color-tunable luminescent TbxEuy(BDC) complexes assembled within liposome-based nanoreactors
Source: Cell Rep Phys Sci. 2026 May 20;7(5):103312. doi: 10.1016/j.xcrp.2026.103312 (PMC13190561; doi:10.1016/j.xcrp.2026.103312)
Supplement: Document S2. Article plus supplemental information [file mmc2.pdf]

Cell Reports
Physical Science

Article

Color-tunable luminescent Tb_xEu_y(BDC) complexes assembled within liposome-based nanoreactors

Graphical abstract

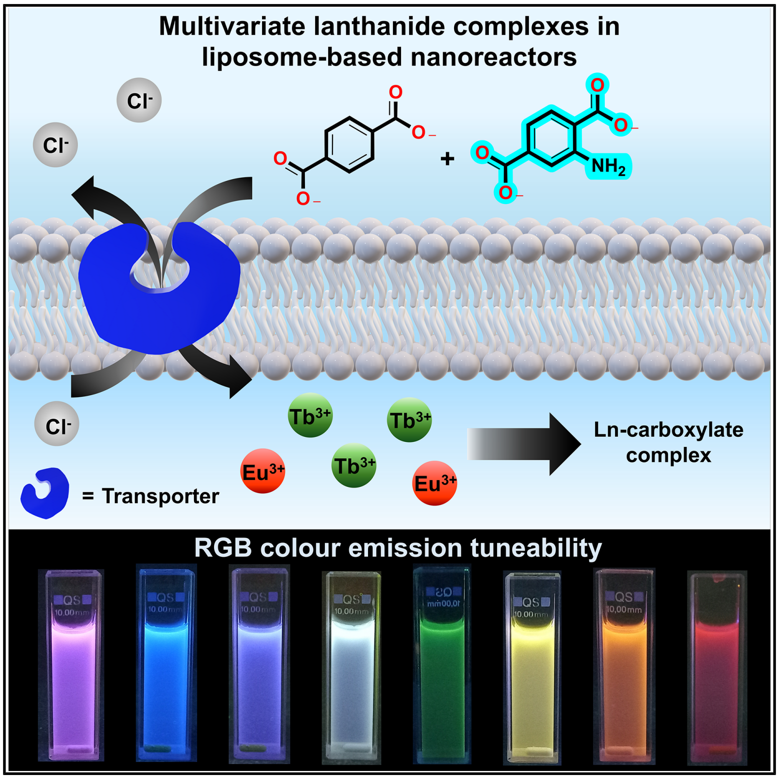

Authors

Aaron Torres-Huerta,
Miriam de J. Velásquez-Hernández,
Sven Lempereur, Ludovic Troian-Gautier,
Giulia Veronesi, Hennie Valkenier

Correspondence

aaron.torres.huerta@ulb.be (A.T.-H.),
hennie.valkenier@ulb.be (H.V.)

In brief

Aaron Torres-Huerta et al. develop liposome nanoreactors that assemble multimetallic lanthanide complexes inside attoliter compartments. Real-time photoluminescence monitoring reveals distinct Tb^{3+} and Eu^{3+} emission behavior, while ligand engineering enables RGB color tuning, providing a platform for studying coordination chemistry and designing luminescent nanomaterials in aqueous environments.

Highlights

- Liposome nanoreactors enable coordination reactions with controlled Tb/Eu ratios
- RGB emission color tuning is achieved via ligand and metal composition control
- Colloidal emissive systems are stable in aqueous solutions

Torres-Huerta et al., 2026, Cell Reports Physical Science 7, 103312
May 20, 2026 © 2026 The Authors. Published by Elsevier Inc.
https://doi.org/10.1016/j.xcrp.2026.103312

CellPress

Cell Reports
Physical Science

CellPress
OPEN ACCESS

Article

Color-tunable luminescent Tb_xEu_y(BDC) complexes assembled within liposome-based nanoreactors

Aaron Torres-Huerta,[1,*] Miriam de J. Velásquez-Hernández,[2] Sven Lempereur,[3] Ludovic Troian-Gautier,[3,4] Giulia Veronesi,[5] and Hennie Valkenier[1,4,6,*]

[1]Université libre de Bruxelles, Engineering of Molecular NanoSystems, Avenue F.D. Roosevelt 50, CP165/64, 1050 Brussels, Belgium
[2]Center for Membrane Separations, Adsorption, Catalysis, and Spectroscopy (cMACS), KU Leuven, 3001 Leuven, Belgium
[3]Institut de la Matière Condensée et des Nanosciences (IMCN), Molecular Chemistry, Materials and Catalysis (MOST), Université catholique de Louvain (UCLouvain), 1348 Louvain-la-Neuve, Belgium
[4]WEL Research Institute, 6 Avenue Pasteur, 1300 Wavre, Belgium
[5]University Grenoble Alpes, CNRS, CEA Grenoble, IRIG, Laboratoire de Chimie et Biologie des Métaux, 17 Rue des Martyrs, 38000 Grenoble, France
[6]Lead contact
*Correspondence: aaron.torres.huerta@ulb.be (A.T.-H.), hennie.valkenier@ulb.be (H.V.)
https://doi.org/10.1016/j.xcrp.2026.103312

SUMMARY

Precise stoichiometric control in multimetallic lanthanide nanosystems is essential for optical devices, sensing, and bioimaging applications owing to their composition-dependent emission properties. However, controlling metal composition, spatial distribution, intermetallic energy transfer, and colloidal stability remains challenging. Here, we report a liposome-based nanoreactor platform that enables the *in situ* formation of multivariate Tb_xEu_y-dicarboxylate complexes, enabling finely tuned lanthanide stoichiometry within attoliter-scale confined volumes. Liposomes pre-loaded with specific Tb^{3+}:Eu^{3+} ratios are combined with a synthetic anion transporter that mediates dicarboxylate transport through lipid membranes, enabling controlled coordination reactions in aqueous solution. This method, coupled with the use of a blue-emissive ligand, supports continuous color tuning across the entire RGB spectrum. Real-time emission spectroscopy reveals faster photoluminescence appearance for Eu^{3+} than Tb^{3+}, providing experimental insight into lanthanide reactivity under nanoscale confinement. These findings position liposome-based nanoreactors as a versatile platform for investigating coordination reactions and engineering multimetallic luminescent colloidal materials in aqueous media.

INTRODUCTION

Lanthanide-based systems possess exceptional luminescent properties, making them uniquely suited for optical applications.[1–3] These properties arise from the open-shell 4f orbitals of lanthanides, resulting in narrow emission bands, high color purity, and long luminescence lifetimes. Precise control over emission color is essential for advancing a wide range of emerging technologies, including information encryption,[4,5] light-emitting diodes (LEDs),[6] optical thermometers,[7] sensing,[8] and bioimaging.[2,9]

The rational integration of multiple lanthanide ions, such as terbium (Tb^{3+}) and europium (Eu^{3+}), into a single system enables multicolor emission spanning the visible spectrum.[10] However, the formation of multivariate lanthanide complexes remains challenging, and the major limitation lies in the difficulty of distinguishing genuine heterometallic assemblies from physical mixtures or phase-segregated domains.[11] Moreover, intermetallic energy transfer from Tb^{3+} to Eu^{3+}, commonly observed in solid-state materials such as metal-organic frameworks (MOFs),[12] often leads to quenching of Tb-based green emission, compromising color tunability and spectral precision. Furthermore, the stoichiometric ratios of lanthanide precursors do not always reflect the final product composition,[13] introducing further variability and uncertainty in the design of multimetallic emitters. To mitigate undesired energy transfer and improve control over emission profiles, strategies such as core-shell nanoparticles,[14–16] core-shell MOFs,[13,17–19] and layer-by-layer films[20,21] have been employed to spatially isolate different lanthanides. Although effective, these approaches often involve sophisticated fabrication protocols and are generally limited to solid-state systems, hindering their use in aqueous or biologically relevant environments.

To address these challenges and enable the formation of water-compatible, luminescent nanomaterials with color-tunable properties, we propose the on-demand assembly of lanthanide-carboxylate complexes within liposomes. Drawing inspiration from synthetic ion transport strategies commonly applied in the context of channelopathies,[22,23] we have pioneered the controlled transmembrane transport of organic ligands into liposomes pre-loaded with selected metal ions to drive the formation of hybrid metal-organic colloidal materials.[24]

© 2026 The Authors. Published by Elsevier Inc.
This is an open access article under the CC BY license (http://creativecommons.org/licenses/by/4.0/).

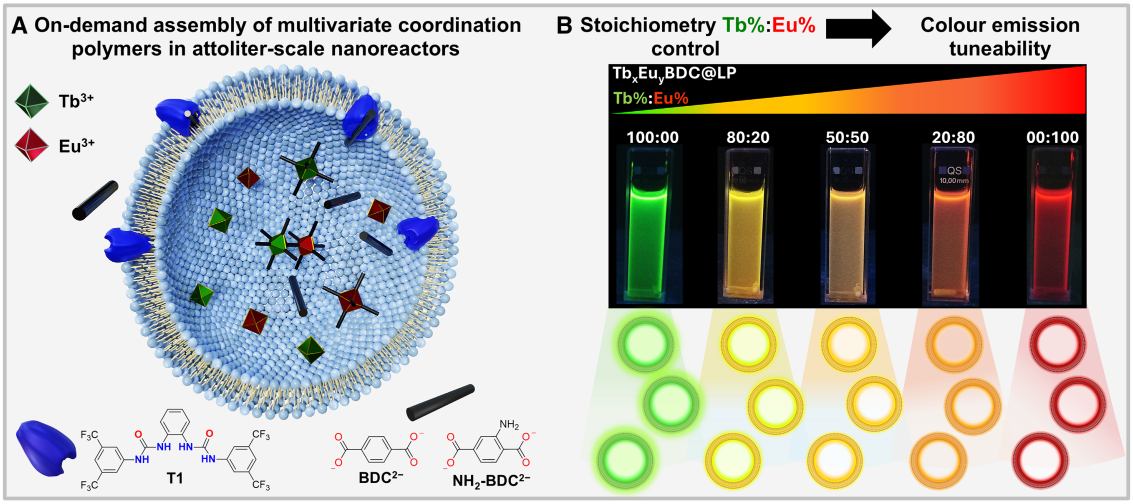

Figure 1. Liposome nanoreactor strategy to control multivariate lanthanide complex formation

(A) Schematic illustration of the liposome-based nanoreactor (LNR) strategy, showing the controlled transmembrane transport of benzene-1,4-dicarboxylate (BDC^{2-}) and subsequent coordination with encapsulated lanthanide cations inside 200-nm liposomes.

(B) Tuning of Tb^{3+}:Eu^{3+} ratios to achieve color-tunable luminescent nanomaterials in aqueous solution.

In this work, we exploit the liposome-based nanoreactor (LNR) platform to tune the luminescent properties of colloidal multivariate Tb_xEu_y(BDC) complexes assembled within attoliter (10^{-18} L) confined volumes (Tb_xEu_yBDC@LP; LP, liposome). This level of synthetic control is achieved by encapsulating predefined Tb^{3+}:Eu^{3+} ratios in liposomes, followed by the assisted transport of benzene-1,4-dicarboxylate (BDC^{2-}) using a bisurea-based transporter (**T1**), to afford colloidal Tb_xEu_yBDC@LP systems with a bilamellar structure (Figures 1 and S2; Note S1). This methodology offers precise control over the stoichiometry ratios of encapsulated lanthanide ions, and the fluorescence properties of the lanthanides allow for real-time monitoring via fluorescence spectroscopy. The successful formation of co-encapsulated Tb_xEu_yBDC@LP systems can be confirmed by steady-state photoluminescence spectroscopy using the distinct emission profiles of both lanthanides. Real-time tracking of the 542 (from Tb^{3+}) and 614 (from Eu^{3+}) nm emission bands allows us to study the emission evolution kinetics of Eu^{3+} and Tb^{3+} in competitive mixtures, providing direct experimental insight into the reactivity of lanthanides in confined nanospaces. Furthermore, by employing the blue-emissive 2-amino-1,4-benzenedicarboxylate (NH_2BDC^{2-}) ligand, the system could be extended to achieve full RGB color tunability.

RESULTS AND DISCUSSION

Preparation and characterization of luminescent multivariate Tb_xEu_yBDC@LP nanosystems

Liposomes were prepared using a 7:3 molar ratio of 1-palmitoyl-2-oleoyl-*sn*-glycero-3-phosphocholine (POPC) and cholesterol. The lipid film was hydrated with a 7 mM aqueous solution of $TbCl_3$ and $EuCl_3$ in predefined molar ratios (Tb:Eu = 100:00, 90:10, 80:20, 70:30, 60:40, 50:50, 40:60, 30:70, 20:80, 10:90, and 00:100). Then, lipids were subjected to freeze-thaw cycles and extrusion through a polycarbonate membrane with 200 nm pores to afford uniform $Tb_xEu_yCl_3$@LP vesicles. Non-encapsulated lanthanide ions and chloride were removed by dialysis against a 10.5 mM Na_2SO_4 solution. The resulting vesicle suspensions were diluted in Na_2SO_4 medium to achieve a final lipid concentration of 3 mM.

The encapsulation efficiency was confirmed using a chloride ion-selective electrode (Cl-ISE) by measuring the chloride concentration in a 3 mL liposomal suspension. All $Tb_xEu_yCl_3$@LP samples displayed residual chloride levels below 0.07 mM. After lysing the liposomes with Triton X-100, the chloride concentration increased significantly (0.36–0.45 mM; Figure S1; Methods S1), which corresponds to an estimated total lanthanide concentration of 0.12–0.15 mM. This confirmed that chloride and, consequently, the lanthanide ions were successfully encapsulated.

To induce the *in situ* formation of lanthanide-organic complexes within the liposomes, BDC^{2-} was transported through the membrane by the anion transporter **T1** (Figure 1A) at a transporter-to-lipid molar ratio of 1:10,000. The integration of **T1** in the lipid membrane triggers the dissipation of the BDC^{2-} gradient via the BDC^{2-}/Cl^{-} ion-exchange process.[25] Dynamic light scattering (DLS) measurements confirmed that the resultant Tb_xEu_yBDC@LP systems exhibit a monodisperse size distribution and high colloidal stability, with an average hydrodynamic diameter of approximately 170 nm (Figures 2A and S3), which is consistent with our previous reports.[24] Additionally, scanning electron microscopy coupled with energy-dispersive X-ray spectroscopy (SEM-EDX; Methods S2) analysis of selected samples revealed the co-localization of both Tb^{3+} and Eu^{3+} within individual liposomes (Figure S4).

The successful formation of the luminescent Tb_xEu_y(BDC) complex inside the liposomes was confirmed by steady-state photoluminescence spectroscopy in solution. Emission spectra were recorded from 450 to 750 nm upon excitation at λ_{ex} = 285 nm, using a 450 nm long-pass filter. The excitation

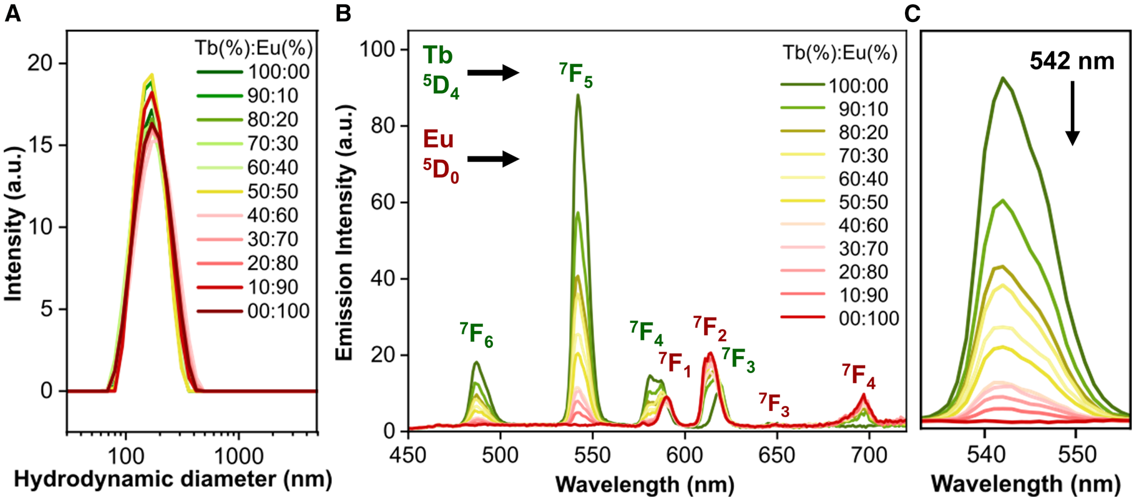

Figure 2. Characterization of $Tb_xEu_yBDC@LP$ systems

(A) DLS measurements of $Tb_xEu_yBDC@LP$ samples in aqueous Na_2SO_4 solution.

(B) Emission spectra of $Tb_xEu_yBDC@LP$ samples upon excitation at 285 nm, displaying characteristic terbium and europium transitions.

(C) Zoom of the emission band around 542 nm as a function of terbium mole fraction.

wavelength was selected based on the excitation spectra of reference samples $Tb_{100}Eu_{00}BDC@LP$ and $Tb_{00}Eu_{100}BDC@LP$ (Figure S5). The $Tb_{100}Eu_{00}BDC@LP$ sample displayed characteristics terbium transitions $^5D_4 \rightarrow {}^7F_j$ (j = 6, 5, 4, and 3), while the $Tb_{00}Eu_{100}BDC@LP$ sample exhibited europium transitions $^5D_0 \rightarrow {}^7F_j$ (j = 1–4), which are consistent with carboxylate coordination (Figures S6 and S8).[26] Samples containing both Tb^{3+} and Eu^{3+} ions displayed both sets of transitions, in agreement with the co-encapsulation and implication of both lanthanides in the coordination reaction with BDC^{2-} (Figures 2B and S9).

Notably, the terbium transitions $^5D_4 \rightarrow {}^7F_j$ exhibited higher emission intensity than the europium transitions $^5D_0 \rightarrow {}^7F_j$. This trend is consistent with the generally more efficient sensitization of Tb^{3+} by BDC^{2-} ligands than of Eu^{3+}, as reported for related lanthanide-carboxylate systems.[27] To further support this interpretation, we measured the photoluminescence quantum yields of representative samples (see Methods S3). The $Tb_{100}Eu_{00}BDC@LP$, $Tb_{00}Eu_{100}BDC@LP$, and $Tb_{50}Eu_{50}BDC@LP$ systems exhibited quantum yields of Φ = 0.049, 0.016, and 0.011, respectively. Interestingly, the strongest Tb^{3+} emission at 542 nm ($^5D_4 \rightarrow {}^7F_5$) significantly decreased with increasing Eu^{3+} contents (Figure 2C), consistent with quenching via intermetallic energy transfer from Tb^{3+} to Eu^{3+} (Figure S6). This behavior is in line with previously reported $Tb^{3+} \rightarrow Eu^{3+}$ energy transfer in mixed-lanthanide MOFs[12,28] and molecular lanthanide systems.[29]

Comparison of the emission properties of co-encapsulated $Tb_xEu_yBDC@LP$ systems and controlled mixtures of monometallic systems

To gain deeper insights into the intermetallic energy transfer from Tb^{3+} to Eu^{3+} in multivariate systems, we also prepared physical mixtures of $Tb_{100}Eu_{00}BDC@LP$ and $Tb_{00}Eu_{100}BDC@LP$ samples in mole fractions that matched the Tb:Eu ratios used in the $Tb_xEu_yBDC@LP$ series. These mixtures ensured equivalent total lanthanide concentrations but maintained spatial separation between Tb^{3+} and Eu^{3+} within distinct liposomes, thereby minimizing direct intermetallic interactions.

Under UV illumination at 254 nm, these differences were visually apparent. The co-encapsulated $Tb_xEu_yBDC@LP$ samples, in which intermetallic energy transfer is active, exhibited a color gradient from green (Tb rich) to red (Eu rich), with prominent yellow to orange hues observed at intermediate compositions (Tb:Eu = 80:20–10:90) (Figure 3A). In contrast, the physically mixed systems, which maintained spatial separation between lanthanides and thereby suppressed energy transfer, displayed predominantly green-yellow emission at Tb:Eu ratios of 100:00–40:60 (Figure 3B), with significantly reduced orange-red colors.

Quantitative analysis of the emission intensities at 542 (from Tb^{3+}) and 614 (from Eu^{3+}) nm revealed pronounced differences between co-encapsulated (Figure 3C) and physically mixed (Figures 3D and S10) systems. In the $Tb_xEu_yBDC@LP$ series, terbium emission showed an exponential decrease, while europium emission increased logarithmically with increasing Eu^{3+} mole fraction (Figures 3C and S11). This behavior is consistent with the intermetallic energy transfer from Tb^{3+} to Eu^{3+} within the confined environment of the liposomes. In contrast, physical mixtures displayed linear emission trends for both lanthanides (Figures 3D and S12). This linearity indicates a simple dilution effect rather than an energy transfer process, suggesting the presence of independent, non-interacting luminescent species.

These findings demonstrate that spatial confinement within liposomes enables controlled localization and distribution of lanthanide ions, thereby governing intermetallic energy transfer and resulting in tunable luminescence. Notably, while co-encapsulation promotes energy-transfer-based modulation, physical mixtures yield more predictable and finely tunable color due to the absence of intermetallic coupling. Overall, the liposomal membrane functions as a versatile nanoscale scaffold that

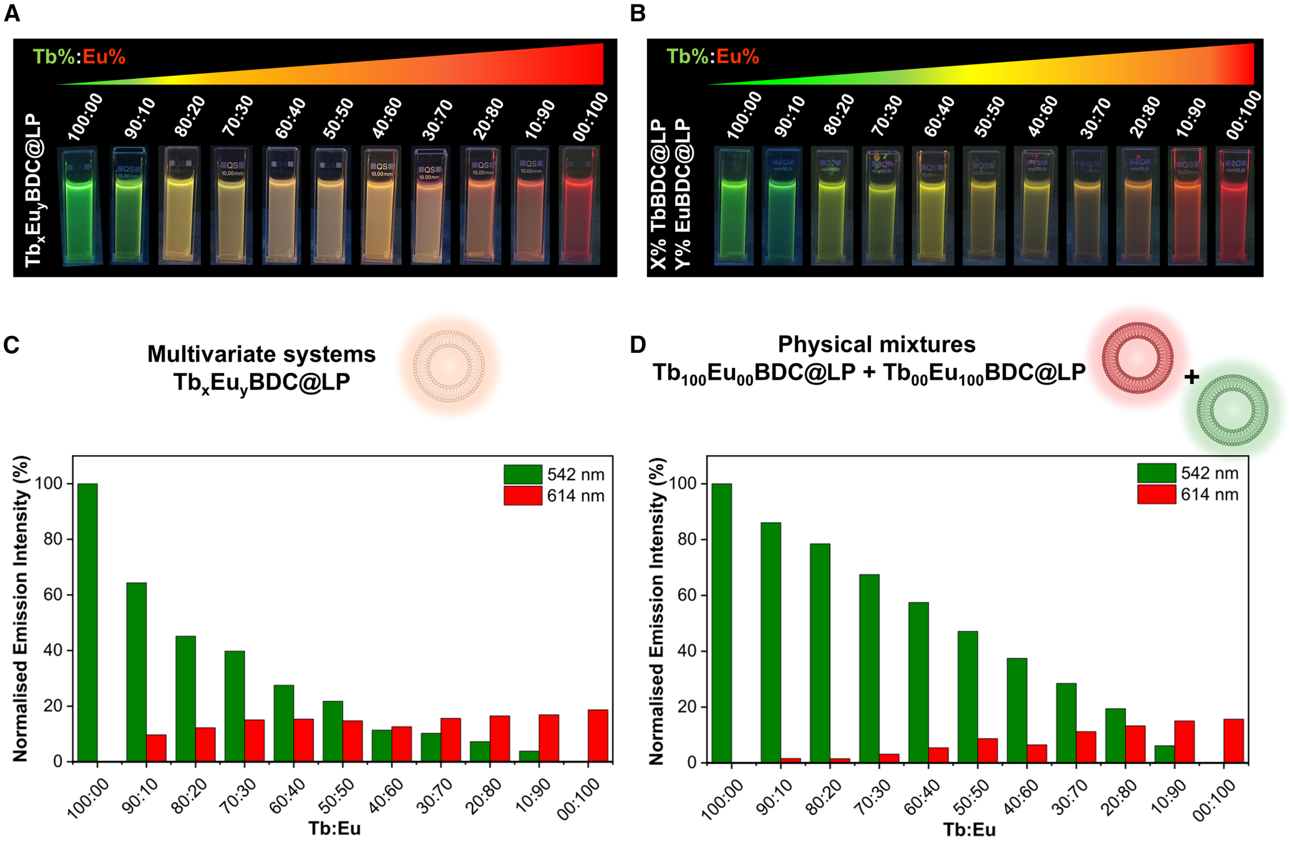

Figure 3. Emission properties of co-encapsulated lanthanides and physically mixed systems

(A and B) Photographs of liposome suspensions under 254-nm UV illumination, showing tunable emission colors in (A) the Tb$_x$Eu$_y$BDC@LP series and (B) their analog physical mixtures.

(C) Emission intensities for Tb$_x$Eu$_y$BDC@LP samples collected at 542 (Tb^{3+}, green) and 614 (Eu^{3+}, red) nm reveal an exponential decrease in terbium emission and a corresponding increase in europium emission with rising Eu^{3+} content.

(D) Emission intensities for physical mixtures of Tb$_{100}$Eu$_{00}$BDC@LP and Tb$_{00}$Eu$_{100}$BDC@LP at equivalent Tb:Eu mole fractions for reference.

supports either synergistic or independent luminescent emission, depending on the spatial arrangement of the lanthanide ions.

Monitoring lanthanide emission appearance in lipid nanoreactors with real-time steady-state photoluminescence spectroscopy

To further assess changes in the emission behavior of Tb^{3+} and Eu^{3+} within the LNRs, we performed real-time steady-state photoluminescence measurements. Emission intensities at 542 (Tb^{3+}, $^5D_4 \rightarrow {}^7F_5$) and 614 ($Eu^{3+}$, $^5D_0 \rightarrow {}^7F_2$) nm were simultaneously recorded over 30 min, upon the addition of BDC^{2-}. These measurements allowed us to monitor both the transmembrane transport of BDC^{2-} within the vesicle interior and the subsequent linker coordination with the encapsulated lanthanide ions.

Initial experiments were performed on the single-lanthanide reference samples Tb$_{100}$Eu$_{00}$BDC@LP and Tb$_{00}$Eu$_{100}$BDC@LP, using **T1** at a 1:10,000 transporter-to-lipid molar ratio. Upon BDC^{2-} addition, the europium-containing sample exhibited an immediate increase in emission intensity at 614 nm, indicating rapid complex formation (Figure 4F). In contrast, the terbium-containing sample displayed a delayed onset emission at 542 nm, with a pronounced increase occurring ~5 min post-addition (Figure 4A). These differences could, for instance, be due to intrinsic variations in the coordination kinetics of Eu^{3+} and Tb^{3+} with carboxylate ligands or to different thresholds in local coordination geometry required for each lanthanide to become efficiently emissive.

In multivariate systems such as the Tb$_{80}$Eu$_{20}$BDC@LP sample (Figure 4B), the initial emission was dominated by the Eu^{3+} band centered at 614 nm, which increased faster and reached a plateau earlier than the emission associated with Tb^{3+} at 542 nm. Thus, despite the higher Tb^{3+} mole fraction, europium emission clearly appeared faster. This trend persisted across all Tb$_x$Eu$_y$BDC@LP samples (Figures 4B–4E and S13–S18), where the emission of europium increased consistently at a faster rate than that of terbium. Photoluminescence data across the Tb$_x$Eu$_y$BDC@LP series revealed a systematic reduction in the Tb^{3+} signal intensity and a corresponding increase in the Eu^{3+} signal as the mole fraction of Eu^{3+} increased. Furthermore, the presence of quenching throughout the kinetic profiles of

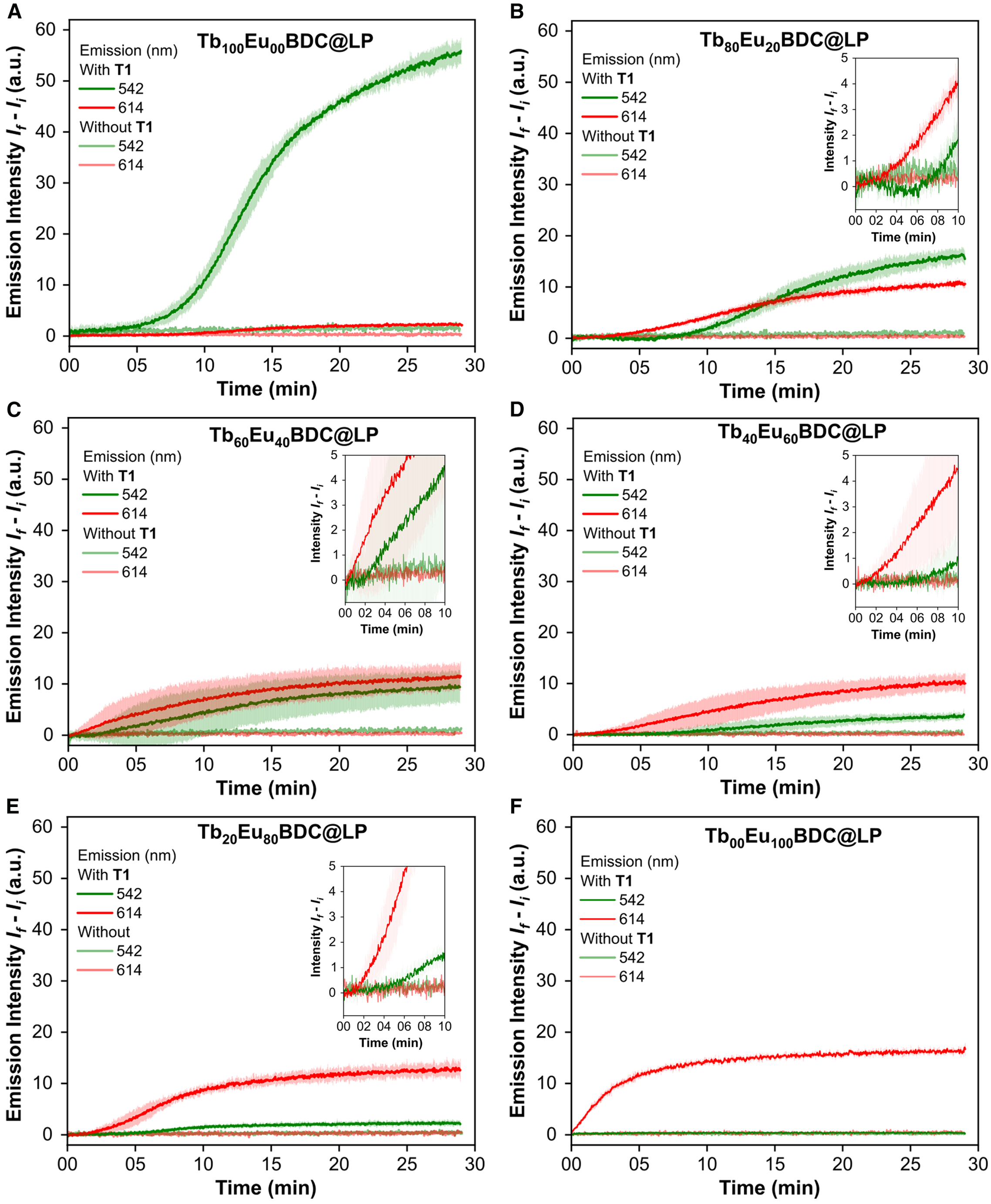

Figure 4. Real-time monitoring of emission changes following BDC^{2-} addition

(A–F) Emission intensities at 542 nm (green line) correspond to the Tb^{3+} $^5D_4 \rightarrow {}^7F_5$ transition, while those at 614 nm (red line) correspond to the Eu^{3+} $^5D_0 \rightarrow {}^7F_2$ transition. Kinetic data were collected for (A) $Tb_{100}Eu_{00}$BDC@LP, (B) $Tb_{80}Eu_{20}$BDC@LP, (C) $Tb_{60}Eu_{40}$BDC@LP, (D) $Tb_{40}Eu_{60}$BDC@LP, (E) $Tb_{20}Eu_{80}$BDC@LP, and (F) $Tb_{00}Eu_{100}$BDC@LP. Each image includes control experiments without transporter **T1**, represented by light green and light red. Shaded areas represent the standard deviation of three independent measurements.

Tb_xEu_yBDC@LP systems suggests the formation of a multimetallic coordination complex.

The earlier appearance of Eu^{3+} compared to Tb^{3+} emission may arise not only from faster coordination kinetics, as in the present system, the time-dependent photoluminescence response reflects the convolution of several coupled processes. These include the evolution of ligand-to-metal sensitization pathways and structural rearrangements of the coordination assemblies

to obtain strong emission. To understand such contributions in a quantitative manner would require detailed theoretical modeling and/or advanced kinetic analysis, which are beyond the scope of the present work.

Control experiments conducted in the absence of **T1** confirmed that no change in emission intensity occurred upon BDC^{2-} addition, supporting the role of **T1** in actively mediating ligand transport and indicating the integrity of the liposomal membrane, confirming the absence of Ln^{3+} leakage and the impermeability of the lipid membrane to BDC^{2-} in the absence of an anion transporter.

We have attempted to study the changes in the coordination of Tb^{3+} during the formation of $Tb_{100}Eu_{00}BDC@LP$ systems in more detail using X-ray absorption spectroscopy (XAS; see Methods S4 and Note S3). While the Tb^{3+} signal was clearly observed, the changes in the coordination sphere were too small to result in significant differences in the near-edge region of the spectra (Figure S29A), and only minor differences were detected in the extended X-ray absorption fine structure (EXAFS) spectra (Figures S29B and S30; Table S1). This can be explained by the first coordination sphere most likely being made up exclusively of oxygen atoms throughout the coordination process. At the start, the encapsulated Tb^{3+} will be solvated by water molecules, with potential interactions with phosphate groups from the lipids, and after the transport of BDC^{2-} into the liposomes, the water molecules are (partially) replaced by the coordinating carboxylate groups. The coordination of the carboxylates to Tb^{3+} or Eu^{3+} cations in the liposomes was confirmed by infrared spectroscopy (see Methods S5; Note S4; Figures S31 and S32).

Extending emission to cover the RGB spectrum

To expand the potential versatility of our system, we used the blue-emissive ligand NH_2BDC^{2-} to achieve full RGB color coverage, which represents an essential feature for advanced display technologies and optical probes within biological environments.[30,31] NH_2BDC^{2-} is known to exhibit blue fluorescence (∼460–480 nm; Figure S19) in ligand-based MOF systems,[32,33] making it a suitable complement to the green (Tb^{3+}) and red (Eu^{3+}) emissions within our nanoreactor platform.

Initial attempts to form $Tb(NH_2BDC)@LP$ and $Eu(NH_2BDC)@LP$ resulted exclusively in intense blue emission, with no detectable Tb^{3+} or Eu^{3+} transitions, indicating that the NH_2BDC ligand strongly quenches lanthanide-based emission. This behavior is consistent with previous reports on Ln-NH_2BDC-based MOFs suspended in dry organic solvents, where even small amounts of water (<5%) cause significant quenching of lanthanide emission due to Ln^{3+}→ligand energy back-transfer processes.[34,35] To minimize quenching, the excess of unbound ligand was removed by introducing an additional post-complexation dialysis step for the $Ln(NH_2BDC)@LP$ and $Ln(BDC)(NH_2BDC)@LP$ systems (see the methods section).

Mixed-ligand systems with varying BDC^{2-}:NH_2BDC^{2-} mole fractions were then prepared (Figure 5A). Compositions containing ≥20% NH_2BDC^{2-} significantly suppressed lanthanide emission (Figures S21 and S22), whereas samples containing ≤10% NH_2BDC^{2-} retained characteristic Tb^{3+} and Eu^{3+} bands. Based on these results, selected reference compositions exhibiting green ($Tb_{100}Eu_{00}BDC@LP$), yellow ($Tb_{80}Eu_{20}BDC@LP$), orange ($Tb_{30}Eu_{70}BDC@LP$), and red ($Tb_{00}Eu_{100}BDC@LP$) emissions were further modified by replacing the ligand pulse with BDC^{2-}:NH_2BDC^{2-} mixtures at 95:05 and 90:10 ratios (Figures 5B and 5C). In all cases, a reduction in lanthanide emission intensity was observed, more pronounced for Tb^{3+} than Eu^{3+} (Figures S23–S26), while blue emission from NH_2BDC^{2-} became dominant. The resulting chromaticity enabled the acquisition of additional colors, such as gray-blue ($Tb_{100}Eu_{00}(BDC)_{95}(NH_2BDC)_{05}@LP$) and pink ($Tb_{80}Eu_{20}(BDC)_{95}(NH_2BDC)_{05}@LP$). Although the additional post-complexation dialysis step and Fourier transform infrared (FTIR) measurements (Figures S31 and S32) support coordination of NH_2BDC^{2-} to the lanthanide cations, the confined and dynamic environment inside liposome nanoreactors may allow the coexistence of multiple coordination species. Therefore, the present experiments cannot completely exclude the presence of mixed assemblies or minor fractions of uncoordinated ligand. Additionally, DLS analysis confirmed that the introduction of NH_2BDC^{2-} did not affect colloidal integrity, as all samples remained monodisperse with hydrodynamic diameters comparable to the $Tb_xEu_yBDC@LP$ samples (Figures S21B and S22B).

Furthermore, to explore whether spatial separation could mitigate quenching, physical mixtures were prepared using $Tb_{100}Eu_{00}BDC@LP$ and the highly violet-emissive $Tb_{00}Eu_{100}(BDC)_{95}(NH_2BDC)_{05}@LP$. As anticipated, the liposomal membrane prevented detrimental quenching of the Tb^{3+} transitions, and the 542 nm emission band decreased linearly with the volume fraction of the violet-emissive partner, consistent with a dilution effect (Figure S27). This mixing strategy enabled a controlled color shift from green to cyan and ultimately to near-white emission at a 70:30 ratio (Figure S28), demonstrating precise RGB balance through nanoscale spatial control.

In summary, this study establishes LNRs as a powerful and versatile platform for the synthesis and study of multimetallic lanthanide complexes in aqueous conditions. By precisely controlling the Tb^{3+}:Eu^{3+} stoichiometry and spatial co-localization within attoliter-scale vesicles, we achieved the *in situ* formation of $Tb_xEu_y(BDC)$ complexes with composition-dependent, tunable emission profiles. The use of a synthetic bisurea as an anion transporter enabled controlled ligand delivery and real-time steady-state photoluminescence monitoring, revealing that Eu^{3+} exhibits faster emission increases than Tb^{3+} under nanoscale confinement. Comparative studies with physically mixed monometallic systems confirmed that efficient Tb→Eu energy transfer requires co-encapsulation and demonstrated the suitability of LNRs to resolve subtle differences in reactivity and complexation. Extending this strategy to blue-emissive NH_2BDC^{2-} ligands enables full RGB coverage, expanding the photonic versatility of the platform. Notably, the liposomal membrane ensures colloidal stability, prevents aggregation, and provides a biologically relevant aqueous environment. Overall, this study highlights LNRs as an adaptable nanoscale scaffold for engineering multimetallic luminescent nanomaterials with precisely controlled spatial organization and optical output.

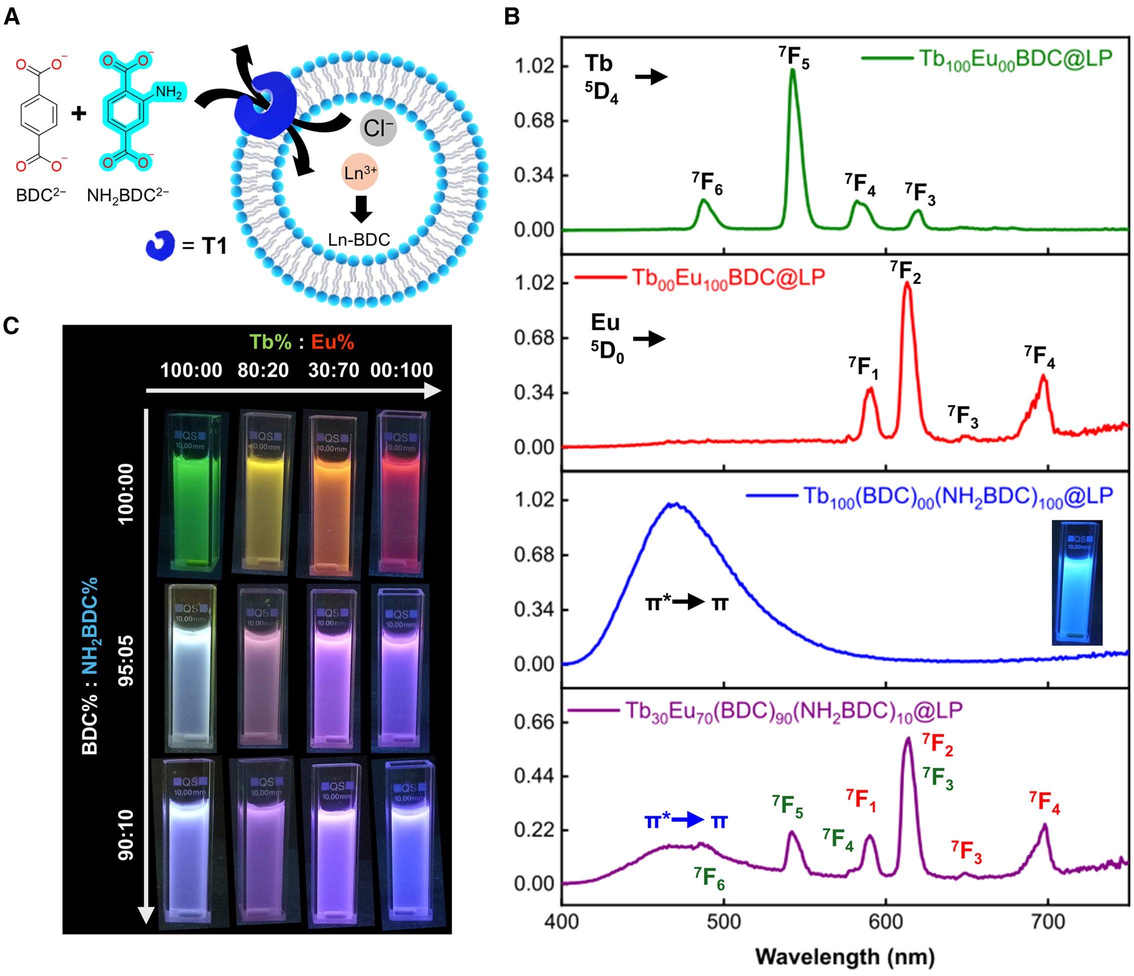

Figure 5. RGB emission tuning using mixed-ligand systems

(A) Extension of emission tunability to cover the full RGB spectrum through the use of mixed BDC^{2-}/NH_2BDC^{2-} ligand pulses.

(B) Emission spectra of representative systems: $Tb_{100}Eu_{00}BDC$@LP, $Tb_{00}Eu_{100}BDC$@LP, $Tb_{100}(BDC)_{00}(NH_2BDC)_{100}$@LP, and $Tb_{30}Eu_{70}(BDC)_{90}(NH_2BDC)_{10}$@LP.

(C) Mixed-ligand systems generated by replacing the ligand pulse with BDC^{2-}/NH_2BDC^{2-} mixtures (95:05 and 90:10), expanding chromaticity. Photographs are of liposome suspensions under 254-nm UV illumination.

METHODS

General experimental information

The reagents and solvents were obtained from Sigma-Aldrich and used without further purification unless otherwise specified. POPC and cholesterol were purchased from Sigma-Aldrich and Acros, respectively. Chloroform was deacidified before preparing lipid solutions by passing it through a column containing basic alumina. POPC solutions were stored at −20°C, while cholesterol solutions were freshly prepared. All aqueous solutions were prepared using deionized water, and the pH values were 6.5–7.

Liposome preparation

Liposomes were prepared from a lipid mixture of POPC and cholesterol at a 7:3 molar ratio, dissolved in deacidified chloroform. The total lipid amount was calculated to achieve a final concentration of 3 mM (POPC + cholesterol). Chloroform was removed by evaporation under a gentle stream of nitrogen, followed by overnight drying under vacuum to ensure complete solvent removal. The dried lipid film was rehydrated with 500 μL of a 7 mM aqueous solution containing $TbCl_3$ and $EuCl_3$ in predefined molar ratios (Tb:Eu = 100:00, 90:10, 80:20, 70:30, 60:40, 50:50, 40:60, 30:70, 20:80, 10:90, and 00:100). The resulting suspension was sonicated for 1 min and stirred for 1 h at room temperature to promote vesicle formation. To obtain predominantly unilamellar vesicles, the suspension underwent 10 freeze-thaw cycles, followed by extrusion 29 times through polycarbonate membranes with a pore size of 200 nm at room temperature.

Unencapsulated lanthanide chloride ($LnCl_3$) outside the liposomes was removed by dialysis using Biotech CE Tubing dialysis membranes (molecular weight cut-off [MWCO] 20 kDa) against a 10.5 mM Na_2SO_4 solution. The final $LnCl_3$@LP suspension was then diluted in the external solution to achieve a final lipid concentration of 3 mM for fluorescence experiments.

Liposome preparation for RGB systems

Liposomes were prepared following the methodology described above, with modifications introduced to minimize the quenching effect of the NH_2BDC^{2-} ligand. Specifically, the total concentration of the BDC^{2-}:NH_2BDC^{2-} mixture was reduced to 0.33 mM, and the resulting Ln(BDC)(NH_2BDC)@LP suspensions were diluted in the external solution to achieve a final lipid concentration of 1 mM. The concentration of transporter **T1** was maintained at a transporter-to-lipid molar ratio of 1:10,000. To remove excess unbound ligand remaining in the external phase after complex formation, a post-complexation purification step was included, consisting of dialysis against a 10.5 mM Na_2SO_4 solution using Biotech CE Tubing (MWCO 20 kDa) (three cycles, 150 mL each).

DLS

DLS measurements were carried out using a Malvern Zetasizer Ultra instrument at 25°C. Samples were loaded into disposable cuvettes, and measurements were performed using a standard refractive index of 1.45 for lipid-based systems.

Steady-state photoluminescence spectroscopy

Steady-state photoluminescence measurements were carried out using a Horiba Fluoromax-4. Emission spectra were recorded from 450 to 750 nm upon excitation at 285 nm, using a 450 nm long-pass filter (Figure S20; Note S2). All measurements were performed in quartz cuvettes containing a magnetic stir bar. The cuvette holder temperature was maintained at 25°C using a water-circulating bath, and the sample compartment was allowed to equilibrate for 3 min before each experiment.

For complexation experiments, 3 mL of a $LnCl_3$@LP suspension was placed in the cuvette, and **T1** (3.3 μL, 0.27 mM in methanol) was added to achieve a transporter-to-lipid molar ratio of 1:10,000. Then, 60 μL of a 50 mM BDC^{2-} solution was added, resulting in a final BDC^{2-} concentration of 1 mM. Emission spectra were recorded until no further changes in intensity were observed, typically within 1 h. Additionally, control experiments were conducted to verify that the emission results directly from the coordination of BDC^{2-} with the lanthanide cation inside liposomes (Figure S7). For kinetics experiments, the emission intensities at 542 and 614 nm were recorded simultaneously over a 30-min period following the addition of BDC^{2-} and **T1**.

Photographs

Photographs of the liposomal suspensions were captured using a Samsung A4 smartphone under illumination from a handheld UV lamp (254 nm). The images were taken inside a custom-built wooden black box to eliminate ambient light interference.[36–43]

RESOURCE AVAILABILITY

Lead contact

Requests for further information and experimental data should be directed to the lead contact, Hennie Valkenier (hennie.valkenier@ulb.be).

Materials availability

This study did not generate new unique reagents.

Data and code availability

- All data reported in this paper will be shared by the lead contact upon request.
- This paper does not report original code.
- Any additional information required to reanalyze the data reported in this paper is available from the lead contact upon request.

ACKNOWLEDGMENTS

This project has received funding from the European Union's Horizon Europe research and innovation programme under Marie Skłodowska-Curie grant agreement no. 101065037 and from the European Research Council (ERC) under grant agreement no. 802727 (Horizon 2020). Access to international research center laboratories and large-scale analytical facilities was supported by the European Union's Horizon 2020 research and innovation programme under grant agreement no. 101007417, having benefited from the access provided by the SAMBA station at the SOLEIL synchrotron in Paris, France, and ENL FEMTO-ST/CNRS - EuroNanoLab in Besançon, France, within the framework of the NFFA-Europe Pilot Transnational Access Activity, proposal ID520. We extend our gratitude to Dr. Andrea Zitolo for assisting with XAS data acquisition and to Elena Tamarit-Amoros for her support in sample preparation during synchrotron experiments. This work was partly supported by the French RENATECH network and its FEMTO-ST technological facility. We thank Marina Raschetti for her assistance with the acquisition and analysis of SEM-EDX data. H.V. is a research associate of the Fonds de la Recherche Scientifique (FNRS).

AUTHOR CONTRIBUTIONS

A.T.-H., M.d.J.V.-H., and H.V. conceptualized the study and designed the experiments. G.V. supervised, analyzed, and validated the synchrotron experiments and results. S.L. and L.T.-G. determined the photoluminescence quantum yields. A.T.-H. and M.d.J.V.-H. prepared the initial draft of the manuscript. H.V. reviewed and edited the manuscript. H.V. provided supervision and project administration. Funding acquisition was secured by H.V. and A.T.-H. All authors reviewed and approved the final version of the manuscript.

DECLARATION OF INTERESTS

The authors declare no competing interests.

SUPPLEMENTAL INFORMATION

Supplemental information can be found online at https://doi.org/10.1016/j.xcrp.2026.103312.

Received: November 10, 2025
Revised: February 27, 2026
Accepted: April 17, 2026
Published: May 8, 2026

REFERENCES

1. Zhou, B., Shi, B., Jin, D., and Liu, X. (2015). Controlling upconversion nanocrystals for emerging applications. Nat. Nanotechnol. *10*, 924–936. https://doi.org/10.1038/nnano.2015.251.
2. Alexander, C., Guo, Z., Glover, P.B., Faulkner, S., and Pikramenou, Z. (2025). Luminescent Lanthanides in Biorelated Applications: From Molecules to Nanoparticles and Diagnostic Probes to Therapeutics. Chem. Rev. *125*, 2269–2370. https://doi.org/10.1021/acs.chemrev.4c00615.
3. Zhao, D., Guo, L., Li, Q., Yue, C., Han, B., Liu, K., and Li, H. (2024). Multi-Functional Lanthanide Metallopolymer: Self-Healing and Photo-Stimuli-Responsive Dual-Emitting Luminescence for Diverse Applications. Adv. Mater. *36*, 2405164. https://doi.org/10.1002/adma.202405164.
4. Liu, Y., Zhao, K., Ren, Y., Wan, S., Yang, C., Li, J., Wang, F., Chen, C., Su, J., Chen, D., et al. (2022). Highly Plasticized Lanthanide Luminescence for

Information Storage and Encryption Applications. Adv. Sci. *9*, 2105108. https://doi.org/10.1002/advs.202105108.

5. Xie, Y., Sun, G., Li, J., and Sun, L. (2023). Multimode Emission from Lanthanide-Based Metal–Organic Frameworks for Advanced Information Encryption. Adv. Funct. Mater. *33*, 2303663. https://doi.org/10.1002/adfm.202303663.
6. Tang, Y., Wu, H., Cao, W., Cui, Y., and Qian, G. (2021). Luminescent Metal–Organic Frameworks for White LEDs. Adv. Opt. Mater. *9*, 2001817. https://doi.org/10.1002/adom.202001817.
7. Brites, C.D.S., Balabhadra, S., and Carlos, L.D. (2019). Lanthanide-Based Thermometers: At the Cutting-Edge of Luminescence Thermometry. Adv. Opt. Mater. *7*, 1801239. https://doi.org/10.1002/adom.201801239.
8. Sahoo, S., Mondal, S., and Sarma, D. (2022). Luminescent Lanthanide Metal Organic Frameworks (LnMOFs): A Versatile Platform towards Organomolecule Sensing. Coord. Chem. Rev. *470*, 214707. https://doi.org/10.1016/j.ccr.2022.214707.
9. Zhu, X., Zhang, H., and Zhang, F. (2023). Expanding NIR-II Lanthanide Toolboxes for Improved Biomedical Imaging and Detection. Acc. Mater. Res. *4*, 536–547. https://doi.org/10.1021/accountsmr.2c00117.
10. Kerbellec, N., Kustaryono, D., Haquin, V., Etienne, M., Daiguebonne, C., and Guillou, O. (2009). An Unprecedented Family of Lanthanide-Containing Coordination Polymers with Highly Tunable Emission Properties. Inorg. Chem. *48*, 2837–2843. https://doi.org/10.1021/ic801616y.
11. Vasile, R.L., Godoy, A.A., Puente Orench, I., Nemes, N.M., De La Peña O'Shea, V.A., Gutiérrez-Puebla, E., Martínez, J.L., Monge, M.Á., and Gándara, F. (2022). Influence of the Synthesis and Crystallization Processes on the Cation Distribution in a Series of Multivariate Rare-Earth Metal–Organic Frameworks and Their Magnetic Characterization. Chem. Mater. *34*, 7029–7041. https://doi.org/10.1021/acs.chemmater.2c01481.
12. Rodrigues, M.O., Dutra, J.D.L., Nunes, L.A.O., De Sá, G.F., De Azevedo, W.M., Silva, P., Paz, F.A.A., Freire, R.O., and A. Júnior, S. (2012). $Tb^{3+} \rightarrow Eu^{3+}$ Energy Transfer in Mixed-Lanthanide-Organic Frameworks. J. Phys. Chem. C *116*, 19951–19957. https://doi.org/10.1021/jp3054789.
13. Cadman, L.K., Mahon, M.F., and Burrows, A.D. (2018). The effect of metal distribution on the luminescence properties of mixed-lanthanide metal–organic frameworks. Dalton Trans. *47*, 2360–2367. https://doi.org/10.1039/C7DT04583B.
14. Huang, J., Tao, L., Wei, H., Huang, H., Zhang, Q., and Zhou, B. (2025). Full-color tuning in multi-layer core-shell nanoparticles from single-wavelength excitation. Nat. Commun. *16*, 2378. https://doi.org/10.1038/s41467-025-57622-y.
15. Lei, L., Wang, Y., Xu, W., Ye, R., Hua, Y., Deng, D., Chen, L., Prasad, P.N., and Xu, S. (2022). Manipulation of time-dependent multicolour evolution of X-ray excited afterglow in lanthanide-doped fluoride nanoparticles. Nat. Commun. *13*, 5739. https://doi.org/10.1038/s41467-022-33489-1.
16. Huang, D., Li, F., Ågren, H., and Chen, G. (2025). Inhibiting concentration quenching in Yb3+-Tm3+ upconversion nanoparticles by suppressing back energy transfer. Nat. Commun. *16*, 4218. https://doi.org/10.1038/s41467-025-59452-4.
17. Chen, L., Wang, H.-F., Li, C., and Xu, Q. (2020). Bimetallic metal–organic frameworks and their derivatives. Chem. Sci. *11*, 5369–5403. https://doi.org/10.1039/D0SC01432J.
18. Da Luz, L.L., Lucena Viana, B.F., Da Silva, G.C.O., Gatto, C.C., Fontes, A.M., Malta, M., Weber, I.T., Rodrigues, M.O., and Júnior, S.A. (2014). Controlling the energy transfer in lanthanide–organic frameworks for the production of white-light emitting materials. CrystEngComm *16*, 6914–6918. https://doi.org/10.1039/C4CE00538D.
19. Abdallah, A., Daiguebonne, C., Suffren, Y., Rojo, A., Demange, V., Bernot, K., Calvez, G., and Guillou, O. (2019). Microcrystalline Core–Shell Lanthanide-Based Coordination Polymers for Unprecedented Luminescent Properties. Inorg. Chem. *58*, 1317–1329. https://doi.org/10.1021/acs.inorgchem.8b02815.
20. Chen, D., Sedykh, A.E., Gomez, G.E., Neumeier, B.L., Santos, J.C.C., Gvilava, V., Maile, R., Feldmann, C., WÖll, C., Janiak, C., et al. (2020). SURMOF Devices Based on Heteroepitaxial Architectures with White-Light Emission and Luminescent Thermal-Dependent Performance. Adv. Mater. Interfaces *7*, 2000929. https://doi.org/10.1002/admi.202000929.
21. Ghazy, A., Lastusaari, M., and Karppinen, M. (2023). White-light emitting multi-lanthanide terephthalate thin films by atomic/molecular layer deposition. J. Mater. Chem. C *11*, 5331–5336. https://doi.org/10.1039/D3TC00464C.
22. Yang, J., Yu, G., Sessler, J.L., Shin, I., Gale, P.A., and Huang, F. (2021). Artificial transmembrane ion transporters as potential therapeutics. Chem *7*, 3256–3291. https://doi.org/10.1016/j.chempr.2021.10.028.
23. Norvaisa, K., Torres-Huerta, A., and Valkenier, H. (2024). Synthetic transporters for oxoanions. Curr. Opin. Chem. Biol. *83*, 102542. https://doi.org/10.1016/j.cbpa.2024.102542.
24. Torres-Huerta, A., Velásquez-Hernández, M.D.J., Tamarit-Amoros, E., Raschetti, M., Pinkas, D., Jurček, O., Pérez, J., and Valkenier, H. (2025). Spatiotemporal Control of the Formation of Luminescent Lanthanide Complexes in Liposome-Based Nanoreactors. Angew. Chem. Int. Ed. *45*, e202510471. https://doi.org/10.1002/anie.202510471.
25. Torres-Huerta, A., and Valkenier, H. (2024). The Role of the Organic Moiety in the Diffusion and Transport of Carboxylates into Liposomes. Molecules *29*, 5124. https://doi.org/10.3390/molecules29215124.
26. Reineke, T.M., Eddaoudi, M., Fehr, M., Kelley, D., and Yaghi, O.M. (1999). From Condensed Lanthanide Coordination Solids to Microporous Frameworks Having Accessible Metal Sites. J. Am. Chem. Soc. *121*, 1651–1657. https://doi.org/10.1021/ja983577d.
27. Alammar, T., Hlova, I.Z., Gupta, S., Biswas, A., Ma, T., Zhou, L., Balema, V., Pecharsky, V.K., and Mudring, A.-V. (2020). Mechanochemical synthesis, luminescent and magnetic properties of lanthanide benzene-1,4-dicarboxylate coordination polymers $(Ln_{0.5}\ Gd_{0.5}\ )_2\ (1,4\text{-BDC})_3\ (H_2\ O)_4$; Ln = Sm, Eu, Tb. New J. Chem. *44*, 1054–1062. https://doi.org/10.1039/C9NJ02583A.
28. Haquin, V., Etienne, M., Daiguebonne, C., Freslon, S., Calvez, G., Bernot, K., Le Pollès, L., Ashbrook, S.E., Mitchell, M.R., Bünzli, J., et al. (2013). Color and Brightness Tuning in Heteronuclear Lanthanide Terephthalate Coordination Polymers. Eur. J. Inorg. Chem. *2013*, 3464–3476. https://doi.org/10.1002/ejic.201300381.
29. Eliseeva, S.V., and Bünzli, J.-C.G. (2010). Lanthanide luminescence for functional materials and bio-sciences. Chem. Soc. Rev. *39*, 189–227. https://doi.org/10.1039/B905604C.
30. Qin, X., Xu, J., Wu, Y., and Liu, X. (2019). Energy-Transfer Editing in Lanthanide-Activated Upconversion Nanocrystals: A Toolbox for Emerging Applications. ACS Cent. Sci. *5*, 29–42. https://doi.org/10.1021/acscentsci.8b00827.
31. Cho, U., and Chen, J.K. (2020). Lanthanide-Based Optical Probes of Biological Systems. Cell Chem. Biol. *27*, 921–936. https://doi.org/10.1016/j.chembiol.2020.07.009.
32. Diamantis, S.A., Hatzidimitriou, A., Plessas, A.K., Pournara, A., Manos, M.J., Papaefstathiou, G.S., and Lazarides, T. (2020). Alkaline earth-organic frameworks with amino derivatives of 2,6-naphthalene dicarboxylates: structural studies and fluorescence properties. Dalton Trans. *49*, 16736–16744. https://doi.org/10.1039/D0DT03325A.
33. Chen, Y., Wang, N., Lv, Y., Zhou, C., Liang, Q., and Su, X. (2024). Construction of the fluorescence sensing platform with a bifunctional Cu@MOF nanozyme for determination of alkaline phosphatase and its inhibitor. Talanta *278*, 126564. https://doi.org/10.1016/j.talanta.2024.126564.
34. Xia, T., Cao, W., Cui, Y., Yang, Y., and Qian, G. (2021). Water-sensitive multicolor luminescence in lanthanide-organic framework for anti-counterfeiting. Opto-Electron. Adv. *4*, 200063. https://doi.org/10.29026/oea.2021.200063.

35. Dang, J., Zhu, R., Fang, W., Hu, Y., Wu, Y., Xin, S., Li, M., Chen, B., Zhao, H., and Li, Z. (2022). The construction of a ratiometric dual-emitting amorphous europium-organic frameworks for sensitive detection of water in white spirit. Dyes Pigm. *206*, 110602. https://doi.org/10.1016/j.dyepig.2022.110602.

36. Brouwer, A.M. (2011). Standards for photoluminescence quantum yield measurements in solution (IUPAC Technical Report). Pure Appl. Chem. *83*, 2213–2228. https://doi.org/10.1351/PAC-REP-10-09-31.

37. Briois, V., Fonda, E., Belin, S., Barthe, L., La Fontaine, C., Langlois, F., Ribbens, M., and Villain, F. (2011). SAMBA: The 4–40 keV X-ray absorption spectroscopy beamline at SOLEIL. In UVX 2010 - 10e Colloque sur les Sources Cohérentes et Incohérentes UV, VUV et X ; Applications et Développements Récents, A. Calisti, C. Mossé, and S. Ferri, eds. (EDP Sciences), pp. 41–47. https://doi.org/10.1051/uvx/2011006.

38. Landrot, G., and Fonda, E. (2025). *Fastosh*: a software for the treatment of XAFS datasets of environmental relevance or acquired in *operando* conditions. J. Synchrotron Radiat. *32*, 1085–1094. https://doi.org/10.1107/S1600577525003923.

39. Newville, M. (2013). Larch: An Analysis Package for XAFS and Related Spectroscopies. J. Phys, Conf. Ser. *430*, 012007. https://doi.org/10.1088/1742-6596/430/1/012007.

40. Rehr, J.J., Kas, J.J., Prange, M.P., Sorini, A.P., Takimoto, Y., and Vila, F. (2008). Ab initio theory and calculations of X-ray spectra. Comptes Rendus Phys *10*, 548–559. https://doi.org/10.1016/j.crhy.2008.08.004.

41. Chong, S., Riley, B.J., and Nelson, Z.J. (2020). Dehydration synthesis and crystal structure of terbium oxychloride, TbOCl. Acta Crystallogr. E Crystallogr. Commun. *76*, 621–624. https://doi.org/10.1107/S2056989020004387.

42. Martinez-Gomez, N.C., Vu, H.N., and Skovran, E. (2016). Lanthanide Chemistry: From Coordination in Chemical Complexes Shaping Our Technology to Coordination in Enzymes Shaping Bacterial Metabolism. Inorg. Chem. *55*, 10083–10089. https://doi.org/10.1021/acs.inorgchem.6b00919.

43. Boglaienko, D., Andersen, A., Heald, S.M., Varga, T., Mortensen, D.R., Tetef, S., Seidler, G.T., Govind, N., and Levitskaia, T.G. (2022). X-ray absorption spectroscopy of trivalent Eu, Gd, Tb, and Dy chlorides and oxychlorides. J. Alloys Compd. *897*, 162629. https://doi.org/10.1016/j.jallcom.2021.162629.

Cell Reports Physical Science, Volume *7*

Supplemental information

Color-tunable luminescent $Tb_xEu_y(BDC)$ complexes assembled within liposome-based nanoreactors

Aaron Torres-Huerta, Miriam de J. Velásquez-Hernández, Sven Lempereur, Ludovic Troian-Gautier, Giulia Veronesi, and Hennie Valkenier

SUPPLEMENTAL METHODS

1. Chloride Ion-Selective Electrode (Cl-ISE)

Chloride concentrations outside and inside the liposomes were quantified using a Fisherbrand Accument AB250 chloride ion-selective electrode. The electrode was calibrated prior to each measurement using $NaNO_3$ (0.5 M) solutions containing NaCl at concentrations of 1 ppm, 10 ppm, 100 ppm, and 1000 ppm.
For each measurement, 3 mL of the $LnCl_3$@LP suspension (3 mM lipid) was transferred into a 25 mL beaker and stirred gently. The Cl-ISE was immersed into the sample, and the reading was recorded once it stabilized, corresponding to the external chloride concentration $[Cl^-]_{ext}$. To determine the total chloride content, 100 µL of Triton X-100 (5% w/w in water) was added to lyse the liposomes, and the measurement taken after complete lysis was recorded as the global chloride concentration $[Cl^-]_{global}$. See Figure S1.

2. Scanning Electron Microscopy and Energy-Dispersive X-ray Spectroscopy (SEM-EDX)

SEM-EDX analyses were conducted using an APREO S Thermo Fisher scanning electron microscope equipped with a Trinity detector system along an A-tube biased at 8 kV. For sample preparation, a small drop of the liposome suspension was deposited onto a silicon chip and dried under vacuum. To remove residual Na_2SO_4, the dried samples were gently rinsed with milli-Q water and then dried again under vacuum. To ensure proper surface conductivity during imaging, the samples were coated with a thin carbon layer (<5 nm) using a vacuum sputter coater. EDX spectra were acquired from at least three different vesicles to confirm elemental composition and ensure representativity. See Figure S4.

3. Quantum yield determinations

UV-vis absorption spectra were recorded on an Agilent Cary 60 spectrophotometer in a quartz cuvette with a 1 cm path length.

Photoluminescence measurements for quantum yield determinations were recorded on an Edinburgh Instruments FS5 Spectrofluorometer. The steady-state photoluminescence spectra were recorded using a 150 W Xenon arc lamp as the excitation source. The photoluminescence was detected at a right angle to the excitation beam using a single photon counting PMT-900 with PMT-EXT, allowing detection up to 980 nm in a temperature stabilized housing.

The measurements were performed four times, using water for the complexes and 0.05 M H_2SO_4 for the quinine reference.[2] Emission spectra were recorded under identical instrumental settings for all measurements. The quantum yield was calculated by integrating the emission spectra over the range 360–750 nm, with correction for the absorbance at the excitation wavelength (250 nm), using the following equation:

$$\emptyset_S = \emptyset_R \left(\frac{PL_S}{PL_R}\right)\left(\frac{1-10^{-A_R}}{1-10^{-A_S}}\right)\left(\frac{\eta_S}{\eta_R}\right)^2$$

(S) Sample; (R) Reference = quinine
∅ : Emission Quantum Yield; $\emptyset_R = 0.52$

PL : Photoluminescence intensity
A : Absorbance at the excitation λ
η: Refractive index of the solution solvent η_S = 0.337; η_R= 0.333

4. X-ray Absorption Spectroscopy (XAS)

Experimental Tb L_{III}-edge X ray Absorption Spectroscopy (XAS) data were acquired on the beamline SAMBA at the SOLEIL synchrotron, Saint-Aubin, France.[3] The energy region 7.35-8.20 keV with a Si(220) double-crystal monochromator equipped with sagittal focusing. Drops of solution samples were frozen in liquid nitrogen (LN2) and measured in a He cryostat. The spectra were acquired in fluorescence mode, using a 35-elements monolithic planar Ge pixel array detector.
Liposomal suspensions were concentrated to achieve a final lipid concentration of 23 mM. 5 to 20 spectra were acquired and averaged using the Fastosh software.[4] Further data reduction and analysis was performed with the Larix GUI provided within the Larch data analysis suite.[5] EXAFS spectra were extracted and Fourier-transformed in the k range [2.6, 10] $Å^{-1}$, with a k-weight of 3, then fitted in the real space in the region [1.5 – 3.8] Å. Theoretical amplitudes and phase shifts were generated with the FEFF8.0 code,[6] including self-consistent calculations, using Tb oxychloride (ICSD 120251) as the input structure.[7] Two or three single-scattering paths were necessary to reproduce the experimental spectra, corresponding to atomic shells populated by O, Cl, and Tb around the Tb absorber. For each atomic shell, the free fit parameters were the number of atoms (N), interatomic distances (R), and Debye-Waller factors (σ^2). A common shift in the energy origin (ΔE_0) was assigned to all scattering paths and allowed to vary. See Figure S29 and S30 and Table S1.

5. FT-IR spectroscopy

To further confirm the lanthanide-dicarboxylate coordination, FT-IR spectroscopy was performed. For these measurements, the external sulphate solution was replaced with a 21 mM NaCl solution (pH 7) to avoid interference from SO_4^{2-} absorption bands. Furthermore, a second dialysis step with 21 mM NaCl solution was performed after the Tb_xEu_yBDC@LP formation to remove any remaining exterior carboxylate salts. See Figure S31 and S32.

SUPPLEMENTAL NOTES

NOTE S1. Formation of bilamellar vesicles

The unilamellar-to-bilamellar transition may be attributed to osmotic pressure variations between the bulk solution and the internal liposomal cavity during TbBDC@LP formation. This osmotic shrinkage is likely induced by two key processes: (*i*) the exchange of one BDC^{2-} anions for two Cl^{-} ions via an antiport mechanism to avoid the buildup of an electrostatic potential and (*ii*) the coordination of the BDC^{2-} with Tb^{3+} cations inside the liposome (Figure S2a). The combination of these processes induces osmotic stress, driving the inward folding of the lipid bilayer and the subsequent formation of bilamellar vesicles (Figure S2b).[1]

NOTE S2. Emission studies in RGB systems

NH_2BDC^{2-} exhibits fluorescence between 400 and 550 nm when excited at 285 nm (Figure S19). We note that the 450 nm long-pass filter (Figure S20), required to suppress excitation scattering and overtones that would be visible at 570 nm, partially attenuated the blue edge of the NH_2BDC^{2-} emission band (Figure S19b). However, the characteristic Tb^{3+} (542 nm) and Eu^{3+} (614 nm) emission bands are hardly affected.

NOTE S3. X-ray Absorption Spectroscopy (XAS)

We investigated the formation of Tb-BDC complex with X-ray Absorption Spectroscopy (XAS). To do so, we measured the Tb L_{III}-edge XAS spectra of three solution samples: the $TbCl_3$ salt, $TbCl_3$ encapsulated in liposomes ($TbCl_3$@LP), and $TbCl_3$ co-encapsulated in liposomes with BDC (TbBDC@LP). The X-ray Absorption Near Edge Structure (XANES) region of the X-ray absorption coefficient of the three samples is reported in Figure S29A. The spectra show minimum variations, indicative of a nearly identical coordination sphere, comprising the nature and number of the first neighbors, and their geometry. This is consistent with a same coordination sphere in all samples, composed by O atoms from water or carboxylates, which is favored in lanthanides.[8]

In contrast, the Fourier Transformed Extended X-ray Absorption Fine Structure (FT-EXAFS, Figure S29B) spectra reveal subtle but significant differences between the three samples. In particular, the spectrum of the $TbCl_3$ salt differs from those of $TbCl_3$@LP and TbBDC@LP, whereas the latter two nearly overlap in the region 1.5 - 4 Å. All samples display a first-shell peak centered at ~ 2 Å (not phase-corrected), the intensity of which is higher in the $TbCl_3$ salt. This observation is consistent with the similarity of XANES spectra, the latter region being more sensitive to the first neighbors of Tb^{3+} than to further atomic shells.
The fit of the FT-EXAFS spectra based on a structural model comprising O first neighbors, as well as Cl and Tb atoms, confirms that in all samples Tb^{3+} binds O atoms at a distance of 2.38-2.39 Å. The quantitative fit results are reported in Table S1. The average number of O atoms is slightly higher in $TbCl_3$ in solution than in $TbCl_3$ encapsulated into liposomes (6.4±0.2 *vs* 5.9±0.2), regardless of the presence of BDC.

In $TbCl_3$ in solution, an average number of 1.3 ± 0.4 Cl atoms surrounds the Tb absorber, with a Tb-Cl distance of 3.25 Å. This distance is longer than one observed in the literature for a $TbCl_3{\cdot}6H_2O$ solid powder (reported in Table S1 for comparison), consistently with the differences expected in solution *vs* solid samples. Accordingly, a higher disorder (indicated by an increase in the dynamical parameter σ^2, see Table S1) is observed in the $TbCl_3$ solution

with respect to the $TbCl_3·6H_2O$ powder. When $TbCl_3$ is encapsulated into liposomes, the EXAFS analysis exposes the formation of a Tb cluster, indicated by the presence of 6.0 ± 1.7 Tb atoms surrounding the Tb absorber, with an average Tb-Tb distance of 3.34 ± 0.02 Å (Table S1). When $TbCl_3$ and BDO are co-encapsulated, these values are unchanged within the error, as well as the number of O neighbors and the Tb-O distance.
It is worth to mention that both Cl and Tb neighbors are necessary to fit the features in the 2.8-3.8 Å region of the FT-EXAFS spectra in the $TbCl_3$@LP and TbBDC@LP samples (Figure S29B). The interplay between these two contributions is particularly clear in the real part of the FT EXAFS signal (Figure S30, dark yellow *vs* magenta curves), where they cancel out in the range 2.0-3.0 Å, and they sum up to account for the spectral features in the 3.0-3.8 Å region.

Overall, these results indicate that the confinement into liposomes fosters the formation of Tb clusters, in which Tb^{3+} binds oxygen atoms from the solution of from the phosphate heads of the membrane. When BDC is co-encapsulated, the carboxylate moieties can replace O atoms in the pre-formed Tb-cluster, inducing the formation of the Tb-BDC complex and the acquisition of luminescence, without changing the nature of the Tb^{3+} ligands.

NOTE S4. FT-IR spectroscopy

Six representative samples were analysed: $Tb_{100}Eu_{00}$BDC@LP, $Tb_{00}Eu_{100}$BDC@LP, $Tb_{50}Eu_{50}$BDC@LP, $Tb_{100}Eu_{00}NH_2$BDC@LP, $Tb_{00}Eu_{100}NH_2$DC@LP and $Tb_{50}Eu_{50}NH_2$BDC@LP. These spectra were compared with those obtained from control samples, including $LnCl_3$@LP and $LnCl_3$@LP + **T1**, as well as the corresponding free dicarboxylate ligands (BDC^{2-} or NH_2BDC^{2-}). In dried samples of BDC-based systems ($Tb_{100}Eu_{00}$BDC@LP, $Tb_{00}Eu_{100}$BDC@LP, and $Tb_{50}Eu_{50}$BDC@LP), the asymmetric $RCOO^-$ stretching vibration appeared at 1562 cm^{-1}, which is shifted compared to the uncoordinated BDC^{2-} ligand (1551 cm^{-1}). Similarly, for NH_2BDC-based systems ($Tb_{100}Eu_{00}NH_2$BDC@LP, $Tb_{00}Eu_{100}NH_2$DC@LP, and $Tb_{50}Eu_{50}NH_2$BDC@LP) the asymmetric $RCOO^-$ band was observed in the range 1566-1570 cm^{-1}, compared to 1557 cm^{-1} for the free NH_2BDC^{2-} ligand.

SUPPLEMENTAL FIGURES

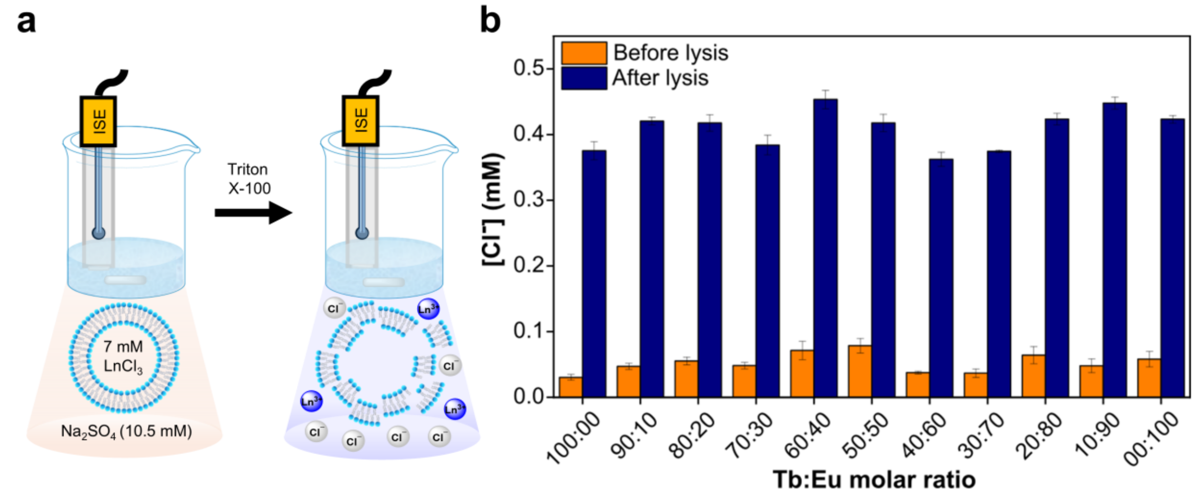

Figure S1: Quantification of encapsulated lanthanide chloride content. A) Schematic of Cl-ISE assay to determine Cl^- concentration before and after lysing $Tb_xEu_yCl_3$@LP systems. B) Chloride ion-selective electrode (Cl-ISE) measurements of $Tb_xEu_yCl_3$@LP suspensions. Error bars represent the standard deviation of three independent measurements.

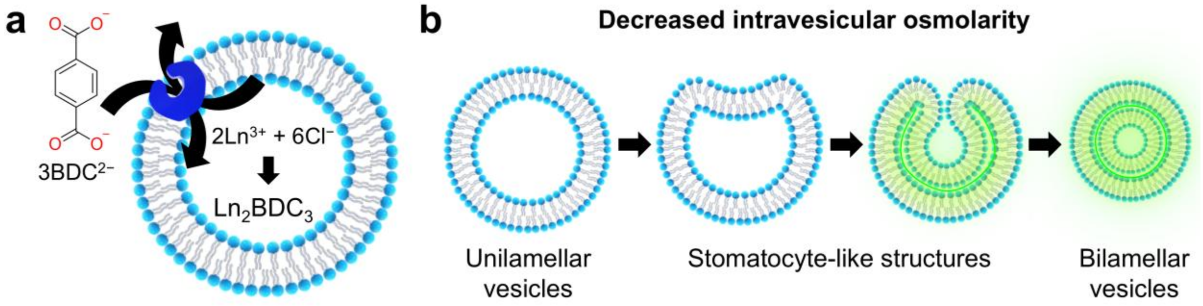

Figure S2: Schematic illustration of bilamellar vesicle formation. A), B) Schematic representation of bilamellar vesicle formation induced by osmolarity reduction upon the anti-port BDC^{2-} transport.

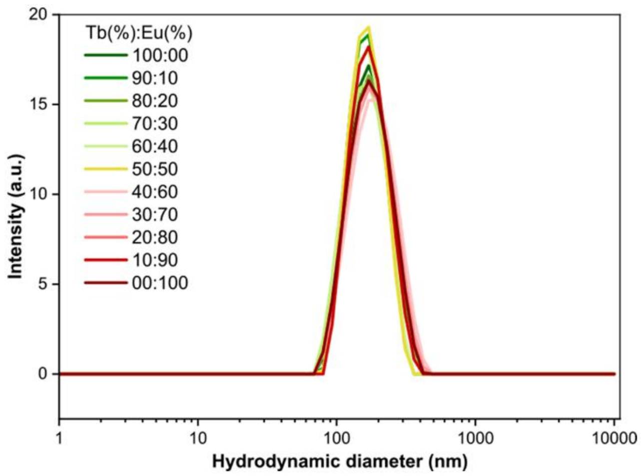

Figure S3: Full-range DLS intensity. Distributions for representative Tb_xEu_yBDC@LP systems.

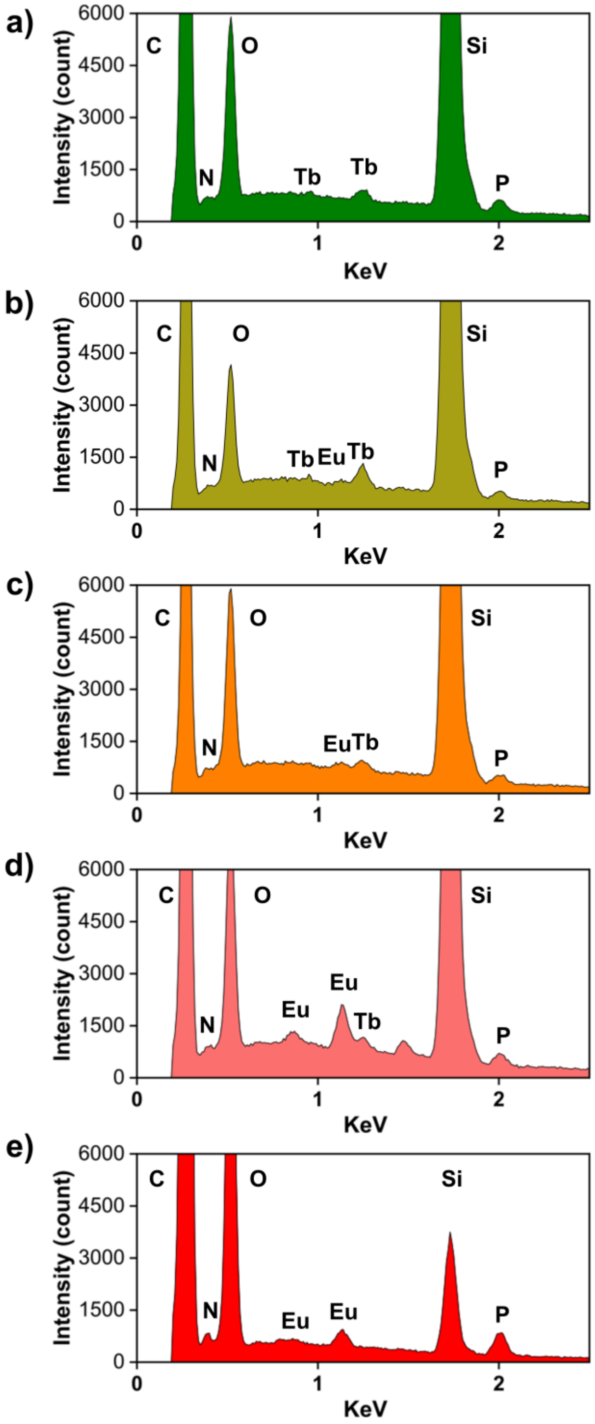

Figure S4: Energy-dispersive X-ray spectroscopy (EDX) elemental analysis. EDX elemental analysis of selected Tb_xEu_yBDC@LP samples: A) $Tb_{100}Eu_{00}$BDC@LP, B) $Tb_{80}Eu_{20}$BDC@LP, C) $Tb_{50}Eu_{50}$BDC@LP, D) $Tb_{20}Eu_{80}$BDC@LP, and E) $Tb_{00}Eu_{100}$BDC@LP.

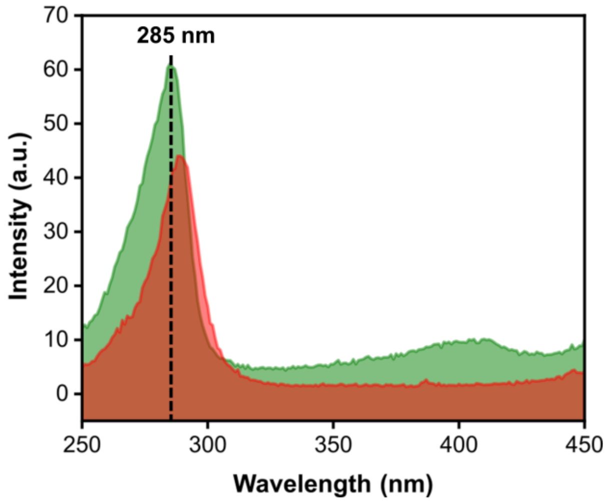

Figure S5: Excitation spectra in Tb and Eu reference systems. Excitation spectra of $Tb_{100}Eu_{00}$BDC@LP (λ_{em} = 542) and $Tb_{00}Eu_{100}$BDC@LP (λ_{em} = 614).

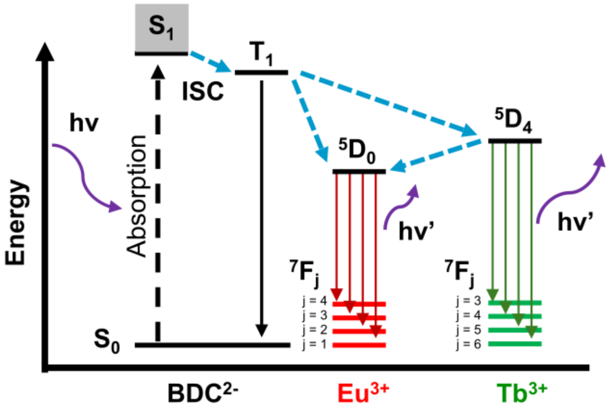

Figure S6: Schematic representation of the energy transfer pathways in Tb_xEu_yBDC@LP systems. Excitation of the BDC^{2-} ligand results in energy transfer to the $^5D_4$ excited state of Tb^{3+} and the $^5D_0$ excited state of Eu^{3+}. In mixed-metal systems, additional intermetallic energy transfer occurs from Tb^{3+} to Eu^{3+}.

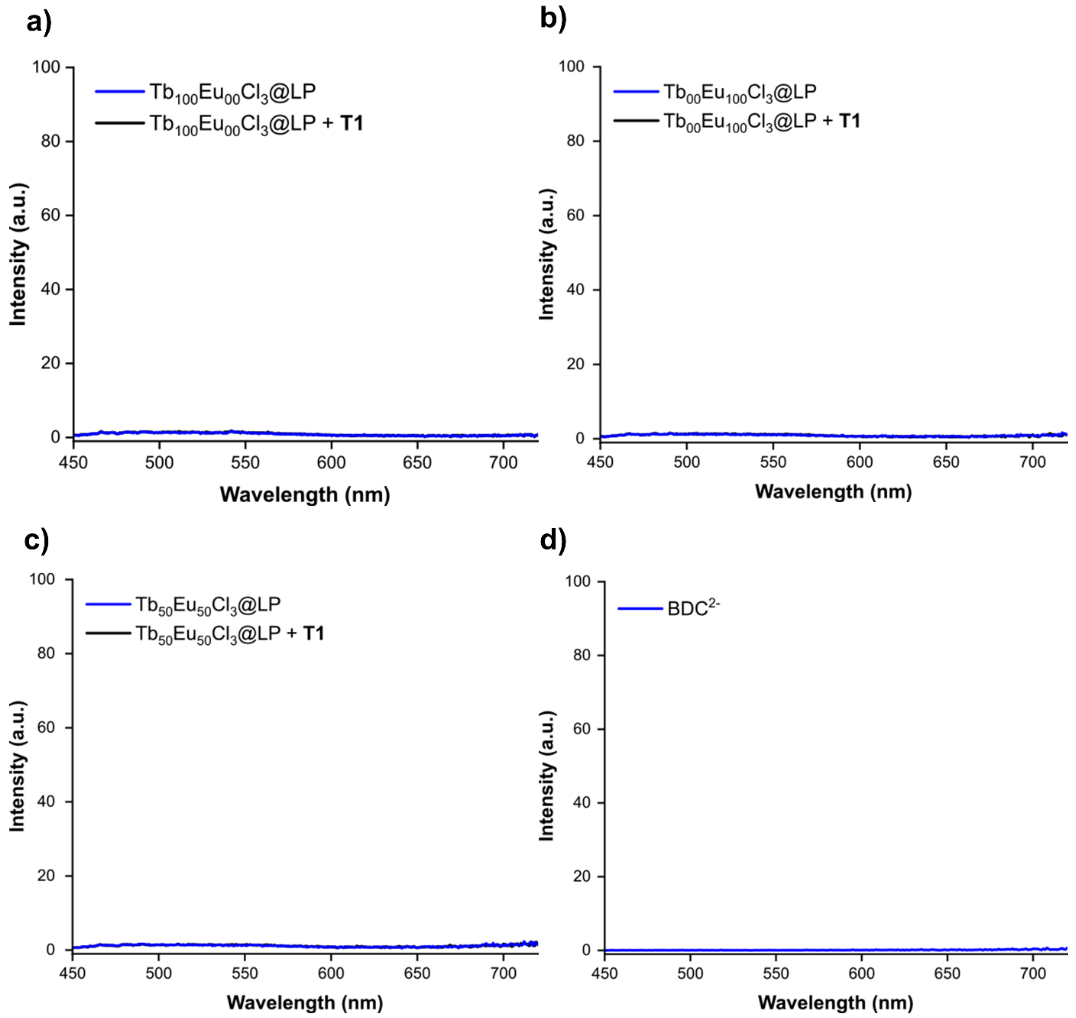

Figure S7: Control emission spectra. Emission spectra recorded under 285 nm excitation (using a 450 nm long-pass filter) for 3 mL liposome samples in the absence and presence of the anion transporter **T1**: A) $Tb_{100}Eu_{00}Cl_3$@LP and $Tb_{100}Eu_{00}Cl_3$@LP + **T1**; B) $Tb_{00}Eu_{100}Cl_3$@LP and $Tb_{00}Eu_{100}Cl_3$@LP + **T1**; C) $Tb_{50}Eu_{50}Cl_3$@LP and $Tb_{50}Eu_{50}Cl_3$@LP + **T1**; and D) 1 mM BDC^{2-} in 3 mL of sulphate solution.

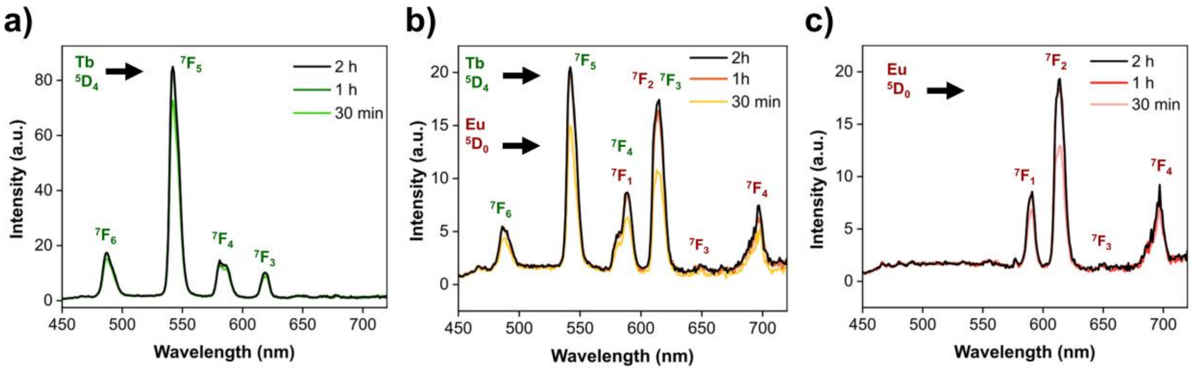

Figure S8: Stability of Tb_xEu_yBDC@LP systems over time. Emission spectra recorded at 30 minutes, 1 hour and 2 hours for selected Tb_xEu_yBDC@LP samples: A) $Tb_{100}Eu_{00}$BDC@LP, B) $Tb_{50}Eu_{50}$BDC@LP, and C) $Tb_{00}Eu_{100}$BDC@LP (λ_{ex}= 285 nm, using a 450 nm long-pass filter). All samples exhibit negligible changes in emission intensity after 1 hour.

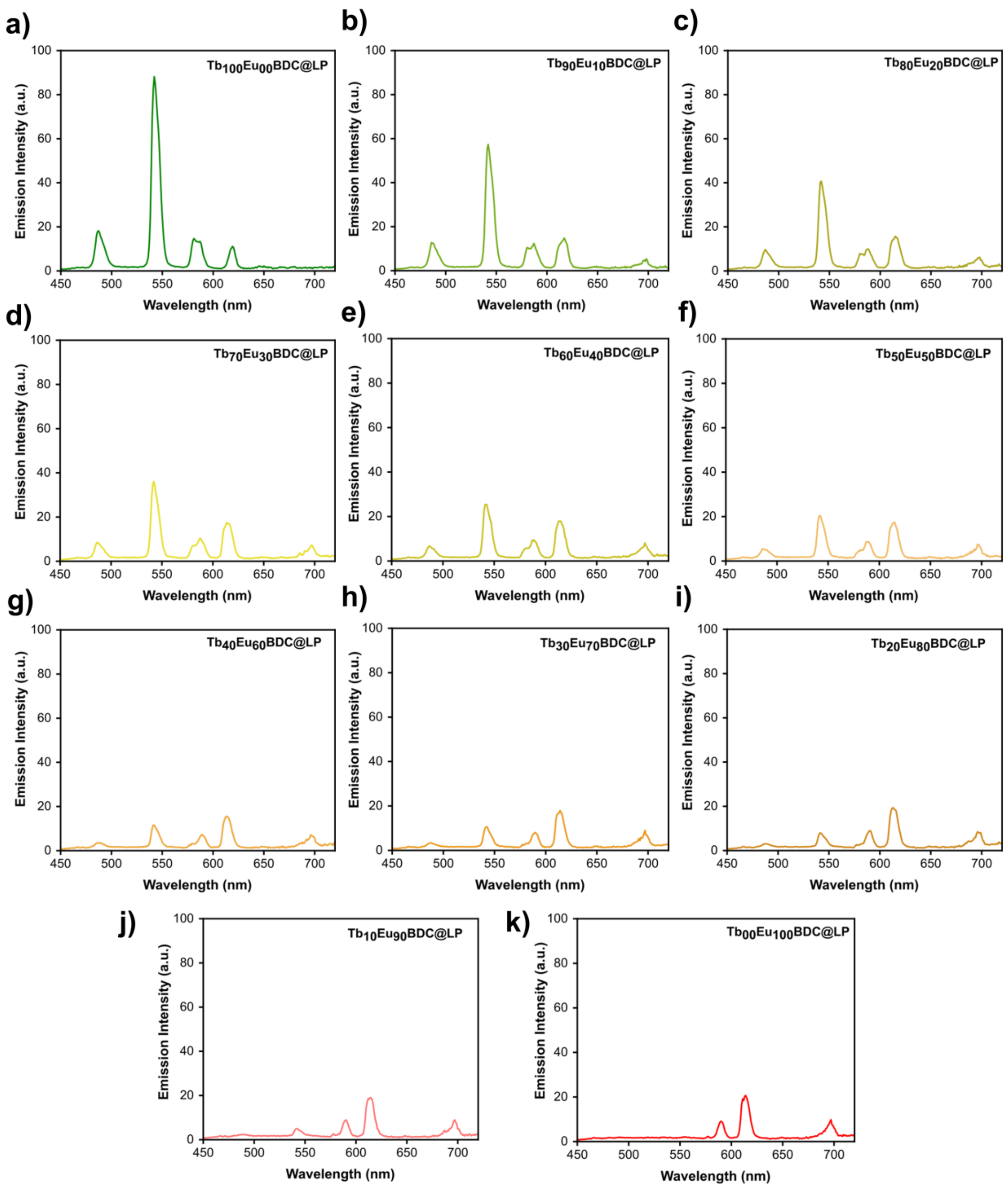

Figure S9: Emission spectra of Tb_xEu_yBDC@LP samples with varying Tb:Eu molar ratios. The spectra were recorded after complexation, and no further changes in emission intensity were observed upon excitation at 285 nm, using a 450nm long-pass filter.

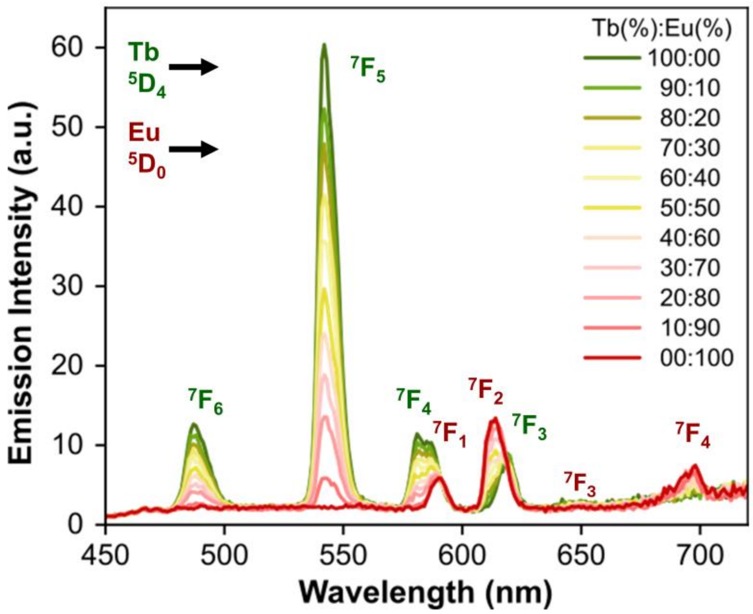

Figure S10: Emission spectra of physically mixed lanthanide systems without intermetallic interaction. Emission spectra for physically mixed samples composed of $Tb_{100}Eu_{00}$BDC@LP and $Tb_{00}Eu_{100}$BDC@LP in varying Tb:Eu molar ratios (λ_{ex}= 285 nm, using a 450nm long-pass filter).

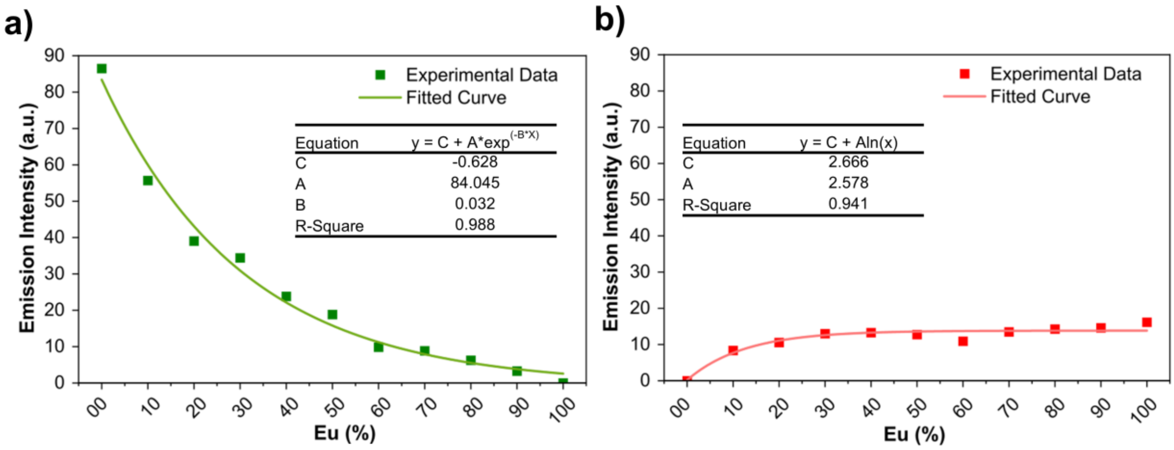

Figure S11: Non-linear variation of Tb and Eu emission intensities in multivariate systems. Fitting of the emission intensities at 542 nm and 614 nm as a function of Eu^{3+} mole fraction in Tb_xEu_yBDC@LP samples.

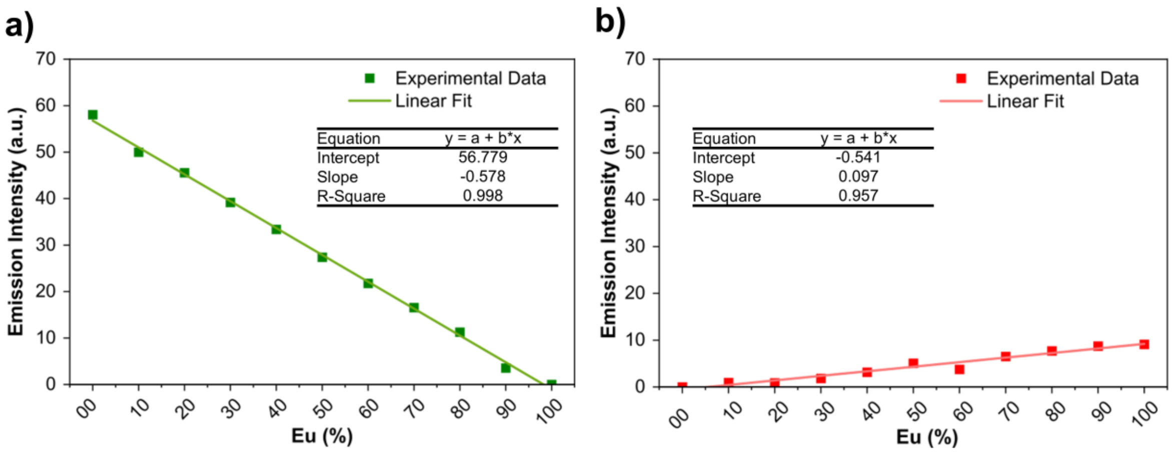

Figure S12: Linear variation of Tb and Eu emission intensities in physical mixtures. Linear fitting of the emission intensities at 542 nm and 614 nm as a function of Eu^{3+} mole fraction in physically mixed samples composed of $Tb_{100}Eu_{00}$BDC@LP and $Tb_{00}Eu_{100}$BDC@LP. The linear trends confirm the independent luminescence contributions of physically separated lanthanide complexes, in contrast to the cooperative behaviour observed in co-encapsulated systems.

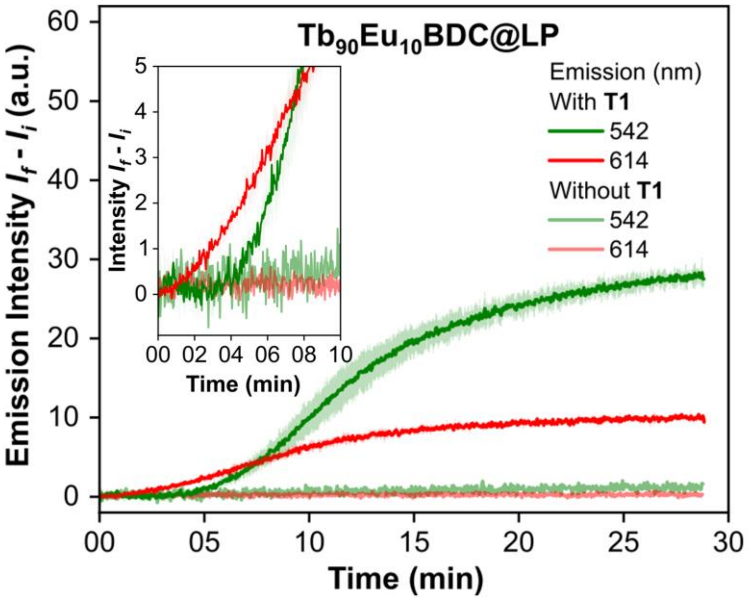

Figure S13: Kinetic emission data for $Tb_{90}Eu_{10}$BDC@LP. Emission intensities at 542 nm (green line) correspond to the Tb^{3+} $^5D_4 \rightarrow {}^7F_5$ transition, while those at 614 nm (red line) correspond to the Eu^{3+} $^5D_0 \rightarrow {}^7F_2$ transition. Control experiments without transporter **T1** are shown in lighter green and red lines. Shaded areas represent the standard deviation of three independent measurements.

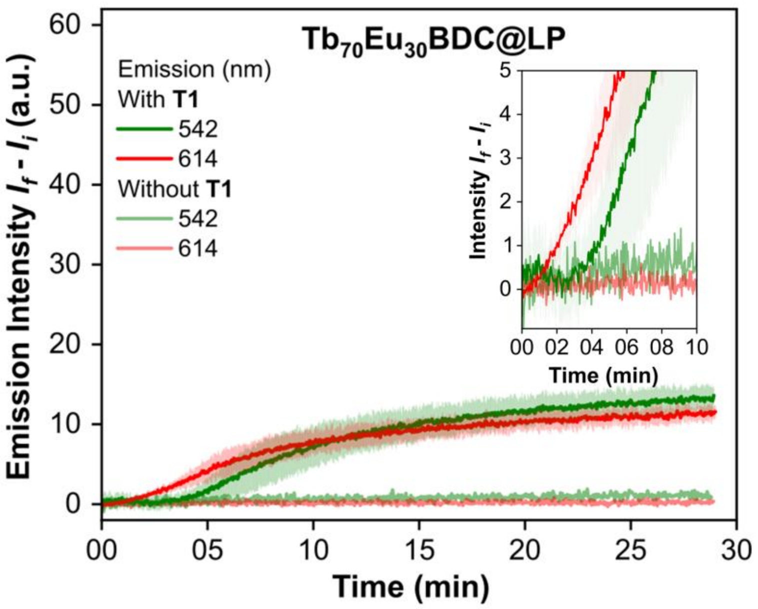

Figure S14: Kinetic emission data for $Tb_{70}Eu_{30}$BDC@LP. Emission intensities at 542 nm (green line) correspond to the Tb^{3+} $^5D_4\rightarrow{}^7F_5$ transition, while those at 614 nm (red line) correspond to the Eu^{3+} $^5D_0\rightarrow{}^7F_2$ transition. Control experiments without transporter **T1** are shown in lighter green and red lines. Shaded areas represent the standard deviation of three independent measurements.

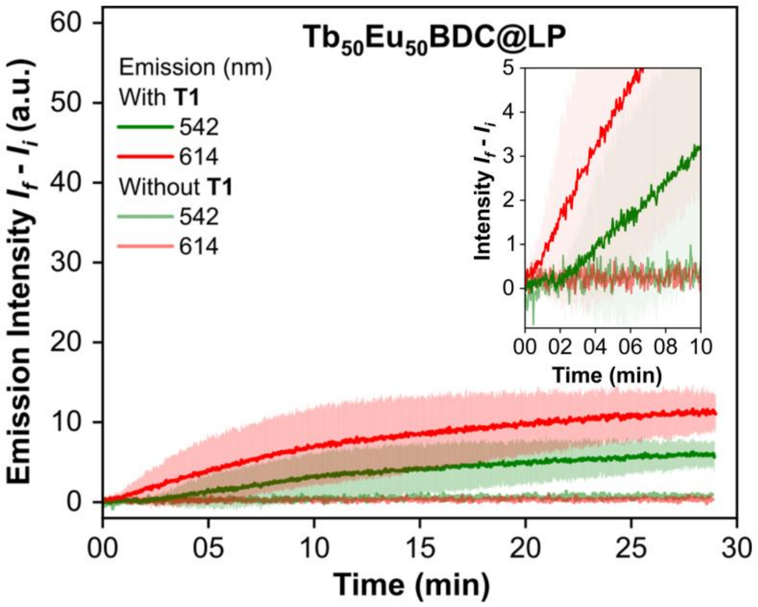

Figure S15: Kinetic emission data for $Tb_{50}Eu_{50}$BDC@LP. Emission intensities at 542 nm (green line) correspond to the Tb^{3+} $^5D_4\rightarrow{}^7F_5$ transition, while those at 614 nm (red line) correspond to the Eu^{3+} $^5D_0\rightarrow{}^7F_2$ transition. Control experiments without transporter **T1** are shown in lighter green and red lines. Shaded areas represent the standard deviation of three independent measurements.

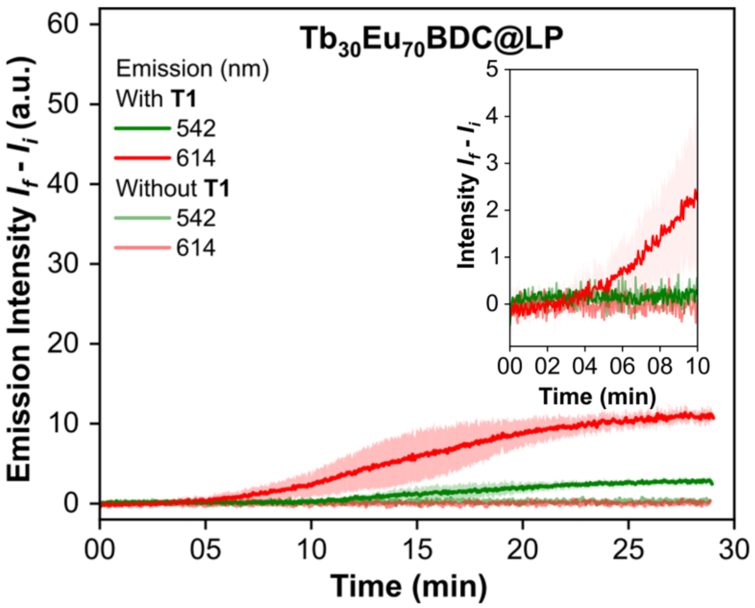

Figure S16: Kinetic emission data for $Tb_{30}Eu_{70}$BDC@LP. Emission intensities at 542 nm (green line) correspond to the Tb^{3+} $^5D_4 \rightarrow ^7F_5$ transition, while those at 614 nm (red line) correspond to the Eu^{3+} $^5D_0 \rightarrow ^7F_2$ transition. Control experiments without transporter **T1** are shown in lighter green and red lines. Shaded areas represent the standard deviation of three independent measurements.

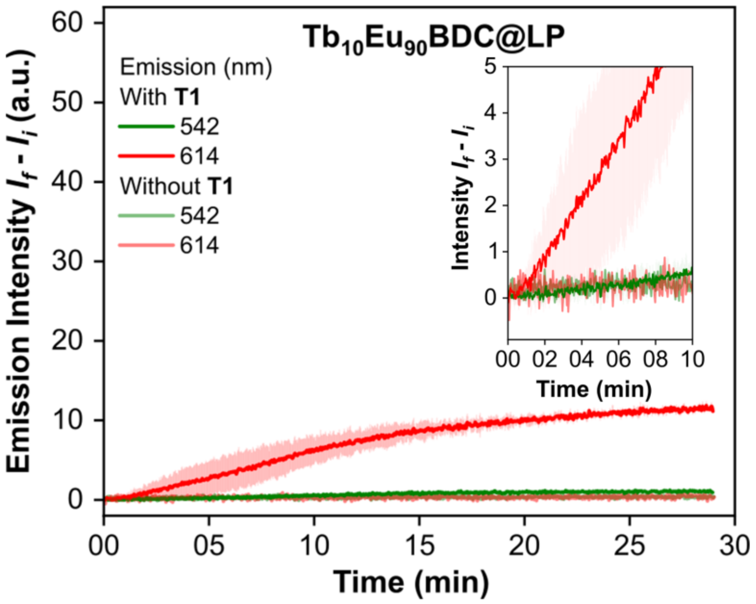

Figure S17: Kinetic emission data for $Tb_{10}Eu_{90}$BDC@LP. Emission intensities at 542 nm (green line) correspond to the Tb^{3+} $^5D_4 \rightarrow ^7F_5$ transition, while those at 614 nm (red line) correspond to the Eu^{3+} $^5D_0 \rightarrow ^7F_2$ transition. Control experiments without transporter **T1** are shown in lighter green and red lines. Shaded areas represent the standard deviation of three independent measurements.

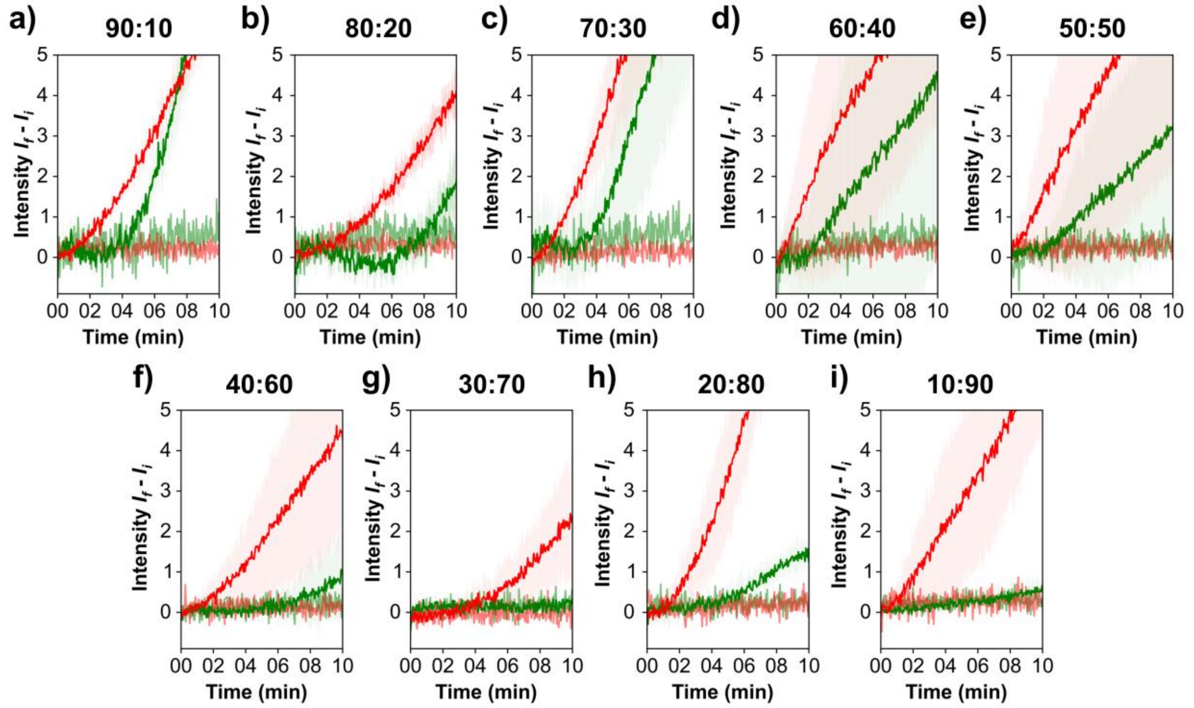

Figure S18: Real-time emission kinetics during early-stage coordination. Real-time emission monitoring of the coordination between BDC and lanthanide ions within liposome-based nanoreactors during the first 10 minutes following reactant addition. Emission intensities at 542 nm (green line) correspond to the Tb^{3+} $^5D_4 \rightarrow ^7F_5$ transition, while those at 614 nm (red line) correspond to the Eu^{3+} $^5D_0 \rightarrow ^7F_2$ transition. Kinetic data are shown for: A) $Tb_{90}Eu_{10}$BDC@LP, B) $Tb_{80}Eu_{20}$BDC@LP, C) $Tb_{70}Eu_{30}$BDC@LP, D) $Tb_{60}Eu_{40}$BDC@LP, E) $Tb_{50}Eu_{50}$BDC@LP, F) $Tb_{40}Eu_{60}$BDC@LP, G) $Tb_{30}Eu_{70}$BDC@LP, H) $Tb_{20}Eu_{80}$BDC@LP, and I) $Tb_{10}Eu_{90}$BDC@LP. Control experiments without transporter **T1** are represented with a slight green and red colour. Shaded areas represent the standard deviation of three independent measurements.

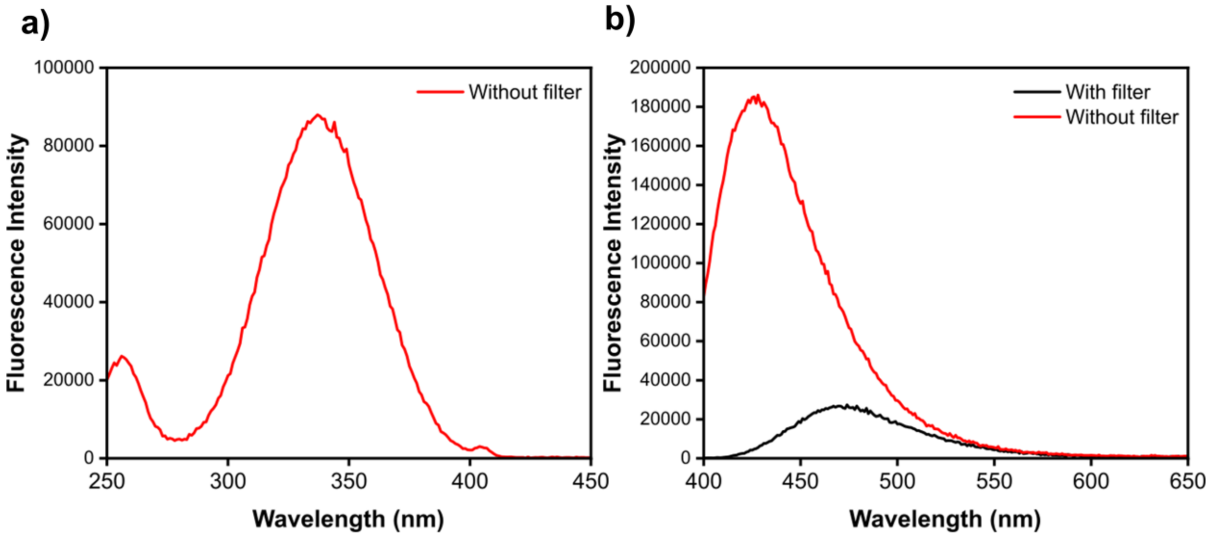

Figure S19. Long-pass filter effect on excitation and emission spectra of NH_2BDC^{2-} ligand. Fluorescence spectra of NH_2BDC^{2-} ligand (1.6 μM in 3 mL of 10.5 mM Na_2SO_4) obtained in the presence and absence of the 450 nm long-pass filter. A) The excitation spectrum was obtained when recording the emission at 470 nm, and B) the emission spectra upon excitation at 285 nm.

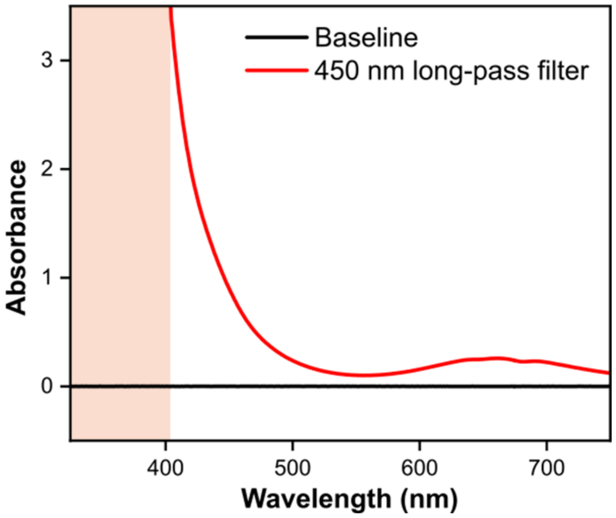

Figure S20: Absorbance profile of the 450 nm long-pass filter used in emission measurements. Spectrum of the 450 nm long-pass filter from 400-750 nm upon excitation at 285 nm.

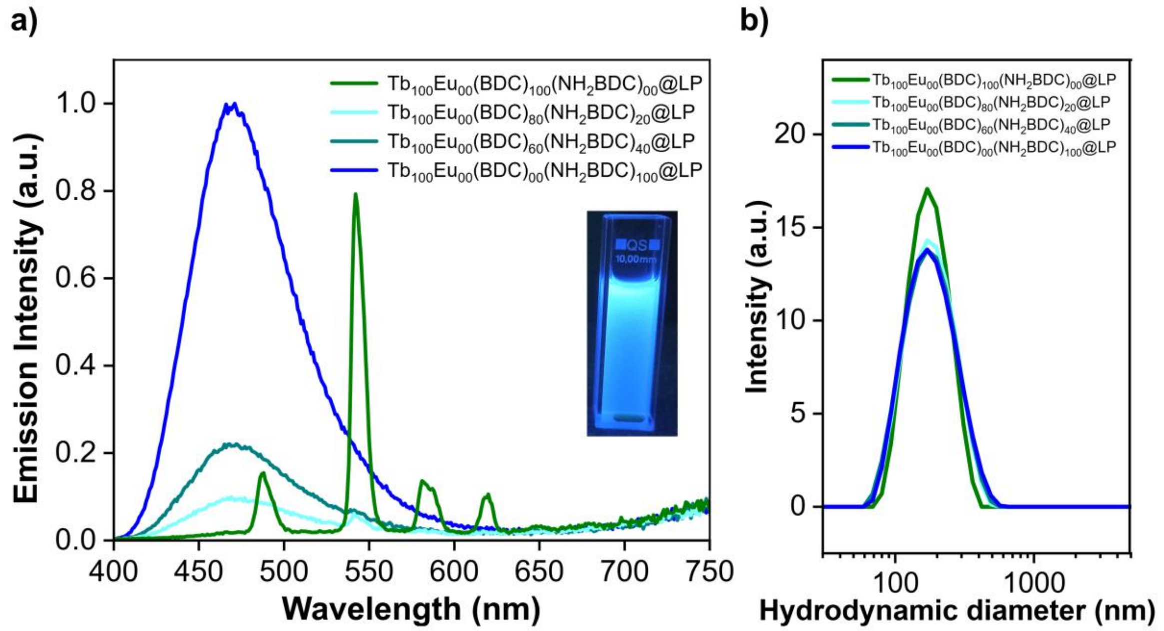

Figure S21: Mixed-ligand systems. A) Emission spectra of mixed-ligand systems prepared with varying BDC^{2-}/NH_2BDC^{2-} molar ratios, showing the evolution from Tb^{3+} emission (λ_{ex}= 285 nm, using a 450nm long-pass filter). Inset: Photograph of the $Tb_{100}Eu_{00}(BDC)_{00}(NH_2BDC)_{100}$@LP sample under 254 nm UV illumination. B) corresponding DLS analysis.

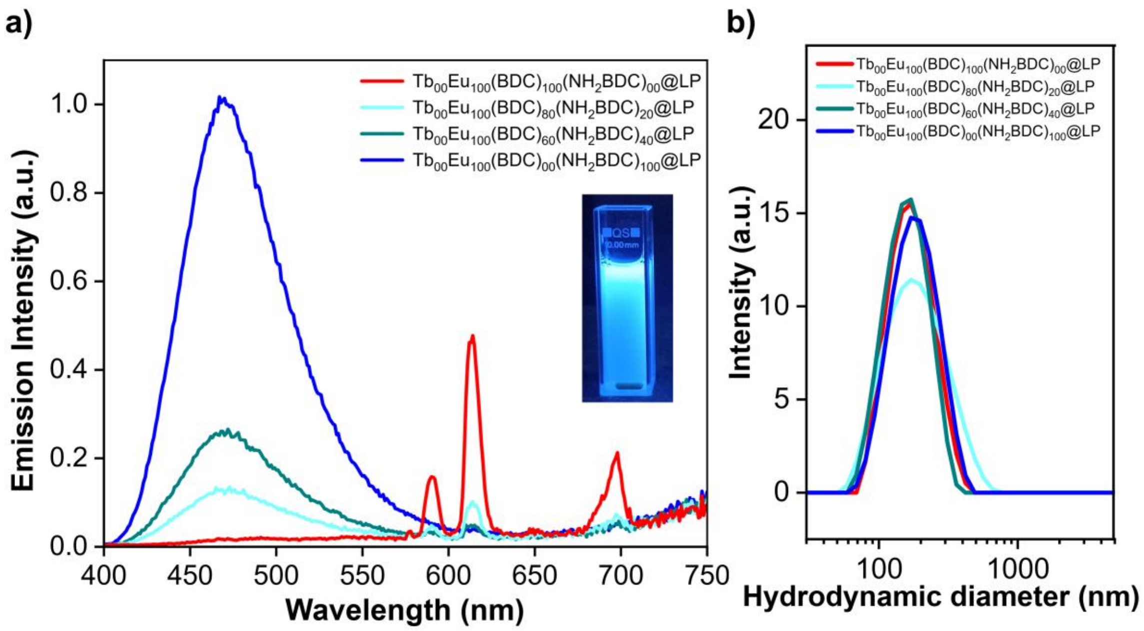

Figure S22: Mixed-ligand systems. A) Emission spectra of mixed-ligand systems prepared with varying BDC^{2-}/NH_2BDC^{2-} molar ratios, showing the evolution from Eu^{3+} emission (λ_{ex}= 285 nm, using a 450nm long-pass filter). Inset: Photograph of the $Tb_{00}Eu_{100}(BDC)_{00}(NH_2BDC)_{100}$@LP sample under 254 nm UV illumination. B) corresponding DLS analysis.

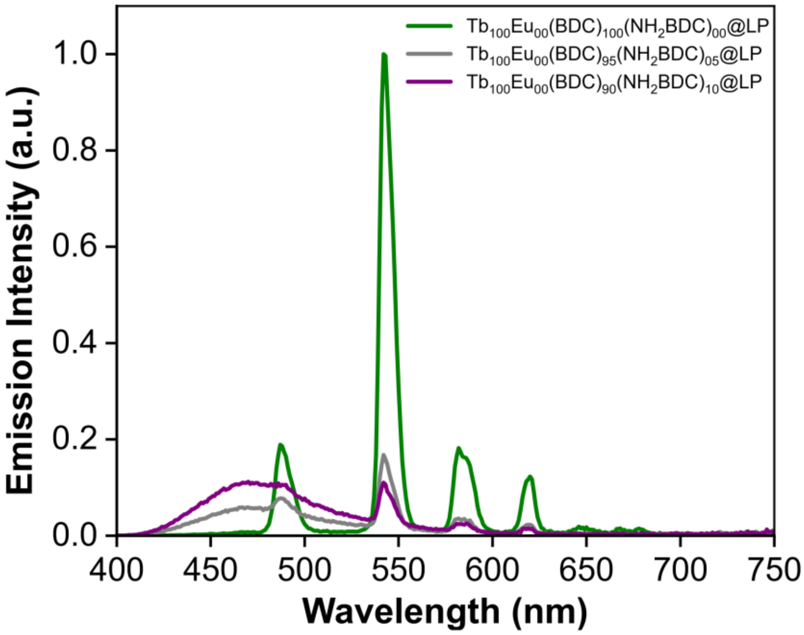

Figure S23: Progressive suppression of Tb emission in mixed-ligand systems. Emission spectra of mixed-ligand systems prepared with BDC^{2-}/NH_2BDC^{2-} molar ratios of 100/00, 95/05, and 90/10, showing the evolution from Tb^{3+} emission (λ_{ex}= 285 nm, using a 450nm long-pass filter).

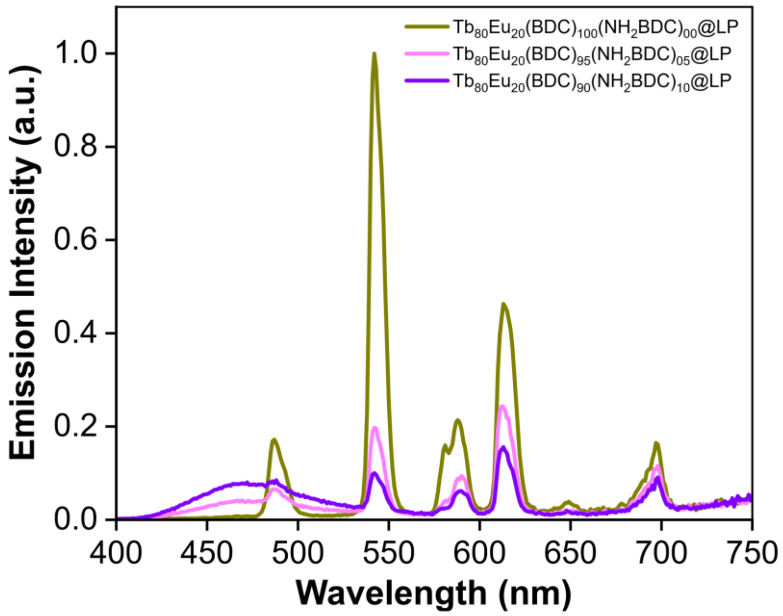

Figure S24: Evolution of Tb and Eu emission in mixed-ligand systems. Emission spectra of mixed-ligand systems prepared with BDC^{2-}/NH_2BDC^{2-} molar ratios of 100/00, 95/05, and 90/10, showing the evolution from Tb^{3+} and Eu^{3+} emissions (λ_{ex}= 285 nm, using a 450nm long-pass filter).

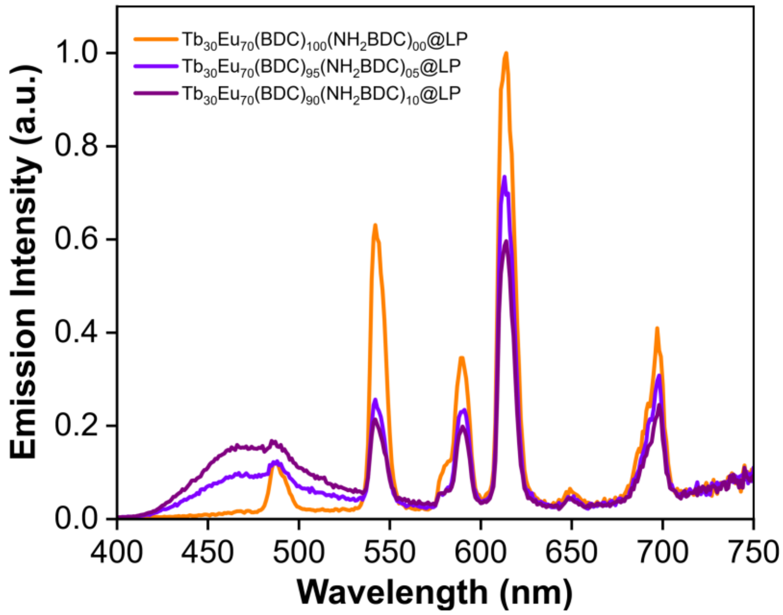

Figure S25: Evolution of Tb and Eu emission in mixed-ligand systems. Emission spectra of mixed-ligand systems prepared with BDC^{2-}/NH_2BDC^{2-} molar ratios of 100/00, 95/05, and 90/10, showing the evolution from Tb^{3+} and Eu^{3+} emissions (λ_{ex}= 285 nm, using a 450nm long-pass filter).

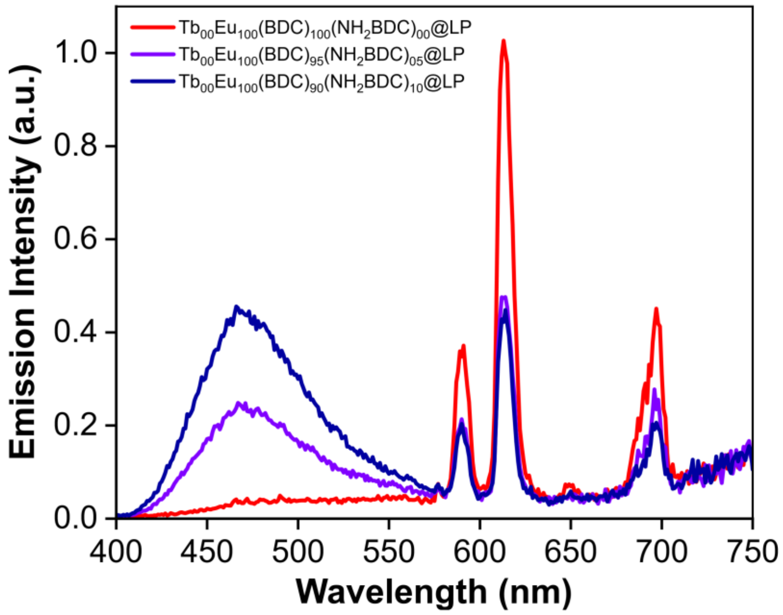

Figure S26: Progressive suppression of Eu emission in mixed-ligand systems. Emission spectra of mixed-ligand systems prepared with BDC^{2-}/NH_2BDC^{2-} molar ratios of 100/00, 95/05, and 90/10, showing the evolution from Eu^{3+} emission (λ_{ex}= 285 nm, using a 450nm long-pass filter).

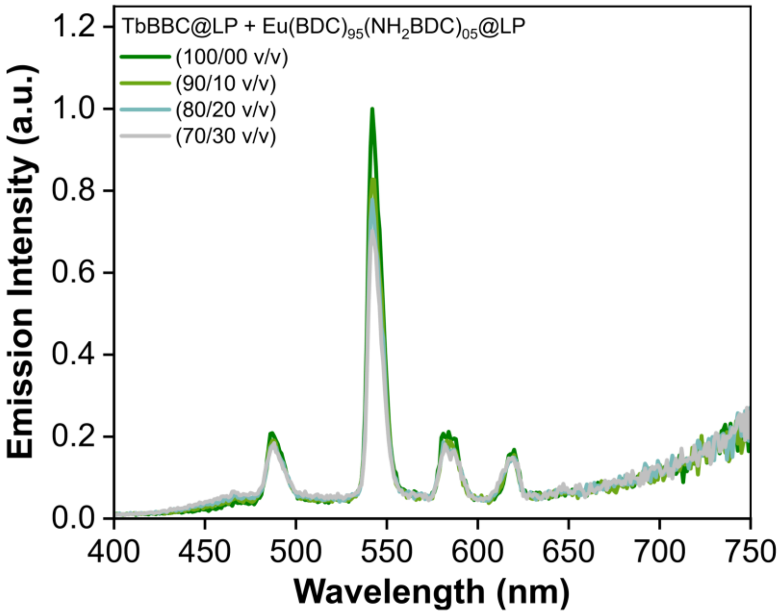

Figure S27: Emission spectra of physically mixed systems. Emission spectra of physical mixtures of $Tb_{100}Eu_{00}$BDC@LP and $Tb_{00}Eu_{100}(BDC)_{95}(NH_2BDC)_{05}$@LP prepared at v/v ratios of 100/00, 90/10, 80/20, and 70/30 (λ_{ex}= 285 nm, using a 450nm long-pass filter).

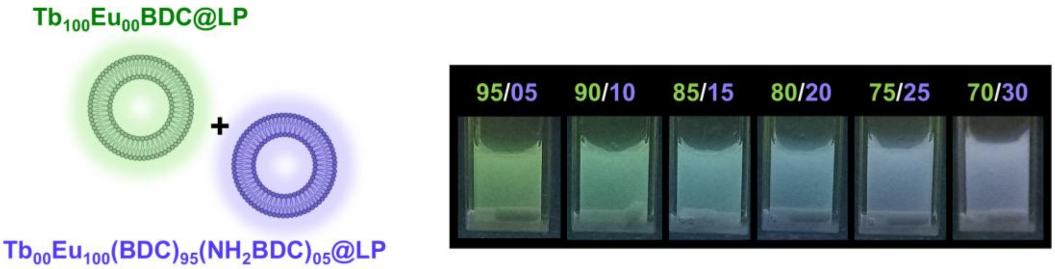

Figure S28: Colour tuning via physical mixing. Colour-tuning strategy based on physical mixtures of green-emissive $Tb_{100}Eu_{00}$BDC@LP and violet-emissive $Tb_{00}Eu_{100}(BDC)_{95}(NH_2BDC)_{05}$@LP, providing a route to cyan and near-white emission.

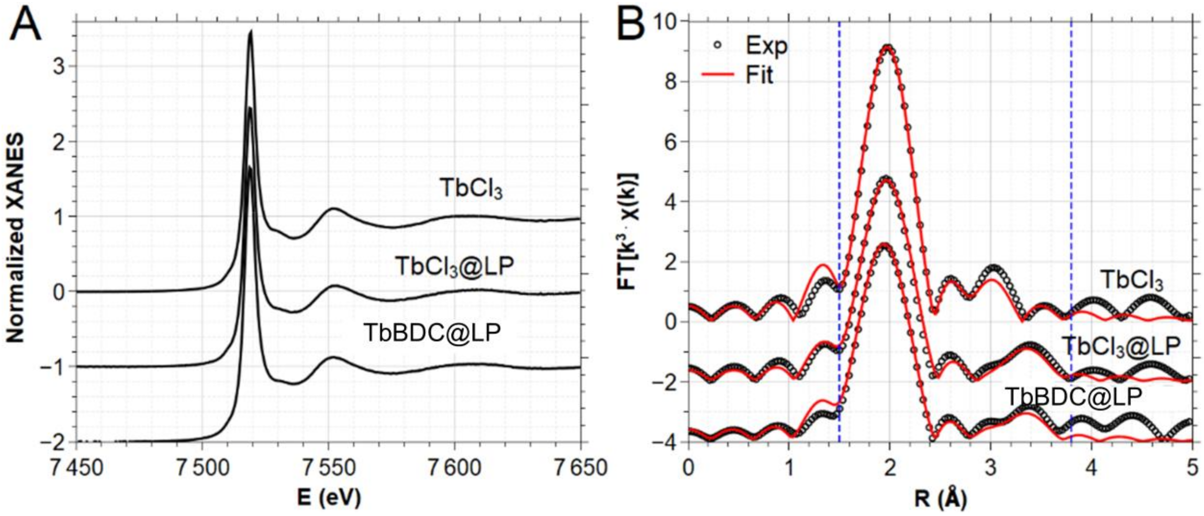

Figure S29: XANES and EXAFS analysis of the coordination environment of Tb in liposome nanoreactors. A) Experimental Tb L_{III}-edge XANES spectra of the $TbCl_3$ salt in solution (top), encapsulated in liposomes (middle), and co-encapsulated with BDC in liposomes (bottom). B) Experimental Fourier-Transformed EXAFS signal (open circles) of the same samples as in panel A, and theoretical curves (red solid) providing the best fit to the experimental data. Blue dashed vertical lines indicate the fitting region.

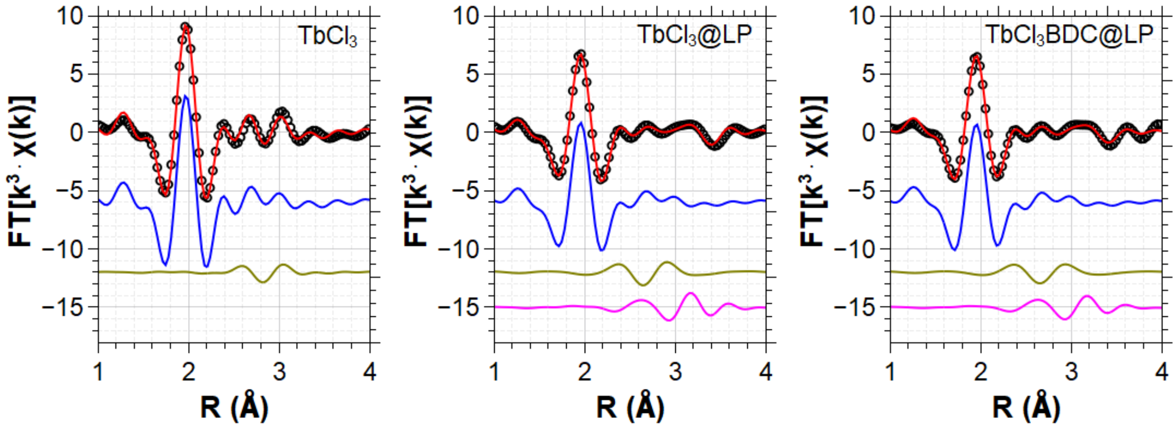

Figure S30: EXAFS fitting analysis. Real part of the Fourier-Transformed Tb L_{III}-edge EXAFS signal. Experimental spectra (open circles) and best-fitting curves (red) based on a theoretical model including Tb-O (blue), Tb-Cl (dark yellow), and Tb-Tb (magenta) contributions. The same model was applied to the three samples: $TbCl_3$ salt in solution (left), encapsulated in liposomes (middle), and co-encapsulated with BDC in liposomes (right).

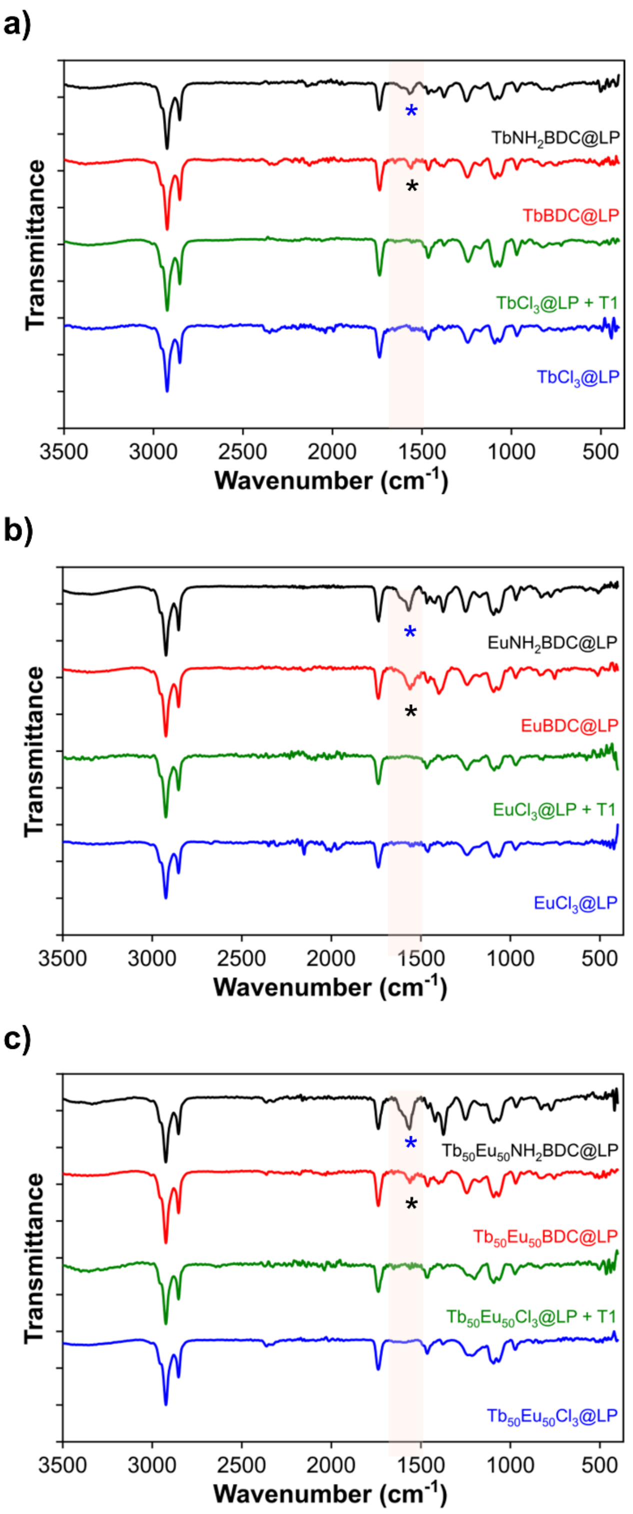

Figure S31: FT-IR analysis. FT-IR spectra comparing lanthanide-chloride and lanthanide-dicarboxylate systems encapsulated within liposomes: A) $TbCl_3$@LP, $TbCl_3$@LP + **T1**, TbBDC@LP, and $TbNH_2BDC$@LP; B) $EuCl_3$@LP, $EuCl_3$@LP + **T1**, EuBDC@LP, and $EuNH_2BDC$@LP; C) $Tb_{50}Eu_{50}Cl_3$@LP, $Tb_{50}Eu_{50}Cl_3$@LP + **T1**, $Tb_{50}Eu_{50}BDC$@LP, and $Tb_{50}Eu_{50}NH_2BDC$@LP. Blue and black stars indicate the asymmetric carboxylate stretching vibration ($\nu_{as}COO^-$) for NH_2BDC^{2-} and BDC^{2-}, respectively.

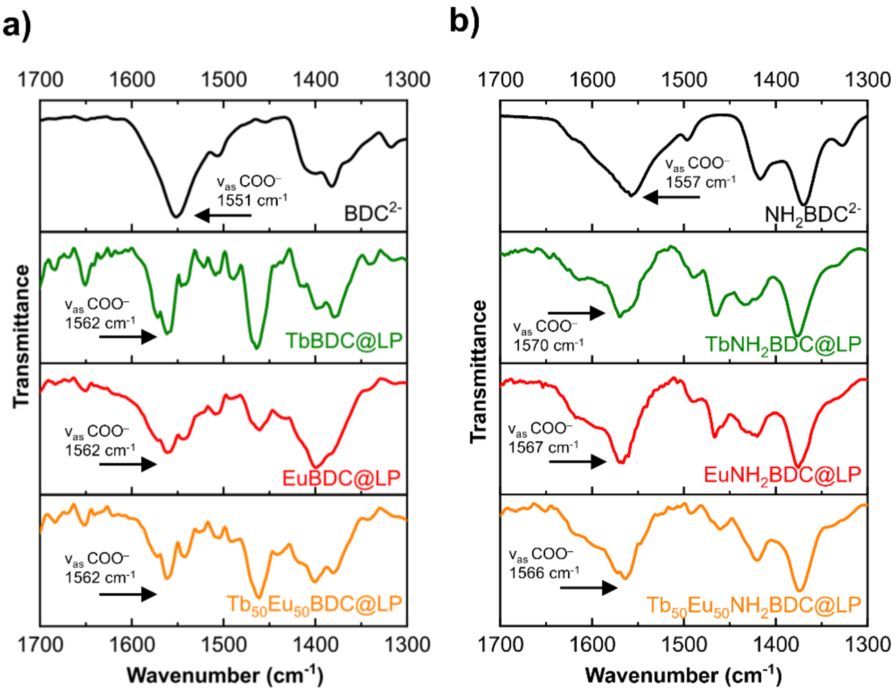

Figure S32: Shifts in asymmetric carboxylate stretching bands in FT-IR spectra. FT-IR spectra comparing the asymmetric carboxylate stretching vibration ($\nu_{as}COO^-$) of: a) TbBDC@LP, EuBDC@LP, $Tb_{50}Eu_{50}$BDC@LP, and the free BDC^{2-} ligand; and b) $TbNH_2$BDC@LP, $EuNH_2$BDC@LP, $Tb_{50}Eu_{50}NH_2$BDC@LP, and the free NH_2BDC^{2-} ligand.

SUPPLEMENTAL TABLE

Table S1: EXAFS-derived structural parameters. Structural and dynamical parameters of the average Tb binding site during the formation of TbMOFs in liposomes, extracted from fitting of Tb L_{III}-edge EXAFS data based on a theoretical model. The values of Tb-O, Tb-Cl, and Tb-Tb distances found in the literature for Tb^{3+} compounds are reported for comparison.[9]

Sample	Tb-O			Tb-Cl			Tb-Tb			ΔE_0 (eV)	R_{fit} (%)
	**N**	R (Å)	σ^2 (10^{-3} Å^2)	**N**	R (Å)	σ^2 (10^{-3} Å^2)	**N**	R (Å)	σ^2 (10^{-3} Å^2)		
**$TbCl_3$**	**6.4 ± 0.2**	2.397 ± 0.005	3.0 ± 0.4	**1.3 ± 0.4**	3.25 ± 0.03	10 ± 4	-	-	-	6.5 ± 0.7	0.65
**$TbCl_3$@LP**	**5.9 ± 0.2**	2.386 ± 0.007	6.1 ± 0.6	**3.5 ± 1.0**	3.10 ± 0.03	23 ± 5	**6.0 ± 1.7**	3.35 ± 0.02	20 ± 4	6.9 ± 0.6	0.75
**TbBCD@LP**	**5.8 ± 0.2**	2.376 ± 0.005	6.0 ± 0.4	**4.1 ± 1.0**	3.10 ± 0.02	27 ± 4	**6.7 ± 1.5**	3.34 ± 0.02	22 ± 2	5.8 ± 0.6	0.33
TbOCl[8]	4	2.24 ± 0.02	7 ± 1	5	3.07 ± 0.03	18 ± 3	4	3.55 ± 0.03	5 ± 2	4 ± 3	1.9
$TbCl_3{\cdot}6H_2O$[8]	6	2.38 ± 0.02	9 ± 1	2	2.73 ± 0.03	10 ± 3	-	-	-	4.2 ± 1.5	0.7

R_{fit} is the goodness of fit calculated as $\Sigma(\chi_{exp}-\chi_{fit})^2/\Sigma(\chi_{exp})^2$, where χ_{exp} is the experimental data point and χ_{fit} the corresponding point in the best-fitting curve.

SUPPLEMENTAL REFERENCES

(1) Torres-Huerta, A., Velásquez-Hernández, M.D.J., Tamarit-Amoros, E., Raschetti, M., Pinkas, D., Jurček, O., Pérez, J., and Valkenier, H. (2025). Spatiotemporal Control of the Formation of Luminescent Lanthanide Complexes in Liposome-Based Nanoreactors. *Angew. Chem. Int. Ed.*, e202510471. https://doi.org/10.1002/anie.202510471.

(2) Brouwer, A.M. (2011). Standards for photoluminescence quantum yield measurements in solution (IUPAC Technical Report). Pure Appl. Chem. *83*, 2213–2228. https://doi.org/10.1351/PAC-REP-10-09-31.

(3) Briois, V., Fonda, E., Belin, S., Barthe, L., La Fontaine, C., Langlois, F., Ribbens, M., and Villain, F. (2011). SAMBA: The 4–40 keV X-ray absorption spectroscopy beamline at SOLEIL. In UVX 2010 - 10e Colloque sur les Sources Cohérentes et Incohérentes UV, VUV et X ; Applications et Développements Récents (EDP Sciences), pp. 41–47. https://doi.org/10.1051/uvx/2011006.

(4) Landrot, G., and Fonda, E. (2025). *Fastosh* : a software for the treatment of XAFS datasets of environmental relevance or acquired in *operando* conditions. *J. Synchrotron Radiat. 32*, 1085–1094. https://doi.org/10.1107/S1600577525003923.

(5) Newville, M. (2013). Larch: An Analysis Package for XAFS and Related Spectroscopies. *J. Phys. Conf. Ser. 430*, 012007. https://doi.org/10.1088/1742-6596/430/1/012007.

(6) Rehr, J.J., Kas, J.J., Prange, M.P., Sorini, A.P., Takimoto, Y., and Vila, F. (2008). Ab initio theory and calculations of X-ray spectra. *Comptes Rendus Phys. 10*, 548–559. https://doi.org/10.1016/j.crhy.2008.08.004.

(7) Chong, S., Riley, B.J., and Nelson, Z.J. (2020). Dehydration synthesis and crystal structure of terbium oxychloride, TbOCl. *Acta Crystallogr. Sect. E Crystallogr. Commun. 76*, 621–624. https://doi.org/10.1107/S2056989020004387.

(8) Martinez-Gomez, N. C.; Vu, H. N.; Skovran, E. Lanthanide Chemistry: From Coordination in Chemical Complexes Shaping Our Technology to Coordination in Enzymes Shaping Bacterial Metabolism. *Inorg. Chem.* 2016, *55* (20), 10083–10089. https://doi.org/10.1021/acs.inorgchem.6b00919.

(9) Boglaienko, D.; Andersen, A.; Heald, S. M.; Varga, T.; Mortensen, D. R.; Tetef, S.; Seidler, G. T.; Govind, N.; Levitskaia, T. G. X-Ray Absorption Spectroscopy of Trivalent Eu, Gd, Tb, and Dy Chlorides and Oxychlorides. *J. Alloys Compd.* 2022, *897*, 162629. https://doi.org/10.1016/j.jallcom.2021.162629.
